# Hyperhomocysteinemia in ApoE-/- Mice Leads to Overexpression of Enhancer of Zeste Homolog 2 via miR-92a Regulation

**DOI:** 10.1371/journal.pone.0167744

**Published:** 2016-12-09

**Authors:** Yang Xiaoling, Zhao Li, Li ShuQiang, Ma Shengchao, Yang Anning, Ding Ning, Li Nan, Jia Yuexia, Yang Xiaoming, Li Guizhong, Jiang Yideng

**Affiliations:** 1 Basic Medical School, Ningxia Medical University, Key Laboratory of Cardio-Cerebro-Vascular Diseases, Ningxia Medical University, Yinchuan, Ningxia, China; 2 Institution of Medical Science of Ningxia, Yinchuan, Ningxia, China; University of Louisville, UNITED STATES

## Abstract

Hyperhomocysteinemia (HHcy) is an independent risk factor for cardiovascular diseases, such as atherosclerosis. HHcy promotes atherogenesis by modifying the histone methylation patterns and miRNA regulation. In this study, we investigated the effects of homocysteine (Hcy) on the expression of enhancer of zeste homolog 2 (EZH2), and tested our hypothesis that Hcy-induced atherosclerosis is mediated by increased EZH2 expression, which is regulated by miR-92a. The levels of EZH2 and H3K27me3 were increased in the aorta of ApoE-/- mice fed a high-methionine diet for 16 weeks, whereas miR-92a expression was decreased. Over-expression of EZH2 increased H3K27me3 level and the accumulation of total cholesterol and triglycerides in the foam cells. Furthermore, upregulation of miR-92a reduced EZH2 expression in the foam cells. These data suggested that EZH2 plays a key role in Hcy-mediated lipid metabolism disorders, and that miR-92a may be a novel therapeutic target in Hcy-related atherosclerosis.

## Introduction

Recent evidence has suggested that changes in the epigenetic mechanisms, such as DNA methylation [1], histone modifications [2], and micro RNA (miRNA) expression [3] contribute to the development of atherosclerosis. Homocysteine (Hcy), a toxic non-protein forming thiol-containing amino acid, is formed from methionine as a result of cellular methylation reactions [4]. Elevated plasma homocysteine levels are known to be a risk factor for atherosclerosis [5], resulting in cardiovascular diseases. It has been reported that cystathionine-β-synthase (CBS)-deficient mice present severe accumulation of tissue Hcy and protein arginine hypomethylation [6]. In addition, some miRNAs that regulate DNA methylation and acetylation are activated during hyperhomocysteinemia (HHcy) [7]. Hence, Hcy plays an important role in post-translational modifications by inhibiting the expression of key genes such as FABP4 [5] and ABCA1 [8]. However, the underlying mechanisms of epigenetic regulation in Hcy-induced atherosclerosis are poorly understood.

miRNAs belong to a class of highly-conserved small non-coding RNAs (~22 nucleotides), and are generated from 70–100 nucleotides hairpin precursors. The miRNAs post-transcriptionally regulate gene expression by binding to the 3’-untranslated regions (3’-UTR) of the mRNA transcripts, and eventually induce translational repression or transcript degradation. Depending on the function of the target gene products, miRNAs are involved in diverse biological processes, including cell survival, proliferation and apoptosis. Particularly, the importance of miRNA as a contributing risk factor in the pathogenesis of atherosclerosis has been well documented. Fang et al. demonstrated that endothelial miRNAs, namely miR-92a are differentially expressed between athero-susceptible aortic arch and nearby athero-protected descending thoracic aorta in normal swine [9]. In addition, miR188 has been shown to be involved in Hcy-induced cardiac remodeling [10]. Furthermore, our previous study also proves that miR-124 is involved in Hcy-induced atherosclerosis [11]. These studies indicate that miRNAs have emerged as important pathophysiological mediators of the vascular system. Moreover, miRNAs have also been closely correlated with other epigenetic mechanisms, such as histone modifications that together contribute to the pathogenesis of atherosclerosis [12, 13].

Histone modifications mainly include acetylation, methylation, phosphorylation, ubiquitination, sumoylation, ADP-ribosylation, deamination and proline isomerization. Histone methylation is one of the well-studied modifications, which is mediated by histone methyltransferase and associated with activation or repression of gene transcription. The H3 histone tail of amino acids mostly comprise of lysine and arginine [14]. Trimethylation of histone H3 at lysine 27 (H3K27me3) is an epigenetic mark associated with gene silencing, and often found in the promoter of developmental genes [15]. Enhancer of zeste homolog 2 (EZH2) histone methyltransferase, a catalytic subunit of Polycomb repressive complex 2 (PRC2), is responsible for catalyzing the methylation of H3K27, thereby resulting in gene silencing [16]. EZH2 plays an essential role in epigenetic maintenance and has been implicated in regulating multiple cellular processes during certain diseases [17], such as cancer and atherosclerosis [18]. However, studies reporting the mechanisms of HHcy in atherogenesis via histone methylation are limited. Recent findings have provided evidence that endogenous miRNAs that target gene promoters form epigenetic remodeling complexes and suppress gene expression by fostering histone methylation (H3K27) [19]. In addition, EZH2 is regulated at the posttranscriptional level by the aberrant expression of miRNAs [20]. However, the role of miRNAs in EZH2 regulation in Hcy-induced atherosclerosis has not yet been fully elucidated.

Hcy is known to be associated with aberrant methylation, therefore the role of epigenetic mechanisms in atherosclerosis need to be further explored. Hence, the main aim of this study was to elucidate the epigenetic mechanisms comprising miRNA vs. histone methylation in atherosclerosis under elevated levels of Hcy.

## Material and Methods

### Animals and samples collection

Six-week-old male apolipoprotein-E-deficient (ApoE-/-) mice (strain C57BL/6J; Animal Center of Peking University, Beijing, China, Permit Number: SCXK2002-001) were divided into 3 groups (n = 10 each) as following: (1) Hyperlipidemia (HLP) group: mice were fed standard mouse diet containing 0% cholesterol, 5.23% fat, 0.37% methionine, 2.39 mg/g choline, 3.19 mg/kg folate, 54.6 μg/kg B12 and 14.5 mg/kg B6; (2) HHcy group: mice were fed standard mouse diet plus 1.7% methionine; (3) HHcy+folate+vitamin B12 (HHcy+FA+VB) group: mice were fed standard mouse diet with 1.7% methionine, folate and vitamin B12 supplements. The control group (CON) consisted of wild-type (WT) C57BL/6J mice fed with a regular mouse diet. The animals were housed in a temperature-controlled room (22–25°C, 45% humidity) with a 12 h dark-light cycle for 16 weeks. All the animal experiments conformed to the Animal Management Guidelines of the People’s Republic of China. The experimental protocol was approved by the Animal Care Ethics Committee of the Ningxia Medical University.

The mice were sacrificed after 16 weeks, and blood was withdrawn in a fasted state from the orbital sinus of anesthetized animals. Serum was then isolated from the blood, and the levels of Hcy and lipid profiles, including total cholesterol (TC), triglyceride (TG), high-density lipoprotein (HDL) and low density lipoprotein (LDL), were determined by High Performance Liquid Chromatography (HPLC) and Automatic Biochemical Analyzer (SIEMENS, Munich, Germany), respectively. The thoracic aorta was isolated from the mice for hematoxylin and eosin (HE) as well as Oil Red O staining. The plaqe area was quantified using the Histolab software (Microvisions, Paris, France). At the end of the experimental protocol, mice were sacrificed by cervical dislocation, and thoracic aorta were rapidly excised and frozen in liquid nitrogen.

### Hematoxylin-Eosin (HE) and Oil Red O staining

Formalin-fixed tissues were embedded in paraffin using standard procedures. Aortic tissues from ApoE-/- and WT mice were placed in OCT (VWR Scientific, St. Louis, MO), and rapidly frozen for sections. The thoracic aortasections (4 μm thick) were cut and stained with HE (Bi Yuntian, Shanghai, China) for standard microscopy.

For Oil Red O staining, sections were fixed in 10% formalin for 1 h, and rinsed 3 times with ddH_2_O. Sections were dehydrated for 5 min in 100% isopropanol, stained with filtered Oil Red O (Sigma, St. Louis, MO, USA) working solution (60% Oil Red O stock solution/40% water) for 1 h at room temperature, and then rinsed in ddH_2_O for 5 min. The sections were observed under the inverted microscope and the images were captured using BD2000 microscopic image analysis system (Qihang, Beijing, China).

### Cell treatment and transfection

Human THP-1 cells, a human monocytic leukemia cell line, was maintained in RPMI-1640 (GIBCO, California, USA) medium supplemented with 10% fetal bovine serum (GIBCO), 100 U/mL penicillin and 100 μg/ mL streptomycin (GIBCO) in a humidified atmosphere at 37°C, 5% CO_2_. Cells were routinely passaged at 2–3 day intervals. To demonstrate the effect of Hcy on lipogenesis, cells were initially differentiated into macrophages by adding 5 μM phorbol 12-myristate 13-acetate (PMA; Sigma-Aldrich, St Louis, USA) for 48 h. Thereafter, macrophages were transformed into foam cells by incubation with 50 mg/L oxidized low density lipoprotein (ox-LDL; Sigma-Aldrich, St Louis, USA) in medium containing 100 μM Hcy or supplemented with its antagonist. Total cholesterol (TC) and triglycerides (TG) were analyzed by Cholesterol Quantitation Kit (Catalogue No. K603-100, Biovision, Inc, Mountain View, CA, USA). For transfection experiments, the cells were seeded in 6-well plates, incubated overnight, and then transfected with Ad-EZH2, Ad-EZH2-siRNAs or Ad-GFP (GENECHEM, Shanghai, China) using Lipofectamine 2000 (Invitrogen, California, USA) according to the manufacturer’s instructions.

### RNA extraction and quantitative real-time PCR (qRT-PCR)

Total RNA was isolated from the aorta tissue and foam cells using RNA isolation kit (Invitrogen) according to the manufacturer’s protocol. The RNA samples were accurately quantified using Qubit fluorometer (Invitrogen), and immediately converted into cDNA using the reverse transcription kit (Takara, Dalian, China). The cDNA was amplified by PCR in a real time cycler using FastStart SYBR Green master mix (MBI, Vilnius, Lithuania) and primers for EZH2 and β-actin (Shenggong, Shanghai, China). The primers we used as follow. Human EZH2 primers: positive-sense: 5’- CATTCGGTAAATCCAAACTGC -3’, anti-sense: 5’- CGACATACTTCAGGGCATCA -3’. Mouse EZH2 primer: positive-sense: 5’- GTATGACTGCTTCCTACATCCCTTC -3’, anti-sense: 5’- CTGGGCGTTTAGGTGGTGTCT-3’. The primers of β-actin were synthetise by commercial company (Shenggong, Shanghai, China). The SYBR Green PCR cycle was set as following: initial denaturation cycle for 10 min at 95°C, 45 cycles consisting of 30 s of denaturation at 95°C, 30 s annealing at primer-specific temperatures, and extension at 72°C. All the experiments were performed in triplicates, and the data were normalized using β-actin as the house-keeping gene, and expressed as mean ± SD.

### Western blot analysis

The protein was extracted from the aorta tissue and foam cells in lysis buffer containing the protease inhibitor cocktail (Biyuntian, Shanghai, China). Western blot analysis was performed as we described previously [5]. In brief, protein concentration was quantified using the BCA protein assay (Pierce, Rockford, IL), and 20 μg of protein extracts were loaded onto 10% SDS-PAGE for electrophoresis. The protein was then transferred onto a PVDF-membrane (Millipore, Boston, Massachusetts, USA) using a Trans-Blot SD Semi-Dry Transfer Cell (Bio-Rad, Berkeley, California, USA), and blocked overnight using 5% nonfat dry milk and Tris-buffered saline with 0.1% Tween 20 (TBS-T). The membrane was incubated with anti-EZH2, H3K27me1,2,3 monoclonal antibody (1:1000 dilution; Abcam, Cambridge, MA, USA) and anti-β-actin antibody (1:1000 dilution; Zhongshan Biotech, Beijing, China) followed by HRP-labeled goat anti-mouse IgG (1:2500 dilution; Santa Cruz Biotechnology, Inc., Santa Cruz, CA, USA). Protein was then detected using the chemiluminescence kit (Biyuntian, Shanghai, China). All samples were derived at the same time and processed in parallel.

### Immunofluorescence staining of adipose differentiation-related protein (ADRP)

First, cells in slides were washed with PBS, fixed with 4% paraformaldehyde for 30 min, then refrigerated in PBS until further use. Immediately prior to staining, the cells were washed with PBS, permeabilized with PBS containing 0.05% Triton X-100 and 10% goat serum for 5 min, and then incubated with anti-ADRP antibody (1:200 dilution; Abcam, Cambridge, MA, USA) overnight at 4°C. Next day, the cells were washed with PBS, incubated with a fluorescein isothiocyanate (FITC)-conjugated donkey anti-rabbit IgG (1:1000 dilution; Abcam, Cambridge, MA, USA) for 1 h at 37°C, followed by washing with PBS for three times, and the nuclei were then counterstained with 4, 6-diamidino-2-phenylindole (DAPI, Sigma, St. Louis, MO, USA). Bound antibodies were viewed under a confocal microscope (Olympus Fluoview 1000, Japan).

### Vector construction of Lv-miR-92a

miR-92a pri-miR sequences were amplified and the amplified PCR product was cloned into hU6-MCS-PGK-EGFP vector (LV-miR-92a)(Hanbio, Shanghai, China). Empty lentiviral vector was used as the negative control. Design the antagonism of miR-92a and cloned the sequences into lentiviral vector (LV-miR-92a-antago). The sequence of antagonism is GACAGGCCGGGACAAGTGCAATATTTTTTc. LV-miR-92a and LV-miR-92a-antago were transfected into THP-1 cells to screen stable cell lines by puromycin.

### Luciferase reporter assay

Through TargetScan, a bioinformatics prediction program that can identify hypothetical mRNA-binding-specific miRNA, we identified EZH2 as a potential target of miR-92a. Genomic DNA extracted from THP-1 cells was used as a template to amplify a fragment of the 3’-UTR of EZH2, which contains the putative miR-92a-binding sequence. The amplified PCR product was cloned into PmirGLO dual fluorescent reporter system vectorvector (Promega, Madison, WI, USA). The miR-92a binding site within the EZH2 3’UTR was mutated using PCR-based site-directed mutagenesis. A control reporter plasmid with a mutation of 6 nucleotides corresponding to miR-92a seed sequence (ACGUUAU) was constructed by site-directed mutagenesis. The luciferase activity was examined at 48 hour post-transfection using a Dual-Luciferase Reporter Assay System (Promega, Madison, Wisconsin, USA) and a 96-well plate reader (Thermo Scientific, Waltham, Massachusetts, USA). All the experiments were repeated at least 3 times.

### Statistical Analyses

Statistical analyses were performed using GraphPad Prism 4.03 Software (San Diego, USA). Data were analyzed for differences between the dietary treatment groups by either One-way ANOVA or unpaired Student's *t*-test. Correlation analysis was performed using the Pearson correlation analysis. *P* ≤ 0.05 was considered as statistically significant. Results were expressed as mean ± SD. All the experiments were performed at least 3 times, and representative results were shown.

## Results

### Hcy promotes atherosclerosis progression

**Fig 1:**
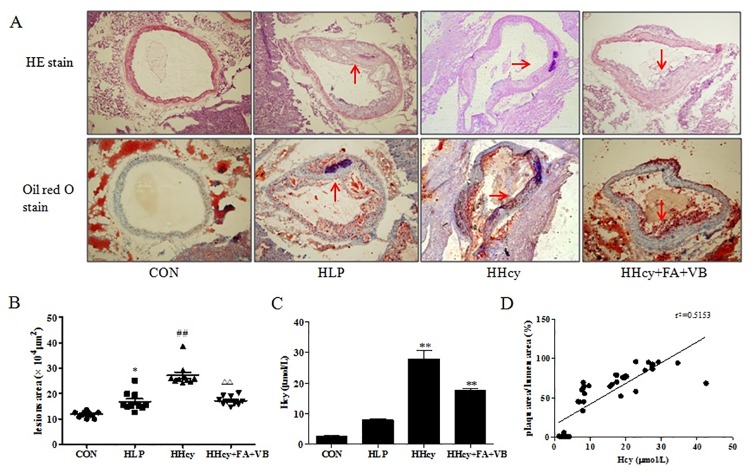
Hcy promoted atherosclerosis progression in ApoE-/- mice. ApoE-/- and control mice were fed a regular, methionine or folate diet, and after 16 weeks, serum and thoracic aorta were isolated. (A) Hematoxylin and eosin (H&E) and Oil Red O staining of the thoracic aorta sections (10×10). The original magnification was × 400. The red arrow indicates a plaque region. (B) Quantification of the average plaqe area was performed using the Histolab software. Representative Oil red O staining of the thoracic aorta sections of the mice (n = 5 to 7 sections per mice) were shown and the plaque area was presented as lesion % (% of whole aorta). Each symbol represented one animal and the horizontal bar indicated the mean value. (C) The serum from ApoE-/- and control mice were analyzed for total Hcy levels. (D) Positive correlation of the atherosclerotic plaque area with Hcy levels was examined by Pearson correlation analysis. All results were presented as mean ± SD. * *P* < 0.05, ** *P* < 0.01, vs. CON. #*P* < 0.05, ##*P*< 0.01, vs. HLP. Δ*P* < 0.05, ΔΔ*P* < 0.01, vs. HHcy. CON: WT C57BL/6J mice were fed with a regular mouse diet; HLP: ApoE-/-mice were fed standard mouse diet; HHcy: ApoE-/- mice were fed standard mouse diet plus methionine; HHcy+FA+VB: mice were fed standard mouse diet with methionine, folate and vitamin B12 supplements.

Atherosclerosis is a systemic chronic disease affecting the vascular walls of the arteries. To determine the order of severity of atherosclerosis, it is important to assess the plaque area, a well-known indicator. Foam cells, the most abundant cells within the lesions, are implicated in the pathogenesis of atherosclerosis. In order to quantify foam cells within the plaques, we performed Oil Red O staining, which is a lysochrome diazo dye used for staining neutral triglycerides and lipids. Thus, H&E and oil Red O staining of the thoracic aorta of mice were used to evaluate the size of the atherosclerotic lesions. The analysis of the H&E staining revealed large atherosclerotic plaques in the thoracic aorta of the ApoE-/- mice, whereas there was no evidence of plaque formation in the control mice. Apo E-/- mice fed with methionine was a typical method to replicate hyperhomocysteinemia animal model [5].Folic acid and vitamin B12 interfered Hcy production of methionine cycle [4, 8]. Increased lesions were observed in the HHcy group compared to the HLP group; moreover, when compared to HHcy+FA+VB group, the HHcy group demonstrated increased lesions (Fig 1A and 1B). In addition, HHcy group displayed markedly increased plaqe area compared to HLP group. The area of the plaque in HHcy+FA+VB group was significantly lower than the HHcy group (Fig 1A and 1B). Consistent with the results from the H&E staining, thoracic aorta from the HHcy group had increased accumulation of lipid droplets compared with the control and HLP group, as determined by Oil Red O staining (Fig 1A). The area of the atherosclerotic plaqe in HLP and HHcy groups also increased, compared to the control group (Fig 1B, *P* < 0.05).

HHcy was induced by feeding ApoE-/- mice a high-methionine diet for 16 weeks, and serum Hcy concentrations were measured by HPLC. The results showed that serum Hcy concentrations in the HHcy group were 9.62-fold higher than the control group (*P* < 0.01), and 2.62-fold higher than the HLP group (Fig 1C). In HHcy+FA+VB group, the Hcy concentration was 37% lower than the HHcy group. Therefore, high-methionine diet significantly increased the serum concentrations of Hcy, suggesting that the diet-induced HHcy model was successful. To address the relationship of Hcy with that of the atherosclerotic plaque area, we calculated the correlation between them using Pearson correlation analysis. As expected, the analysis showed a strong and significantly positive correlation between the serum concentrations of Hcy and the plaque area (R^2^ = 0.5153, *P* < 0.01). These results suggest that elevated Hcy levels may aggravate the formation of the atherosclerotic plaque (Fig 1D).

### Hcy increases the lipid levels in ApoE-/- mice and accelerates the accumulation of lipids in the macrophages

**Fig 2:**
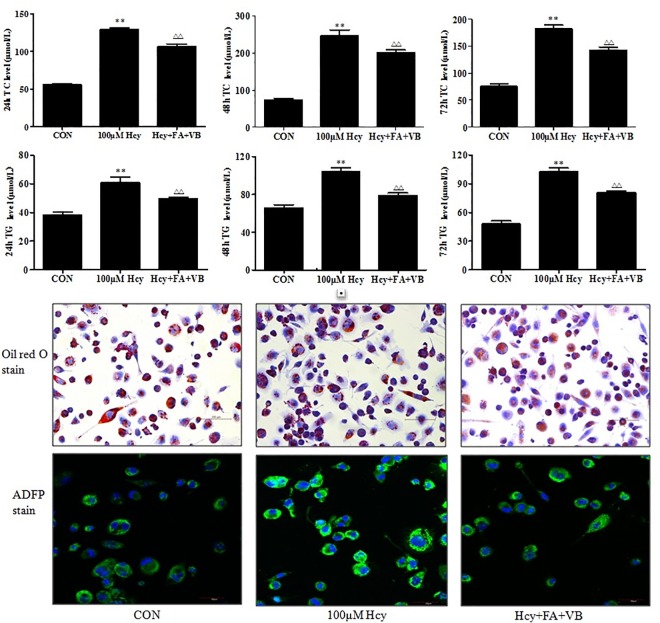
Hcy increased lipid accumulation of macrophage foam cell. Human macrophage foam cells were incubated with 100 μM Hcy or folate supplements for 24, 48 or 72 h. TC and TG levels in cells were quantified. Lipid droplets were observed by oil red O stain. The results were derived from triplicate experiments. Data were presented as mean ± SD. *P < 0.05, **P < 0.01, vs. CON. #*P* < 0.05, ##*P*< 0.01, vs. 100 μM Hcy. CON: WT C57BL/6J mice were fed with a regular mouse diet; HLP: ApoE-/-mice were fed standard mouse diet; HHcy: ApoE-/- mice were fed standard mouse diet plus methionine; HHcy+FA+VB: mice were fed standard mouse diet with methionine, folate and vitamin B12 supplements.

Lipid metabolism disorder is an important feature of atherosclerosis. As shown in Table 1, TC, TG, and LDL were increased, and HDL was decreased after administration of different diets. Lipid profiling suggested that high-methionine diet disturbed lipid metabolism, which was consistent with study described previous. Lipid-laden macrophages, also known as foam cells, play a crucial role in the initiation and progression of atherosclerosis. To investigate the effects of Hcy on macrophage lipid accumulation, we performed assays to quantify the levels of TC and TG in macrophage foam cells. The results demonstrated that the levels of TC increased when the foam cells were treated with 100 μM Hcy for 24, 48 and 72 h (*P* < 0.01, Fig 2). Folate and vitamin B12 supplementation effectively reversed this effect of Hcy, and significantly reduced the levels of TC in the foam cells (*P* < 0.01; Fig 2). Moreover, the levels of TG were significantly higher in the Hcy group compared to the control group (*P* < 0.01; Fig 2). Furthermore, folate and vitamin B12 supplementation alleviated the levels of increased TG that were induced by Hcy (*P* < 0.01, Fig 2). Oil red O and ADFP stain of foam cells also showed Hcy accelerate lipid deposit (Fig 2). Based on these results, we concluded that Hcy promoted lipid accumulation in macrophage foam cells.

**Table 1:** TC, TG, HDL and LDL levels in mice (mean ± SD, n = 10)

Gorup	TC (mmol/L)	TG (mmol/L)	HDL (μmol/L)	LDL (μmol/L)
CON	2.06 ± 0.1	0.31 ± 0.07	1.28 ± 0.05	0.41 ± 0.01
HLP	15.83 ± 0.46*	1.18 ± 0.22*	0.63 ± 0.05*	0.79 ± 0.07
HHcy	24.02 ± 0.80^#^	1.22 ± 0.26^#^	0.48 ± 0.03^#^	1.88 ± 0.42^#^
HHcy+FA+VB	19.53 ± 0.65^Δ^	0.83 ± 0.12^Δ^	0.55 ± 0.02^Δ^	1.07 ± 0.06^Δ^

Results were presented as mean ± SD. After 16 weeks of feeding, blood was collected and serum lipids were detected by an automatic biochemistry analyzer.

*P < 0.05, *vs*. CON.

#*P* < 0.05, vs. HLP.

Δ*P* < 0.05, vs. HHcy. TC, total cholesterol; TG, triglycerides; HDL, high-density lipoprotein; LDL, low-density lipoprotein.

### Hcy increase the H3K27me3 level

**Fig 3:**
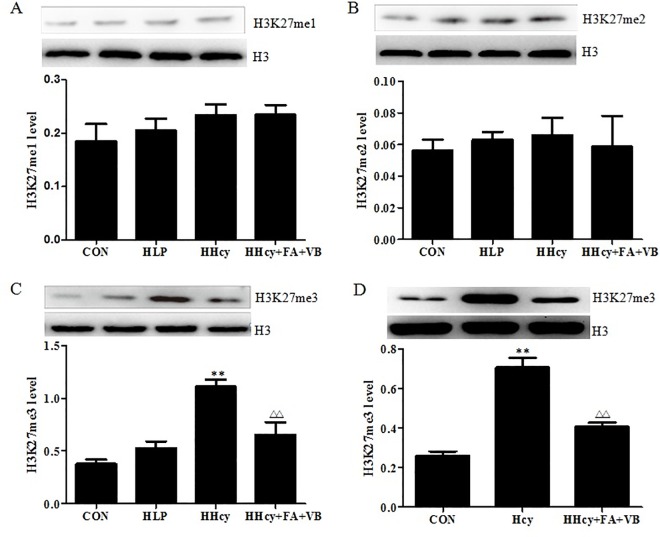
Hcy increased H3K27me3 level. (A) H3K27me1, 2, 3 levels in the mice were detected by Western blot. (B) H3K27me3 level was detected in macrophage foam cells by Western blot. Signal intensity of H3K27me1, 2, 3 was quantified by densitometric analysis and normalized to pan H3 control. Data were presented as mean ± SD. *P < 0.05, **P < 0.01, vs. CON. ##*P*< 0.01, vs. HLP. Δ*P* < 0.05, ΔΔ*P* < 0.01, vs. HHcy or 100 μM Hcy. CON: WT C57BL/6J mice were fed with a regular mouse diet; HLP: ApoE-/-mice were fed standard mouse diet; HHcy: ApoE-/- mice were fed standard mouse diet plus methionine; HHcy+FA+VB: mice were fed standard mouse diet with methionine, folate and vitamin B12 supplements.

Several studies have demonstrated that histone modification plays a vital role in atherosclerosis[18]. Hcy, a risk factor for atherosclerosis, is known to aggravate the progression of atherosclerosis via histone modification[5]. Thus, in order to explore the possible epigenetic mechanisms for Hcy-related pathogenesis of atherosclerosis, we assessed the levels of H3K27me1, 2, and 3 in both the aortic tissue and the foam cells. As shown in Fig 3, no differences were observed in the levels of H3K27me1 and 2 amongst different mice groups (Fig 3A and 3B). Interestingly, the levels of H3K27me3 in the HHcy group were 194% (*P* < 0.05) and 110% (*P* < 0.05) higher than the control and HLP groups, respectively (Fig 3C and 3D). Treatment with folate and vitamin B12 significantly decreased the level of H3K27me3. Consistent with the data from the *in vivo* experiments, the levels of H3K27me3 level increased to 263% compared to the control group, when the foam cells were treated with 100 μM Hcy for 48 h. However, the H3K27me3 level in the Hcy+FA+VB group decreased significantly to 71% (*P* < 0.05) relative to the Hcy group (Fig 3E and 3F). These results indicated that H3K27me3 was involved in the progression of atherosclerosis.

### Upregulated EZH2 increases H3K27me3 level and lipid accumulation

**Fig 4:**
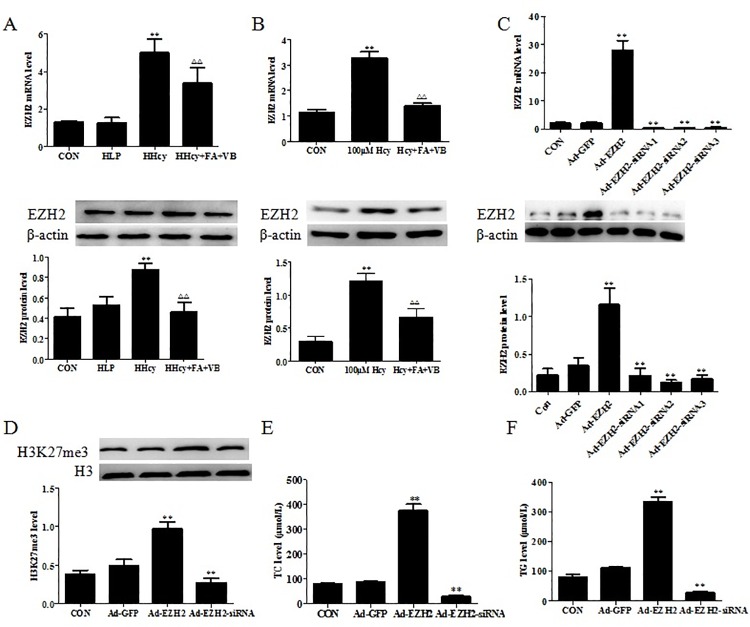
Upregulated EZH2 increased H3K27me3 level and lipid accumulation. (A) RNA and protein were isolated from the artery of ApoE-/- and control mice, and the expression of EZH2 was detected by qRT-PCR and western blot. (B) Macrophage foam cells were treated with 100 μM Hcy or folate for 48 h, RNA and protein were isolated from the cells, and the expression of EZH2 was quantified. (C) EZH2 mRNA and protein expression in macrophage foam cells transfected with Ad-EZH2 or Ad-EZH2-siRNA. (D) Western blot analysis for H3K27Me3 and pan H3 control in macrophage foam cells transfected with Ad-EZH2 or Ad-EZH2-siRNA. (E, F) After 48 h incubation with 100 μM Hcy and folate, macrophage foam cells were lysed, and intracellular TC and TG were measured. The results were derived from triplicate experiments. Data were presented as mean ± SD. **P < 0.01, vs. CON. ΔΔ*P* < 0.01, vs. HHcy or 100 μM Hcy.

EZH2, a histone methyltransferase, initiates target gene silencing by catalyzing the of H3K27me3 level, and eventually leading to chromatin condensation [21]. In order to confirm whether Hcy affected the expression of EZH2, qRT-PCR and Western blotting were used to detect the mRNA and protein levels of EZH2, respectively, in both mice and foam cells. The qRT-PCR analysis revealed markedly elevated EZH2 mRNA levels in the aortic tissue of ApoE-/- mice fed a high-methionine diet (Fig 4A). However, EZH2 mRNA levels in the HHcy+FA+VB group decreased significantly. In addition, western blot analysis was performed to determine whether increased EZH2 gene expression also led to an increase in the protein levels of EZH2. Similar to the qRT-PCR results, the levels of EZH2 protein in the aortic tissue of HHcy group increased significantly compared with the control and HLP groups (Fig 4A). Protein expression of EZH2 in the HHcy+FA+VB group was lower than the HHcy group. Exposure of foam cells to Hcy resulted in induced expression of EZH2 both at the transcriptional and translational levels (Fig 4B). When the cells were treated with folate and vitamin B12 for 48 h, the mRNA and protein levels of EZH2 reduced significantly (Fig 4B). These results indicated that Hcy regulated EZH2 mRNA and protein expression, similar to H3K27me3.

To further elucidate the functions of EZH2, we transfected Ad-EZH2 or Ad-EZH2-siRNAs into the foam cells, and evaluated the transfection efficiency. After the cells were transfected with adenovirus vectors or siRNAs, enhanced green fluorescence was assayed. The cells transfected with Ad-EZH2 demonstrated increased levels of EZH2 mRNA and protein, which were greater than 12-fold and 4.3-fold, respectively, relative to control Ad-GFP (Fig 4C). Similarly, transfection with Ad-EZH2-siRNA1, Ad-EZH2-siRNA2 and Ad-EZH2-siRNA3 to knock down EZH2, resulted in reduced EZH2 expression at both the transcript and the protein levels (Fig 4C).

To confirm whether EZH2 specifically regulated H3K27me3, the level of H3K27me3 was further assessed. Overexpression of EZH2 in the foam cells resulted in the upregulation of H3K27, whereas EZH2 knockdown downregulated the expression H3K27me3 protein (Fig 4D). The data suggested that EZH2 was a key enzyme in the regulation of H3K27me3.

Although EZH2 is considered a functional oncogene, and strongly associated with the progression and invasion of certain cancers [20], the role of EZH2 in lipid metabolism has been seldom reported. To determine whether altered EZH2 expression was involved in lipid accumulation, the concentrations of TG and TC in the foam cells were determined. As shown in Fig 4E and 4F, overexpression of EZH2 in foam cells increased the levels of TC and TG by 3.6- and 3.1- fold, respectively. Contrarily, EZH2 knockdown by RNA interference resulted in 64% and 66% decrease in the levels of TC and TG, respectively, suggesting that EZH2 contributed to the accumulation of lipid in the foam cells.

### 3’-UTR of EZH2 transcript is a direct target for miR-92a

**Fig 5:**
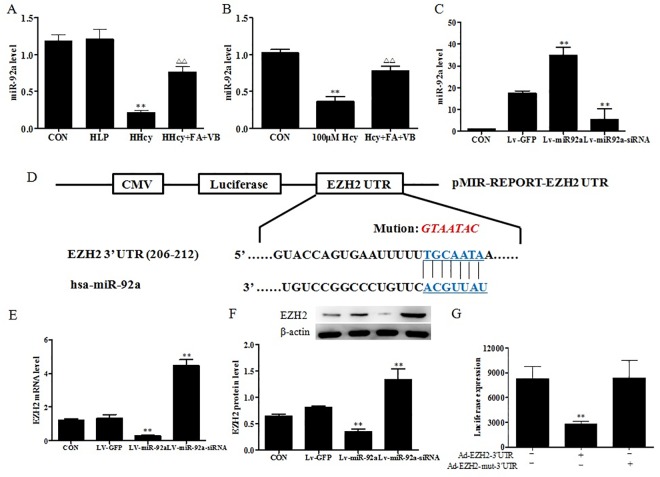
miR-92a was involved in the atherosclerosis process and targeted the 3’-UTR of EZH2. (A, B) Total RNA was isolated from the artery of mice and macrophage foam cells, and miR-92a expression was assessed by qRT-PCR. (C) Ad-EZH2 was transfected into macrophage foam cells, and the expression of miR-92a was assessed by qRT-PCR. (D) Predicted binding site for miR-92a in the 3’UTR region of human EZH2 mRNA. Mutant-type pMIR vector was inserted with mutated seed sequence (from TGCAATA to GTAATAC) for miR-92a. (E, F) Lv-miR-92a or Lv-miR-92a-siRNA was transfected into macrophage foam cells, and the expression of EZH2 mRNA and protein were assessed by qRT-PCR and western blot analysis, respectively. (G) Luciferase activities after co-transfection with control or miR-92a, and wild-type or mutant-type pMIR vectors with the indicated 3’UTR of EZH2. The results were derived from triplicate experiments. Data were presented as mean ± SD. **P < 0.01, vs. CON. ΔΔ*P* < 0.01, vs. HHcy or 100 μM Hcy.

The different roles of miRNAs have been studied in animal models of atherosclerosis, and found to be associated with HHcy. We tested the mRNA expression of miR-92a by qRT-PCR. The results showed that there were no significant differences in the levels of miR-92a between the control and HLP groups. However, HHcy group had significantly reduced miR-92a levels compared to the HLP group. The expression of miR-92a in the HHcy+FA+VB group was higher than the HHcy group (Fig 5A). Consistent with the results from the animal experiments, exposure of foam cells to Hcy resulted in decreased miR-92a levels (Fig 5B). Treatment of the foam cells with folate and Vitamin B12 for 48 h induced the expression of miR-92a (Fig 5B). The data suggest that miR-92a may be involved in the development of HHcy.

In general, miRNAs bind to the corresponding target mRNAs, and regulate gene transcription. Using the bioinformatics software TargetScan (http://www.targetscan.org) for target gene prediction, EZH2 was identified as one of the potential targets of miR-92a. Fig 5D illustrates the predicted binding site of miR-92a with EZH2 3’UTR. To further confirm the direct interaction of miR-92a with EZH2, we first transfected LV-miR-92a and Lv-miR92a-siRNA into the foam cells, and evaluated EZH2 mRNA and protein expression. As shown in Fig 5C, the relative expression levels of miR-92a in Lv-miR-92a group increased significantly compared with the control group. However, there was a significant reduction in the level of miR-92a in Lv-miR92a-siRNA group. As expected, Lv-miR-92a significantly reduced both EZH2 mRNA and protein levels, whereas Lv-miR92a-siRNA accelerated the production of EZH2 mRNA and protein (*P* < 0.05, Fig 5E and 5F). Lastly, we constructed Ad-EZH2 3’-UTR and Ad-EZH2 mut 3’-UTR vectors for the luciferase reporter assay. The data showed a marked decrease in the luciferase activity of Ad-EZH2 3’-UTR in the foam cells (*P* < 0.05). Also, no changes in the luciferase activity of Ad-EZH2 mut 3’-UTR were observed (Fig 5G). Based on these findings, we hypothesize that miR-92a directly interacted with the 3’-UTR of EZH2 in foam cells.

## Discussion

Posttranslational histone modifications play an important role in the transcriptional process. EZH2 is a key component of PRC2, which mediates tissue-specific gene silencing through trimethylation of H3K27 [16]. Several studies have documented the involvement of EZH2 in atherosclerosis [18]; however, the precise role of EZH2 in atherogenesis and the molecular mechanisms remain largely unknown. It has been recently reported that some miRNAs regulate EZH2 expression at the post-transcriptional level [20]. In this study, we showed for the first time that EZH2 and H3K27met3 levels were increased in the aortic tissue of ApoE-/- mice fed the methionine diet. Increased expression of EZH2 promotes lipid deposition *in vitro*, as supported by our results showing elevated levels of both TG and TC in the foam cells. Moreover, the results from the western blot analysis and 3’UTR of the EZH2 luciferase assay confirmed that miR-92a might suppress EZH2 expression by binding to the 3’UTR of EZH2 mRNA. These findings indicate that EZH2 may regulate the initiation and development of Hcy-induced atherosclerosis, and therefore serve as a novel target for gene therapy.

Atherosclerosis originates from a fatty streak within the intimal layer of the artery because of accumulating lipid-engorged foam cells. These foam cells are macrophages that have engulfed oxidized low-density lipoprotein cholesterol particles. A large body of evidence indicates that dyslipidemia might play a crucial role in vascular pathology associated with HHcy [18]. In this study, ApoE-/- mice receiving methionine diet displayed high levels of serum Hcy and typical atherosclerotic plaques in the thoracic aorta. Since Hcy is a branch-point intermediate in the methionine cycle, folate and vitamin B12 are essential for the conversion of Hcy during methionine metabolism [5]. Our data revealed that folate and B12 supplements decreased both the serum Hcy levels and the size of the plaques. *In vitro* experiments also showed that Hcy accelerated lipid accumulation in the foam cells. These results suggested that Hcy might promote the development of atherosclerosis as well as formation of the foam cells, which is in line with previously reported studies [4].

Epigenetic processes play an important role in Hcy metabolism, including histone modification and miRNA regulation. Histone methylation is one of the major types of epigenetic modification. However, limited evidence on the effect of HHcy in atherogenesis via histone methylation is available. Histones have multiple specific reversible post-translational modifications, especially on the N-terminal tail of histones enriched with lysine and arginine residues [22]. Based on the position of the histone chain, methylated lysine is related to transcriptional activation or suppression. The modification of histones by methylation of the lysine residues has been shown to regulate gene expression by affecting chromatin structure. Histone methyltransferase (HMTs) may catalyze the transfer of a methyl group from SAM to a lysine residue on H3 [23]. For instance, methylation state of H3K9 is a strong characteristic of transcriptional repression [24], but trimethylation state of H3K4 is correlated with gene activation [25]. The lysine residue modification of H3K27 might be in the mono-, di-, or tri-methylated states. Methylation of H3K27 is linked to the formation of tight heterochromatin chromatin and transcriptional silencing [21]. H3K27me1 is involved in the regulation of X-linked gene expression, and H3K27me2 is a repressive histone modification to suppress gene transcription [26]. In rats, diet-induced HHcy affects global protein arginine methylation and influences H3R8 methylation [27]. In addition, HHcy decreases H4R3me2 levels in the liver of cystathionine β-synthase deficient mice [6]. In this study, we showed that the levels of H3K27me3 were markedly increased in the aorta, and no differences were observed in the levels of H3K27me1 and H3K27me2. Moreover, Hcy-induced H3K27me3 hypermethylation was also observed in foam cells. Hcy can lead to epigenetic dysregulation of gene expression by affecting the histone-methylation enzymes. EZH2 methyltransferase silences gene expression through methylation of histone H3 on lysine 27 (H3K27) [16]. Loss of EZH2 moved the balance of cortical progenitors from self-renewal to differentiation in the brain [28]. Deletion of LXR (liver X receptor) results in aberrant cholesterol ester accumulation, which is linked to increased expression of EZH2 in mice fed a high-cholesterol diet [29]. We found increased mRNA and protein expression of EZH2 in both ApoE-/- mice and foam cells. To our surprise, overexpression of EZH2 in the foam cells resulted in elevated levels of TC and TG, whereas knockdown of EZH2 by RNA interference caused reduction in TC and TG levels. Our data provide evidence for the new function of EZH2 in affecting lipid metabolism. Moreover, we found a similar trend of H3K27me3 levels after up/down regulated EZH2 expression, which suggested that H3K27me3 might be a vital mechanism for EZH2 on dyslipidemia of foam cells.

Studies showed that miRNAs may affect transcriptional regulation of EZH2 via binding to the 3’UTR region of its mRNA [20]. These miRNAs were found to be widely related to blood lipids in the patients with cardiovascular diseases. Bioinformatics software TargetScan found that EZH2 was one of the potential targets of miR-92a. miR-92a is a member of miR-17-92 cluster located on chromosome 13q31.3, and is reduced profoundly in stable coronary artery disease. Liu and colleagues found that miR-92a was positively related to high-density lipoprotein cholesterol, but negatively related to lipoprotein-a. In our results, miR-92a levels were significantly decreased in ApoE-/- mice fed a high methionine diet as well as in Hcy treated foam cells, suggesting that miR-92a was involved in Hcy induced atherosclerosis. *In vitro* experiments showed that exogenous miR-92a affected EZH2 expression both at the transcriptional and translational levels. Moreover, luciferase reporter assay further proved that miR-92a directly interacted with the 3’-UTR of EZH2 in foam cells.

In summary, our data demonstrate elevated H3K27me3 modification in aortas with Hcy-induced atherosclerotic plaques. Increased levels of H3K27me3 were accompanied by alterations in the corresponding histone methyltransferase EZH2, which was regulated by miR-92a. Furthermore, inhibition of EZH2 resulted in reduced lipid accumulation. These findings help us understand the molecular mechanisms in Hcy-induced atherosclerosis, and also give us a strong rationale to further investigate miR-92a and EZH2 as potential diagnostic or therapeutic targets for atherosclerosis.
