# Correction: Hyperhomocysteinemia in ApoE-/- Mice Leads to Overexpression of Enhancer of Zeste Homolog 2 via miR-92a Regulation

**DOI:** 10.1371/journal.pone.0240762

**Published:** 2020-10-12

**Authors:** Yang Xiaoling, Zhao Li, Li ShuQiang, Ma Shengchao, Yang Anning, Ding Ning, Li Nan, Jia Yuexia, Yang Xiaoming, Li Guizhong, Jiang Yideng

Following publication of this article [1], concerns were raised regarding duplication of an image. Specifically, Fig 2 bottom middle panel, ADFP stain 100μM Hcy, in [1] is a duplicate of Fig 6D top middle panel, miR-148a mimic+Hcy, in a subsequent publication by some of the same authors [2].

The authors confirm that the image used in the *PLOS ONE* article [1] is correct, while the duplicate image in [2] is incorrect. The authors apologise for any confusion.

There is an error in the caption for Fig 3. Please see the complete correct caption here.

**Fig 3:**
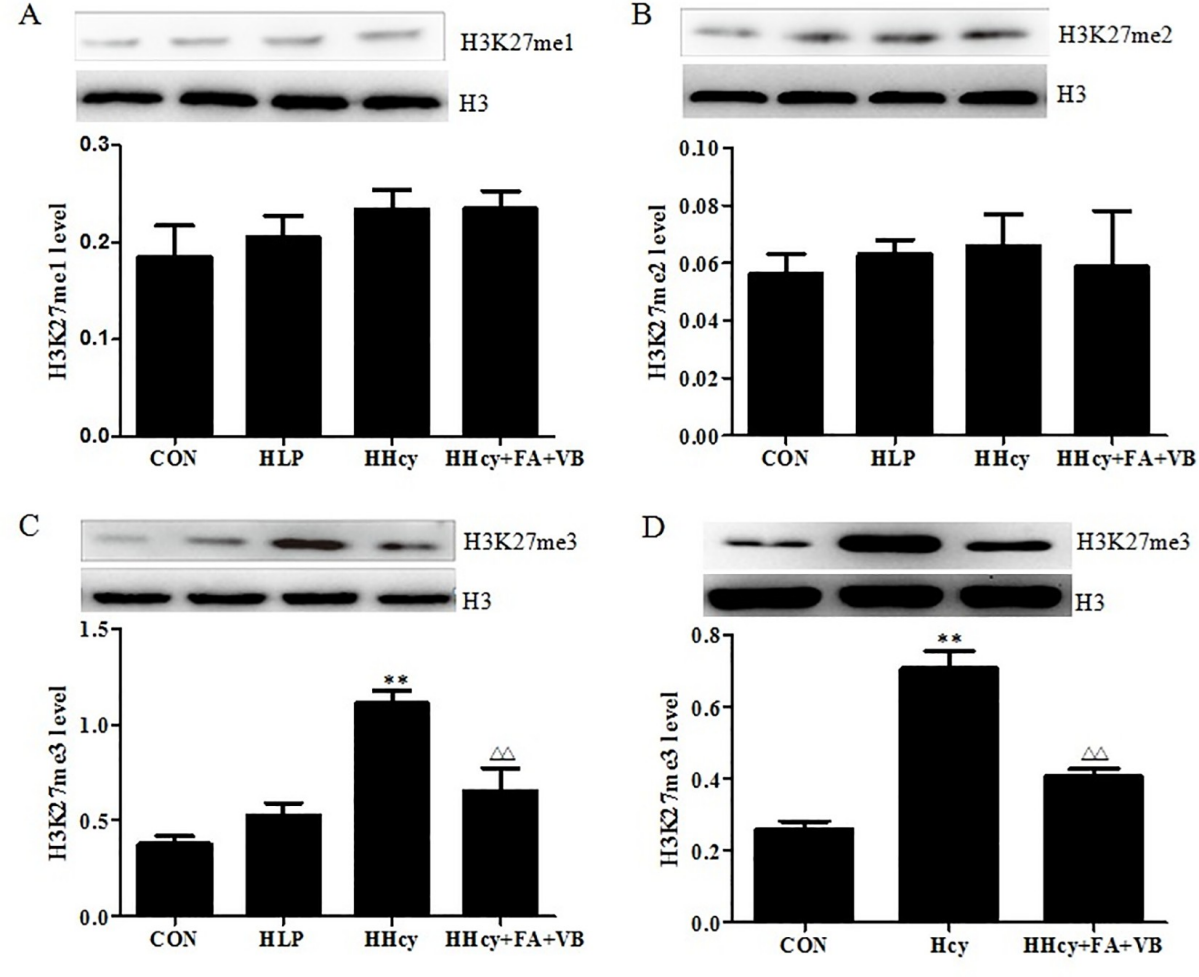
Hcy increased H3K27me3 level. (A, B, C) H3K27me1, 2, 3 levels in the aortic tissue of the mice were detected by Western blot. (D) H3K27me3 level was detected in macrophage foam cells by Western blot. Signal intensity of H3K27me1, 2, 3 was quantified by densitometric analysis and normalized to pan H3 control. Data were presented as mean ± SD. *P < 0.05, **P < 0.01, vs. CON. ##*P*< 0.01, vs. HLP. Δ*P* < 0.05, ΔΔ*P* < 0.01, vs. HHcy or 100 μM Hcy. CON: WT C57BL/6J mice were fed with a regular mouse diet; HLP: ApoE-/-mice were fed standard mouse diet; HHcy: ApoE-/- mice were fed standard mouse diet plus methionine; HHcy+FA+VB: mice were fed standard mouse diet with methionine, folate and vitamin B12 supplements.

There are errors in the figure mentions cited in the Hcy increase the H3K27me3 level subsection of the Results. Please see the correct text here:

Several studies have demonstrated that histone modification plays a vital role in atherosclerosis [18]. Hcy, a risk factor for atherosclerosis, is known to aggravate the progression of atherosclerosis via histone modification [5]. Thus, in order to explore the possible epigenetic mechanisms for Hcy-related pathogenesis of atherosclerosis, we assessed the levels of H3K27me1, 2, and 3 in both the aortic tissue and the foam cells. As shown in Fig 3, no differences were observed in the levels of H3K27me1 and 2 amongst different mice groups (Fig 3A and 3B). Interestingly, the levels of H3K27me3 in the HHcy group were 194% (*P* < 0.05) and 110% (*P* < 0.05) higher than the control and HLP groups, respectively (Fig 3C). Treatment with folate and vitamin B12 significantly decreased the level of H3K27me3. Consistent with the data from the *in vivo* experiments, the levels of H3K27me3 level increased to 263% compared to the control group, when the foam cells were treated with 100 μM Hcy for 48 h. However, the H3K27me3 level in the Hcy+FA+VB group decreased significantly to 71% (*P* < 0.05) relative to the Hcy group (Fig 3D). These results indicated that H3K27me3 was involved in the progression of atherosclerosis.

The following clarifications are provided by the authors for the western blot methodology: protein concentration was detected through BCA, and 20 μg of protein extracts were loaded onto 10% SDS-PAGE for electrophoresis. According to the protein ladder (#26616, Thermo Scientific), the gel containing the target protein was trimmed into a strip as follows: for H3K27me1,2,3 between the 15 and 25 kDa markers; for H3 between the 15 and 25 kDa markers; for EZH2 between the 70 and 100 kDa markers; and for β-Actin between the 35 and 55 kDa markers. Then the protein in the gel strip was transferred onto a PVDF-membrane. Experimental and control data for each western blot experiment were obtained using blots derived from independent parallel gels on which equal amounts of the same protein samples were loaded. Signal intensity for the target proteins was quantified by densitometric analysis and normalised to control proteins in each experiment. Three experimental replicates were included in each blot experiment. Western blot densitometry data are included in S1 File.

Here the authors provide in Supporting Information S1 File the underlying dataset, including the individual-level data points underlying the charts in Figs 3-5, and the underlying image files for Fig 2. Image files underlying the other figures are available upon request from the authors.

## Supporting information

S1 File Underlying data. (ZIP)
