# Supplementary figures and images for: Correction: Hyperhomocysteinemia in ApoE-/- Mice Leads to Overexpression of Enhancer of Zeste Homolog 2 via miR-92a Regulation
Source: PLoS One. 2020 Oct 12;15(10):e0240762. doi: 10.1371/journal.pone.0240762 (PMC7549832; doi:10.1371/journal.pone.0240762)

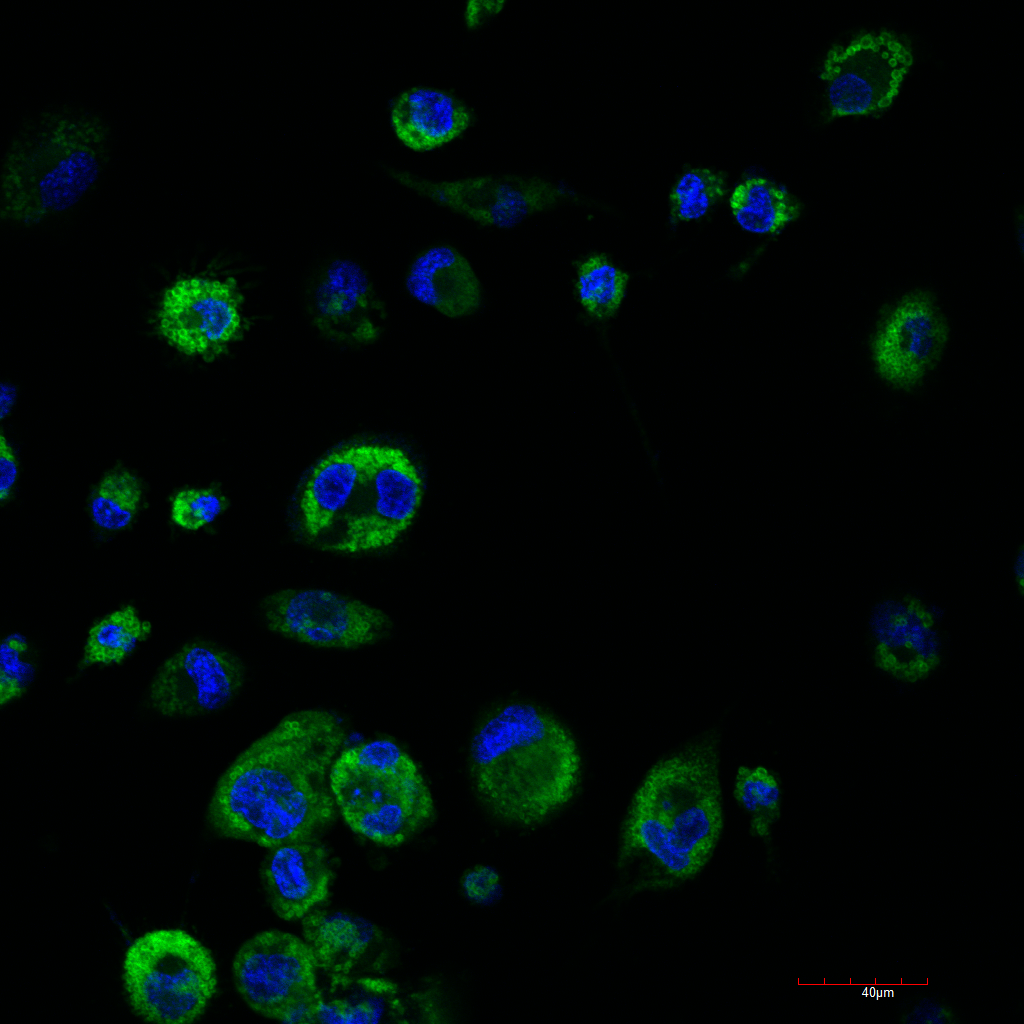

Supplement: S1 File — (ZIP) [file pone.0240762.s001.zip › SI Files Oct 2019/Fig2/ADFR stain/control/1/1--0.tif]

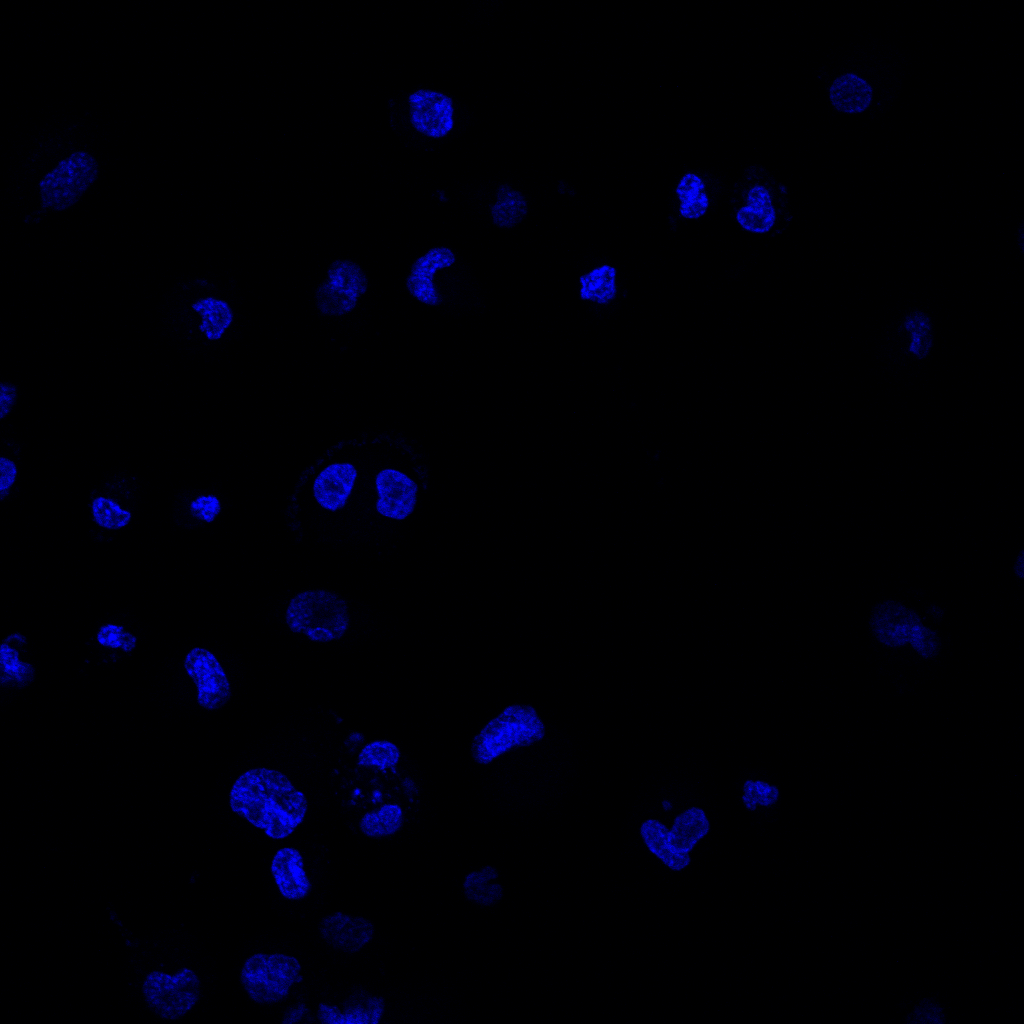

Supplement: S1 File — (ZIP) [file pone.0240762.s001.zip › SI Files Oct 2019/Fig2/ADFR stain/control/1/1--1.tif]

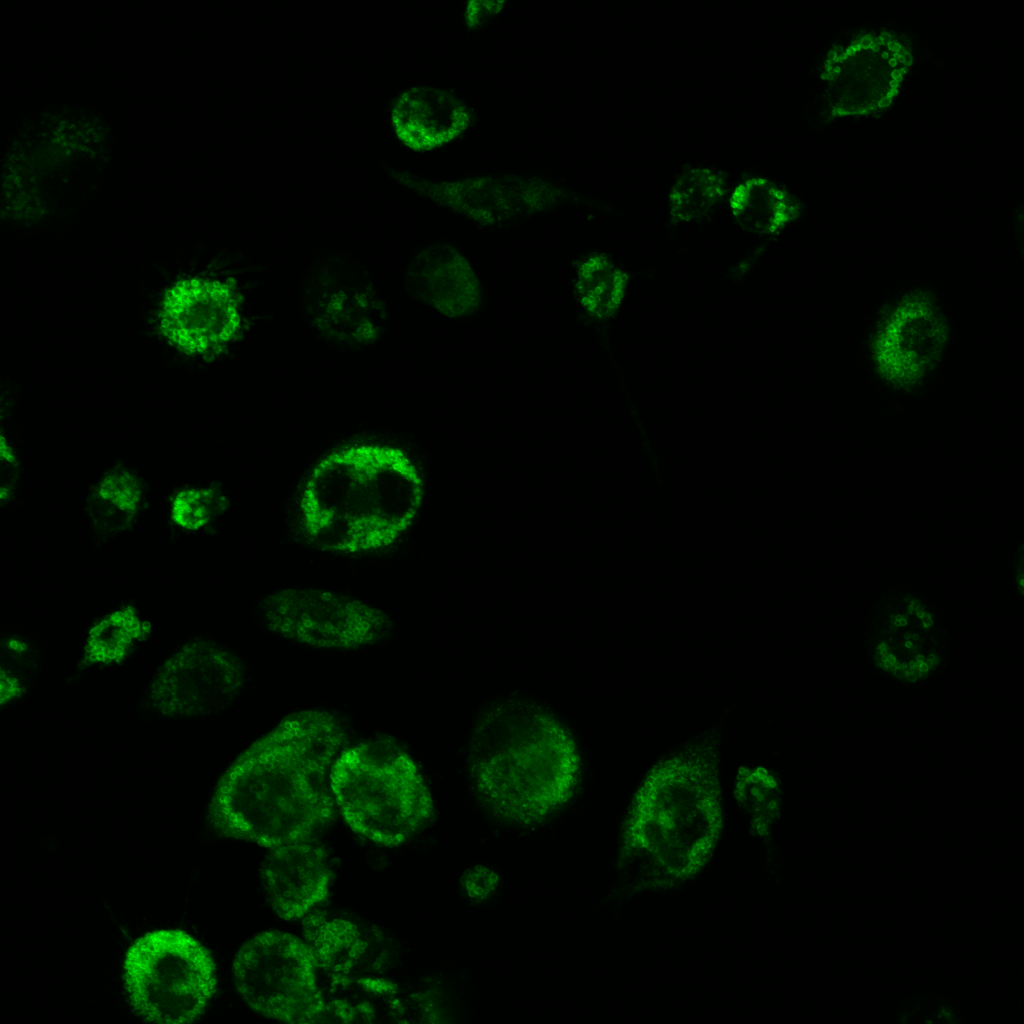

Supplement: S1 File — (ZIP) [file pone.0240762.s001.zip › SI Files Oct 2019/Fig2/ADFR stain/control/1/1--2.tif]

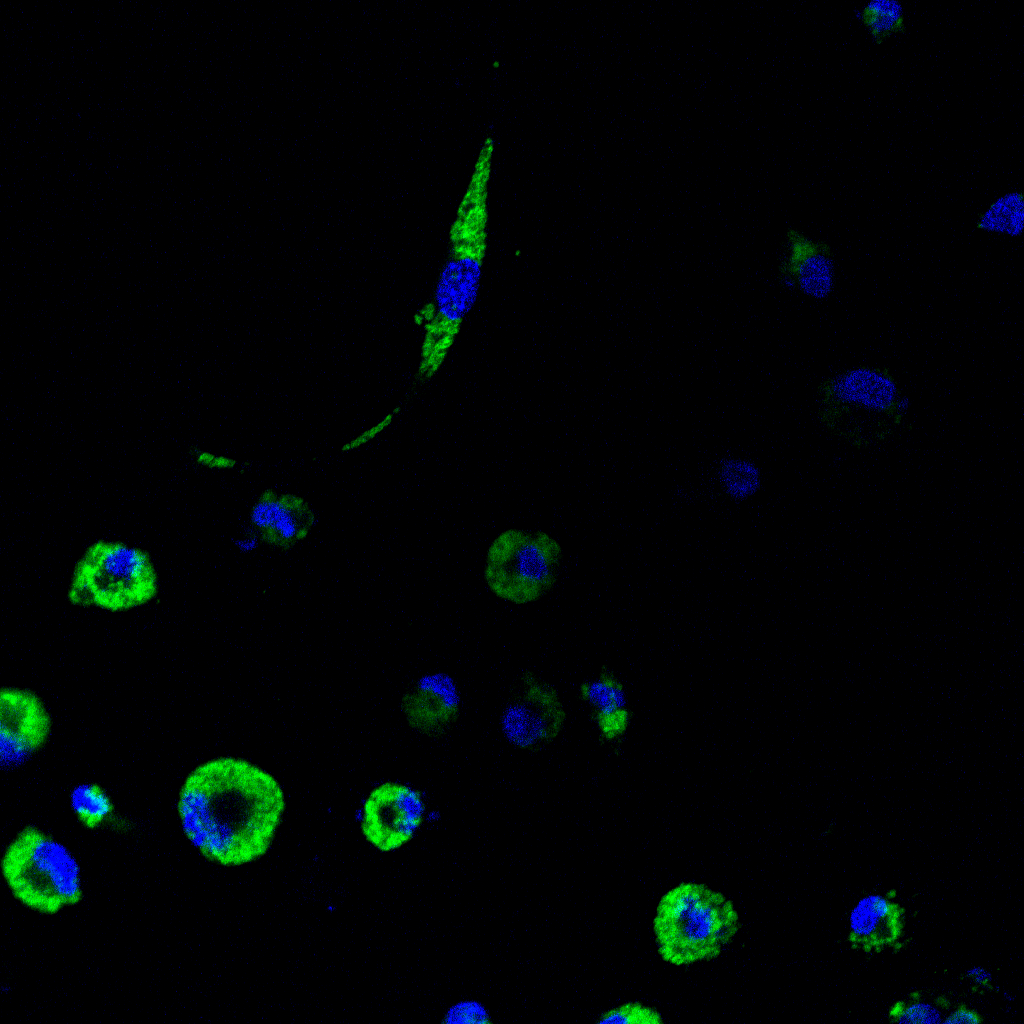

Supplement: S1 File — (ZIP) [file pone.0240762.s001.zip › SI Files Oct 2019/Fig2/ADFR stain/control/2/2--0.tif]

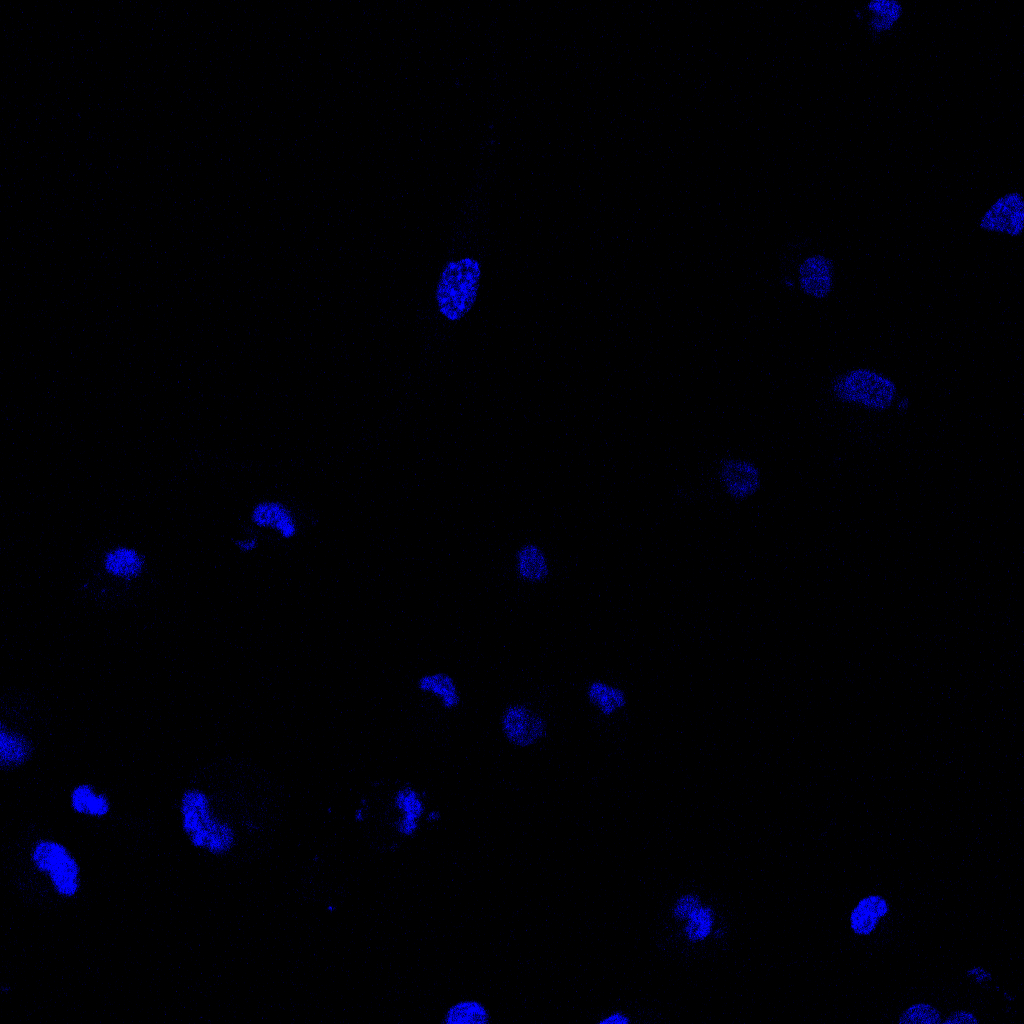

Supplement: S1 File — (ZIP) [file pone.0240762.s001.zip › SI Files Oct 2019/Fig2/ADFR stain/control/2/2--1.tif]

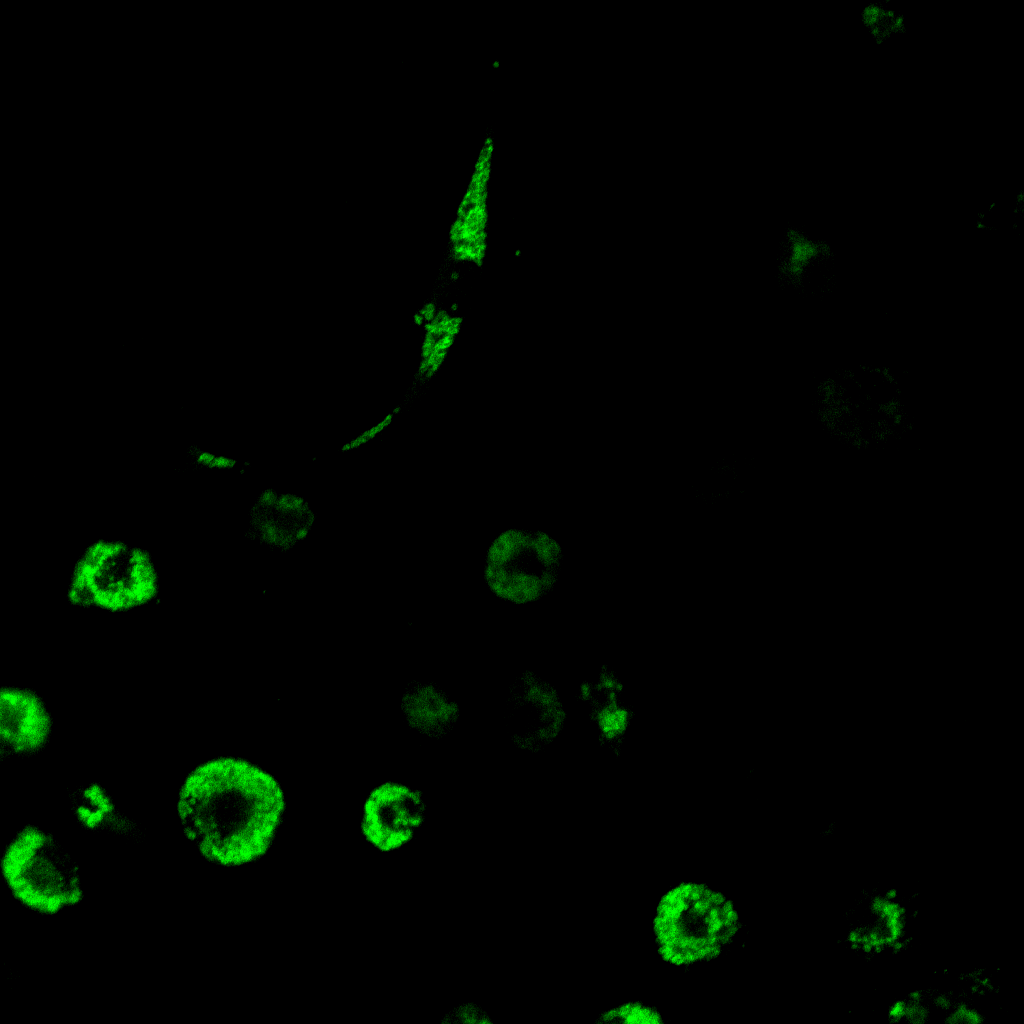

Supplement: S1 File — (ZIP) [file pone.0240762.s001.zip › SI Files Oct 2019/Fig2/ADFR stain/control/2/2--2.tif]

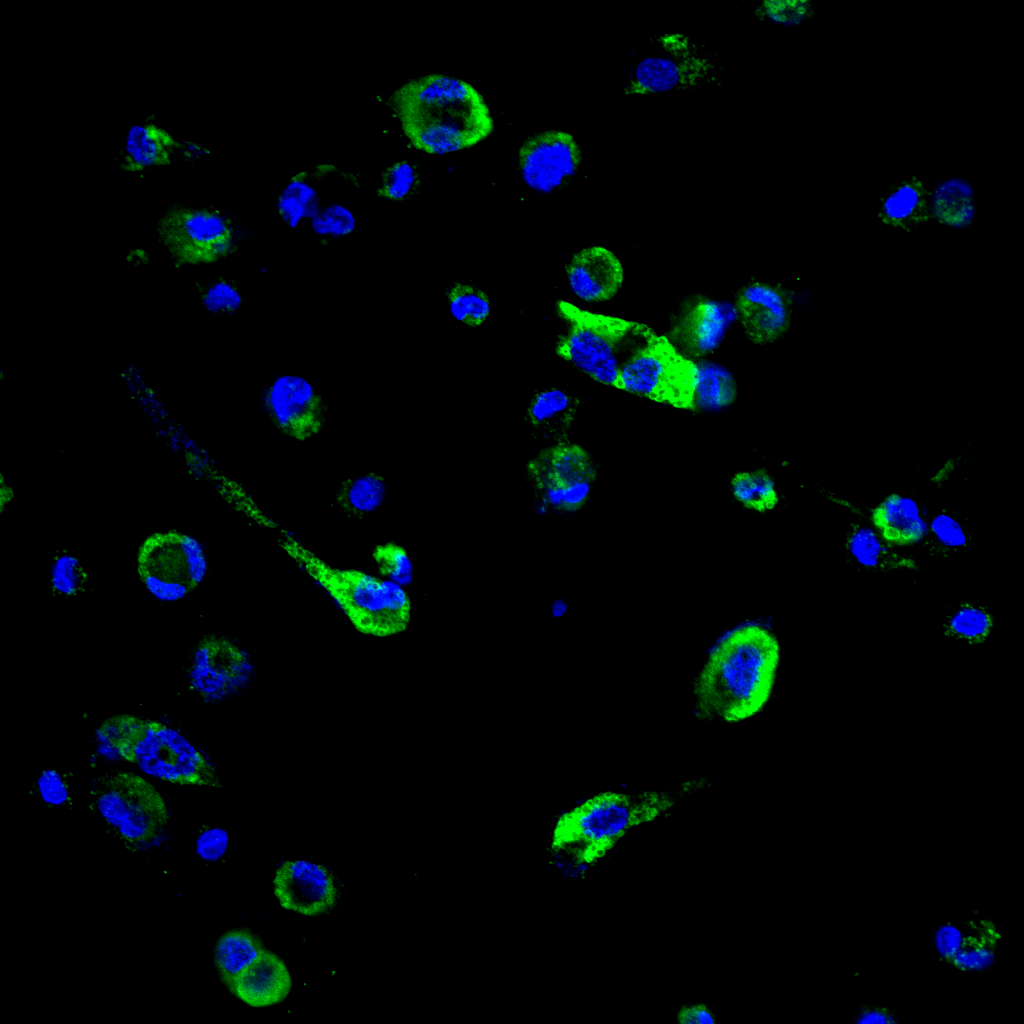

Supplement: S1 File — (ZIP) [file pone.0240762.s001.zip › SI Files Oct 2019/Fig2/ADFR stain/control/3/3--0.tif]

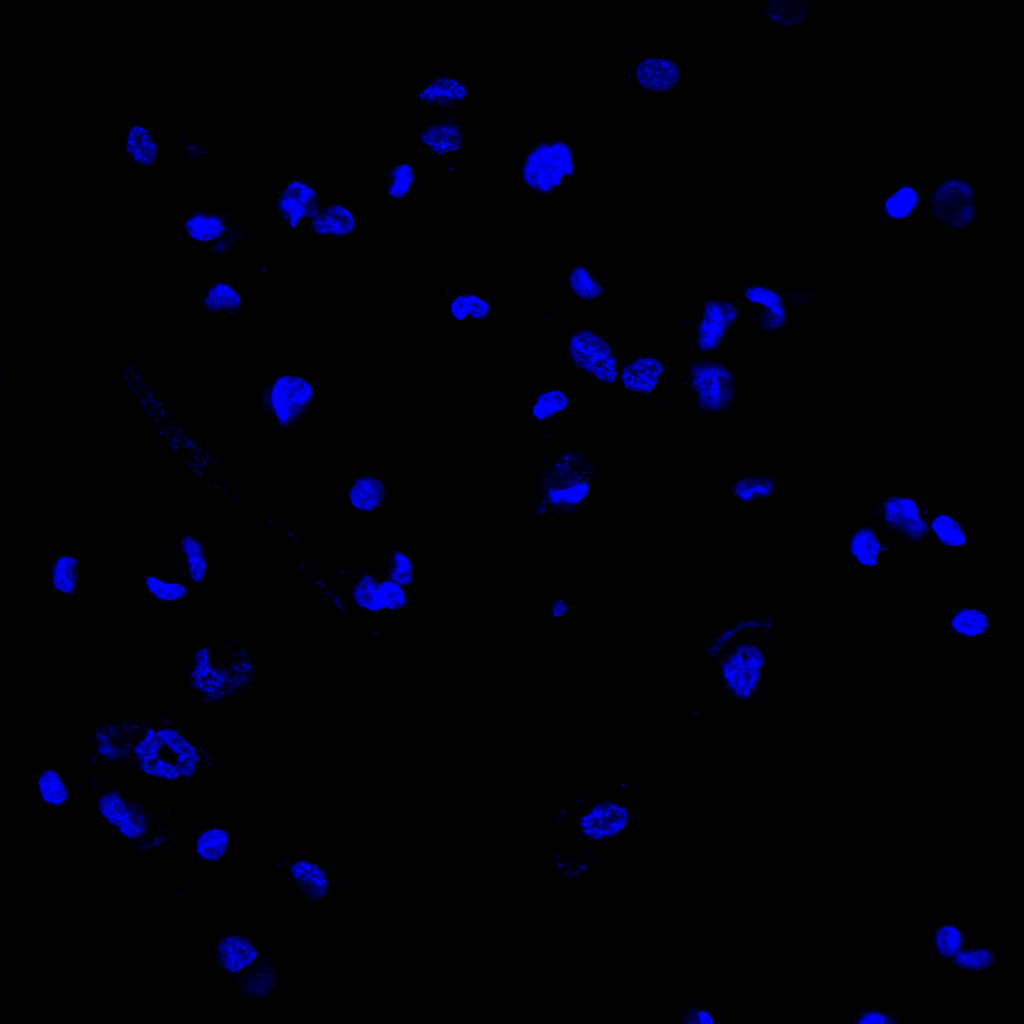

Supplement: S1 File — (ZIP) [file pone.0240762.s001.zip › SI Files Oct 2019/Fig2/ADFR stain/control/3/3--1.tif]

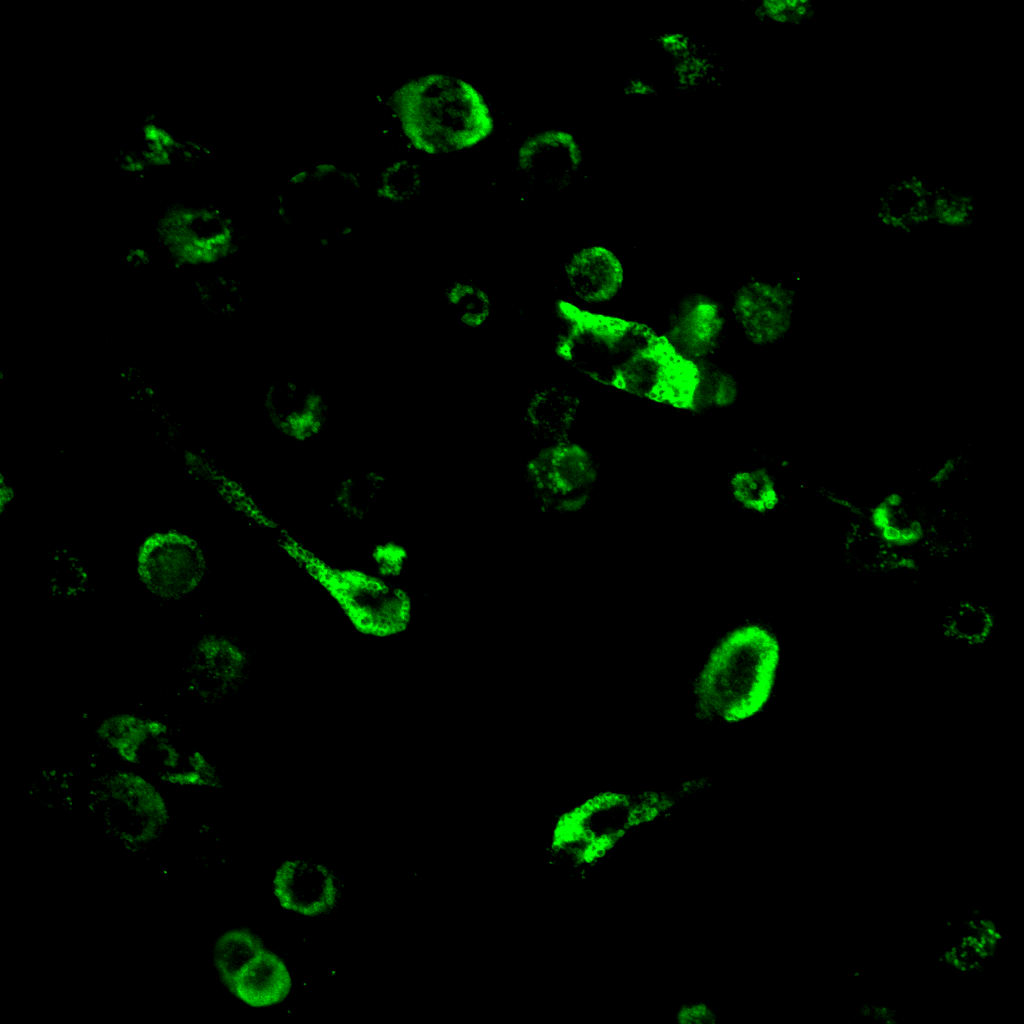

Supplement: S1 File — (ZIP) [file pone.0240762.s001.zip › SI Files Oct 2019/Fig2/ADFR stain/control/3/3--2.tif]

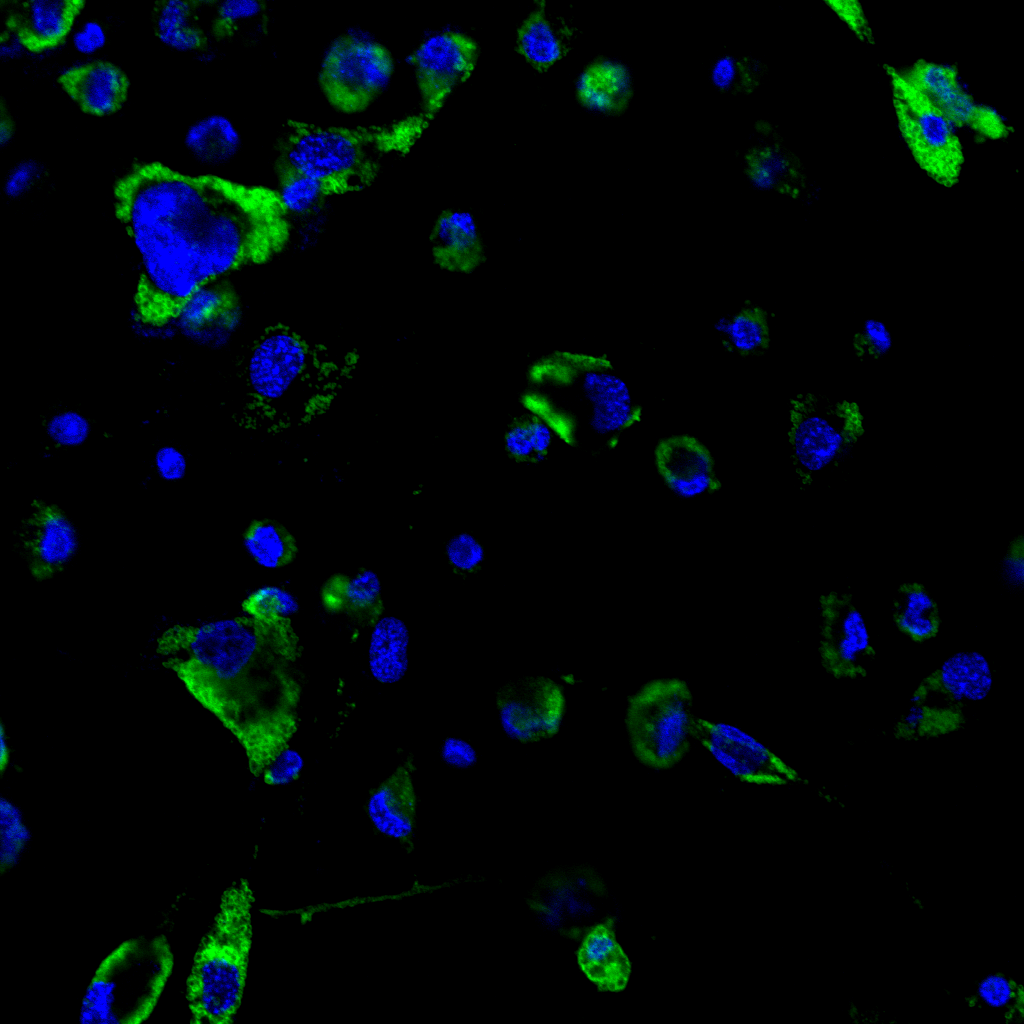

Supplement: S1 File — (ZIP) [file pone.0240762.s001.zip › SI Files Oct 2019/Fig2/ADFR stain/control/4/4--0.tif]

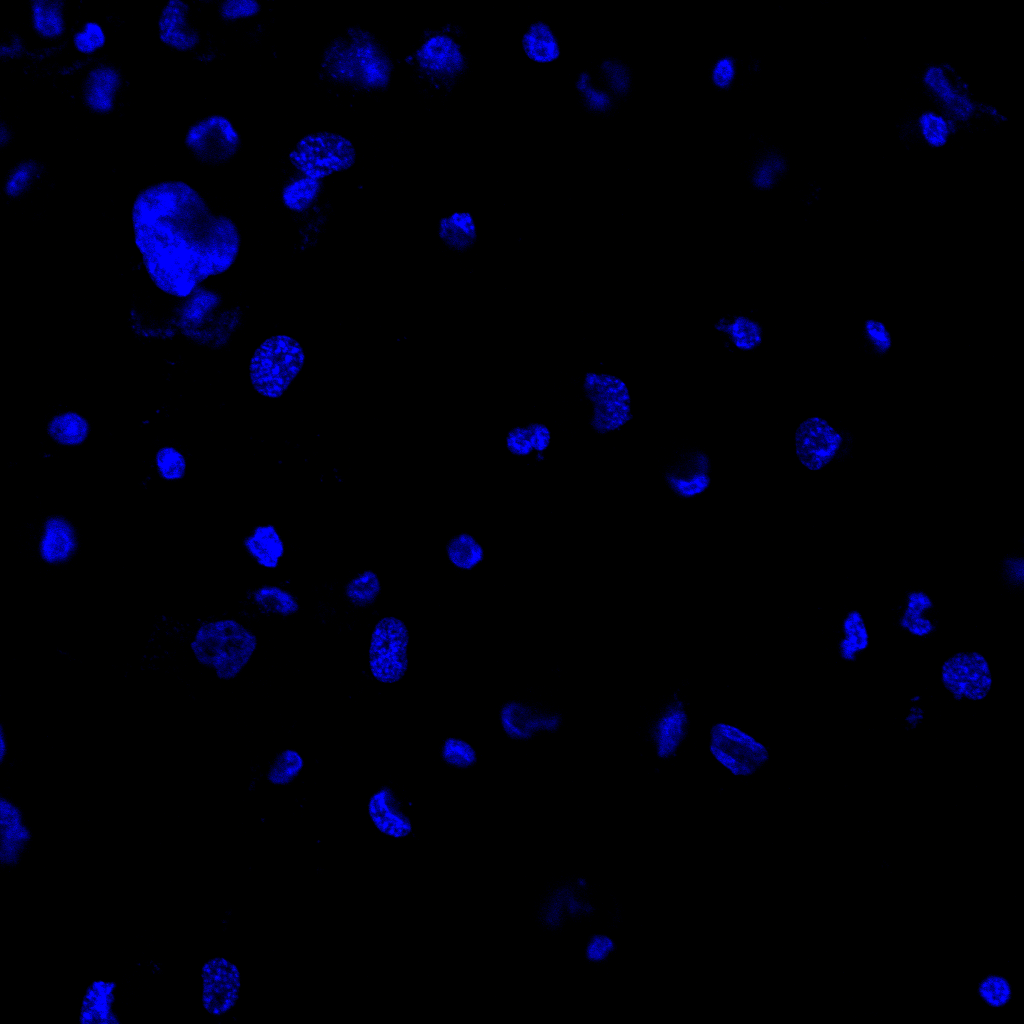

Supplement: S1 File — (ZIP) [file pone.0240762.s001.zip › SI Files Oct 2019/Fig2/ADFR stain/control/4/4--1.tif]

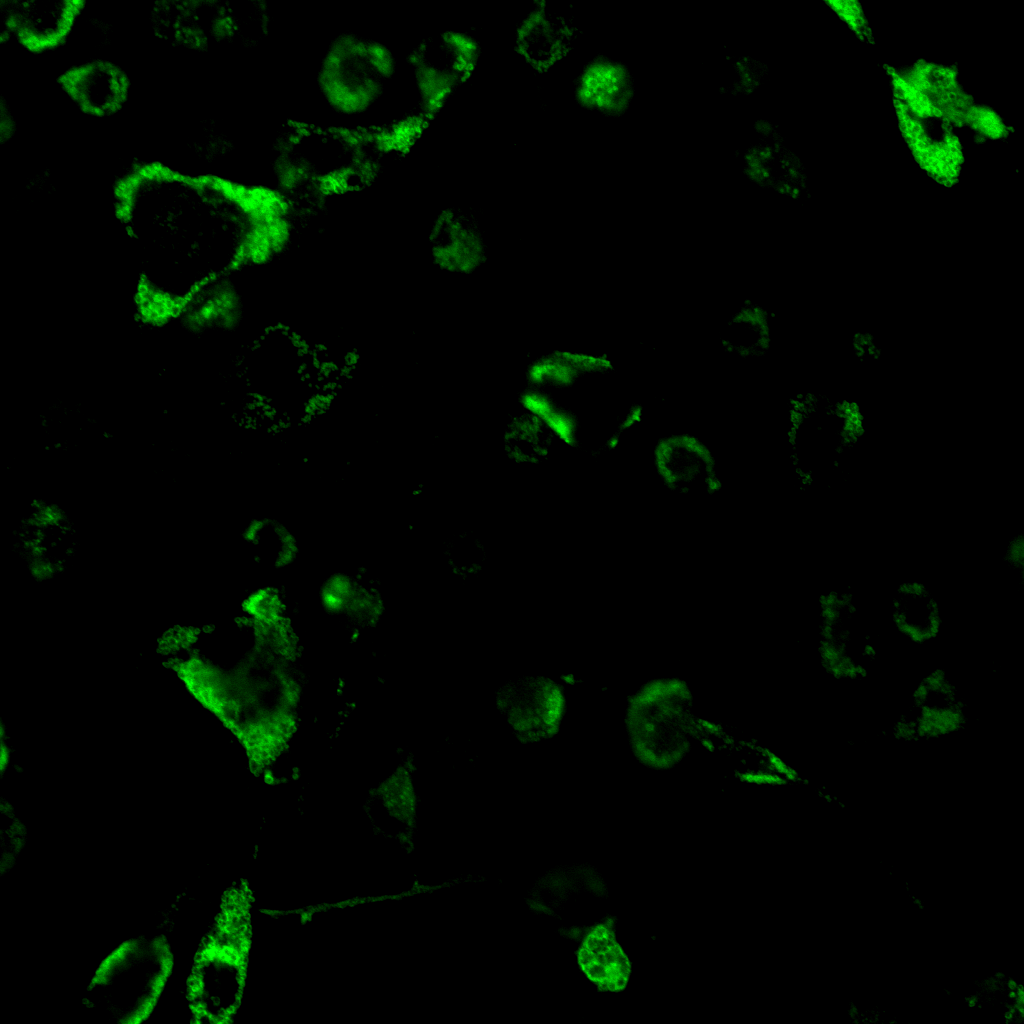

Supplement: S1 File — (ZIP) [file pone.0240762.s001.zip › SI Files Oct 2019/Fig2/ADFR stain/control/4/4--2.tif]

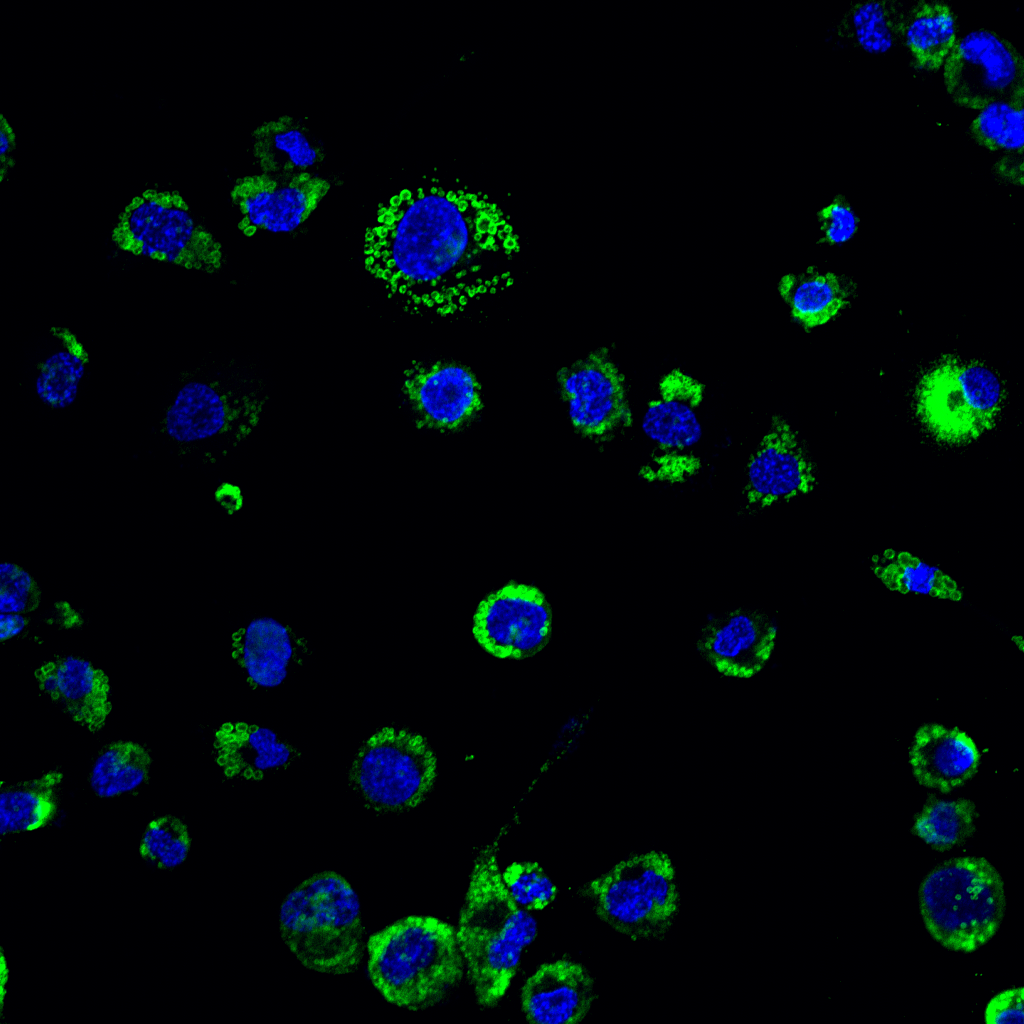

Supplement: S1 File — (ZIP) [file pone.0240762.s001.zip › SI Files Oct 2019/Fig2/ADFR stain/control/5/5--0.tif]

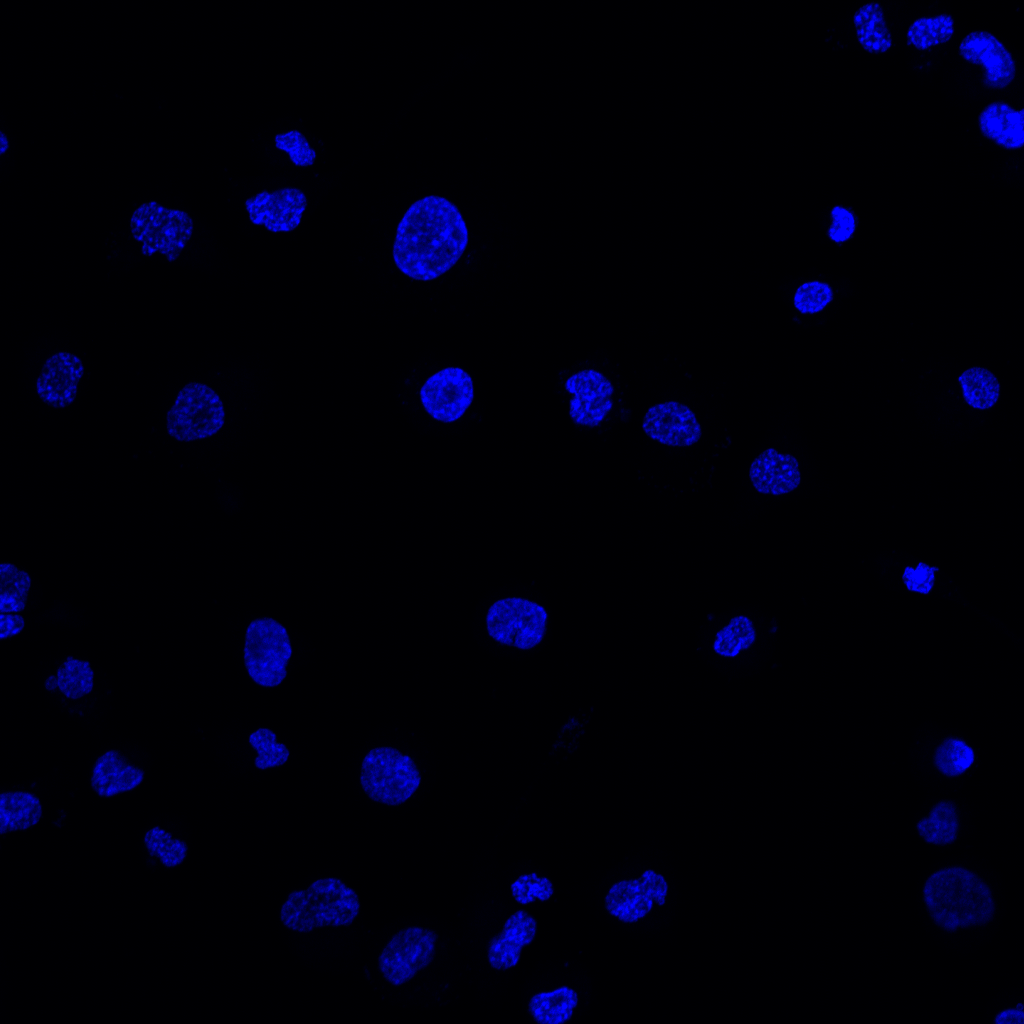

Supplement: S1 File — (ZIP) [file pone.0240762.s001.zip › SI Files Oct 2019/Fig2/ADFR stain/control/5/5--1.tif]

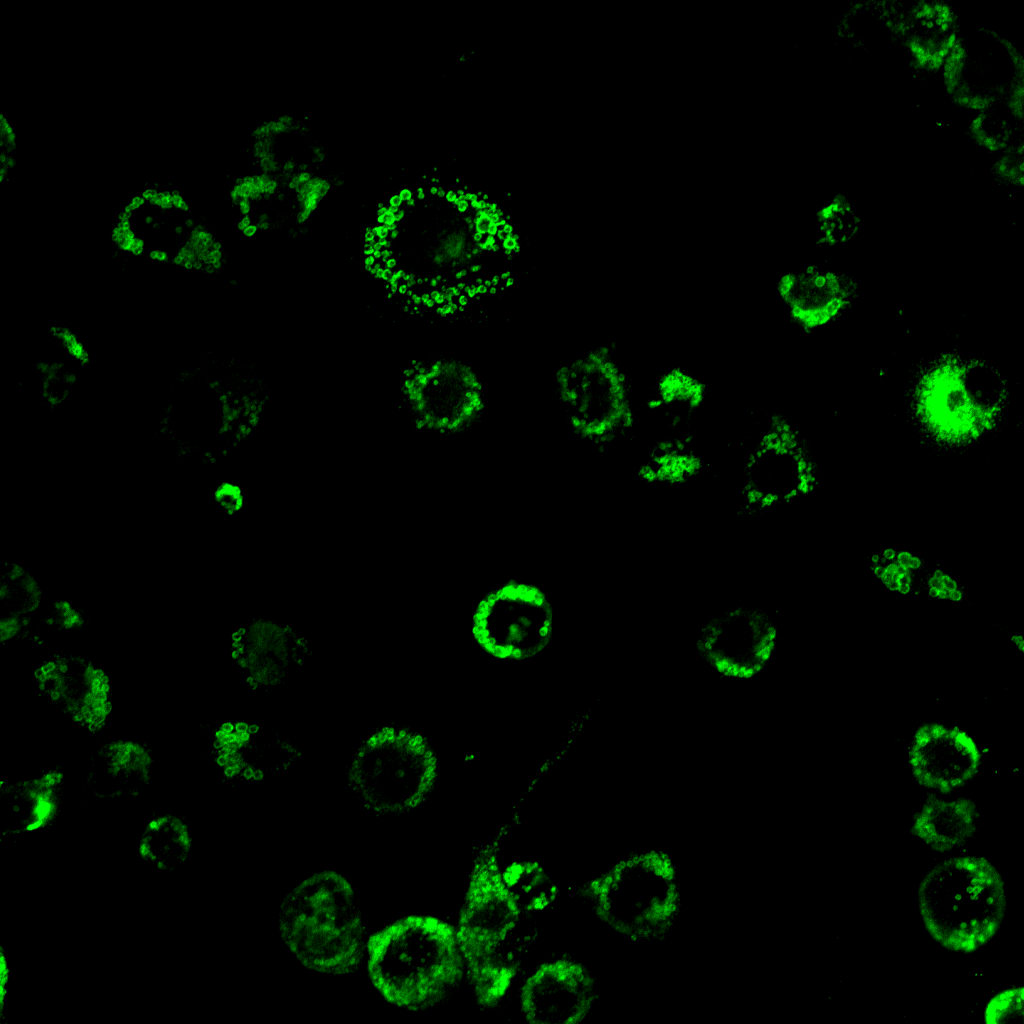

Supplement: S1 File — (ZIP) [file pone.0240762.s001.zip › SI Files Oct 2019/Fig2/ADFR stain/control/5/5--2.tif]

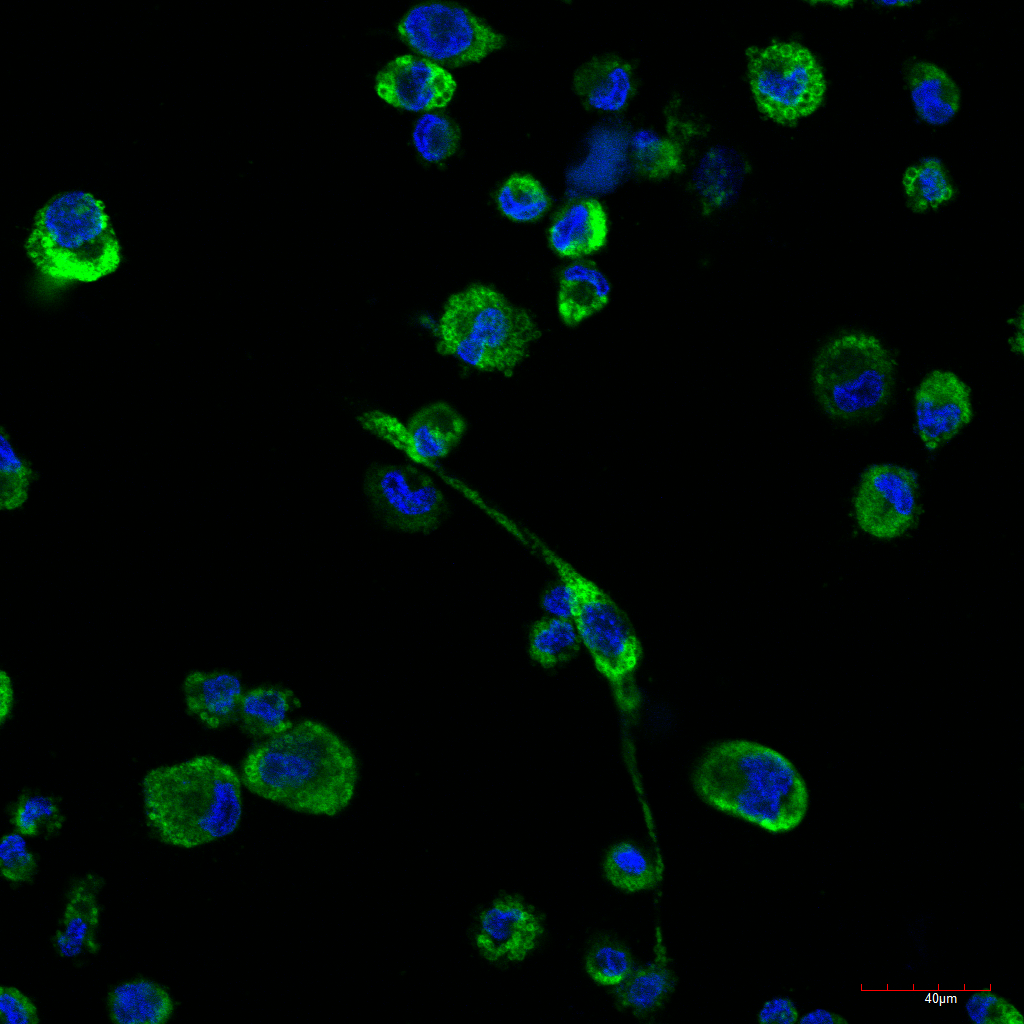

Supplement: S1 File — (ZIP) [file pone.0240762.s001.zip › SI Files Oct 2019/Fig2/ADFR stain/control/6/6--0.tif]

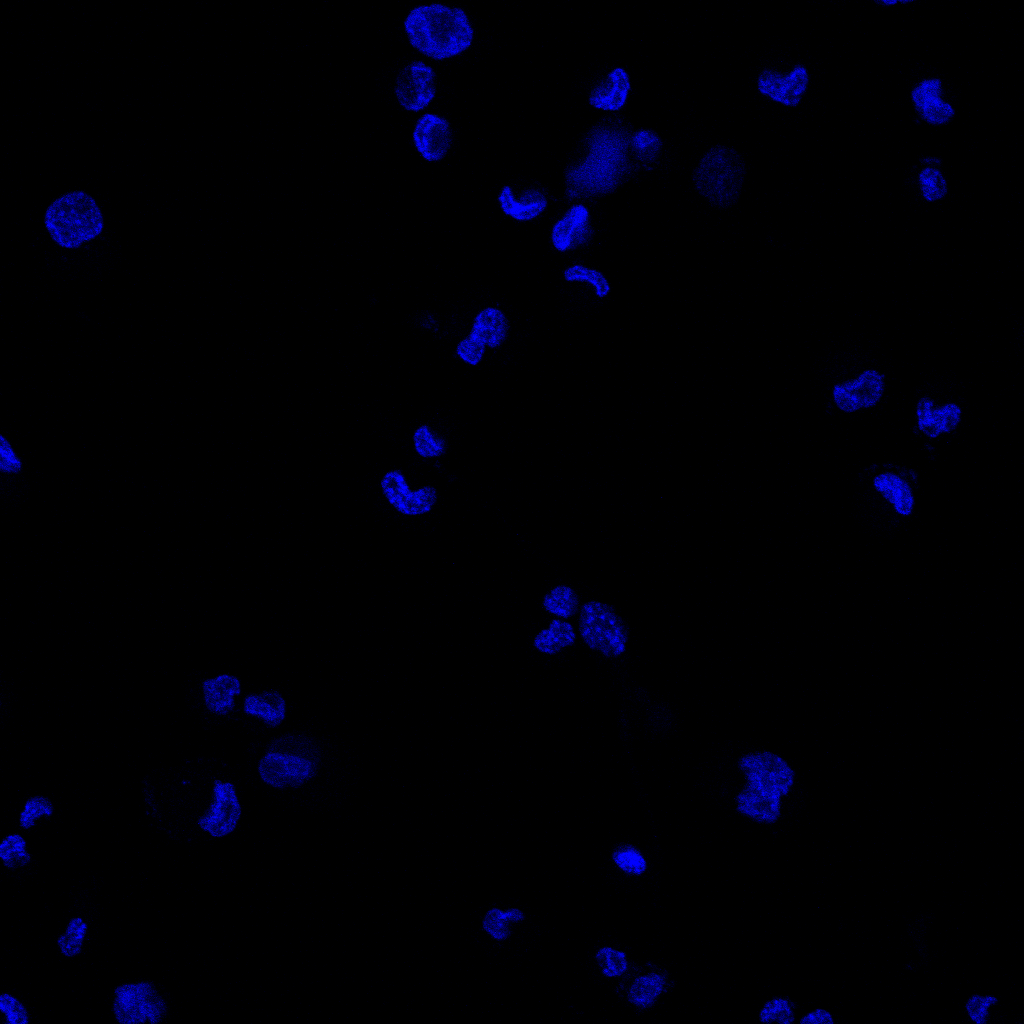

Supplement: S1 File — (ZIP) [file pone.0240762.s001.zip › SI Files Oct 2019/Fig2/ADFR stain/control/6/6--1.tif]

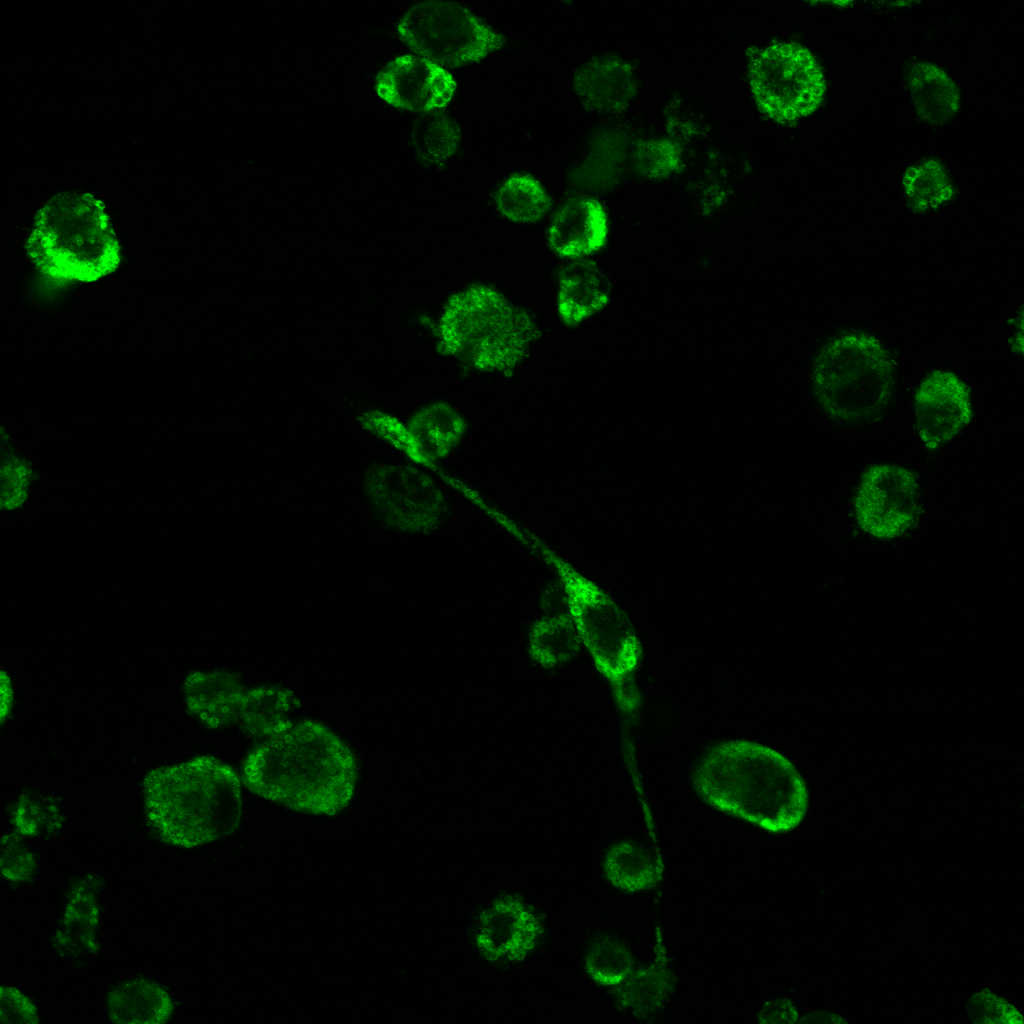

Supplement: S1 File — (ZIP) [file pone.0240762.s001.zip › SI Files Oct 2019/Fig2/ADFR stain/control/6/6--2.tif]

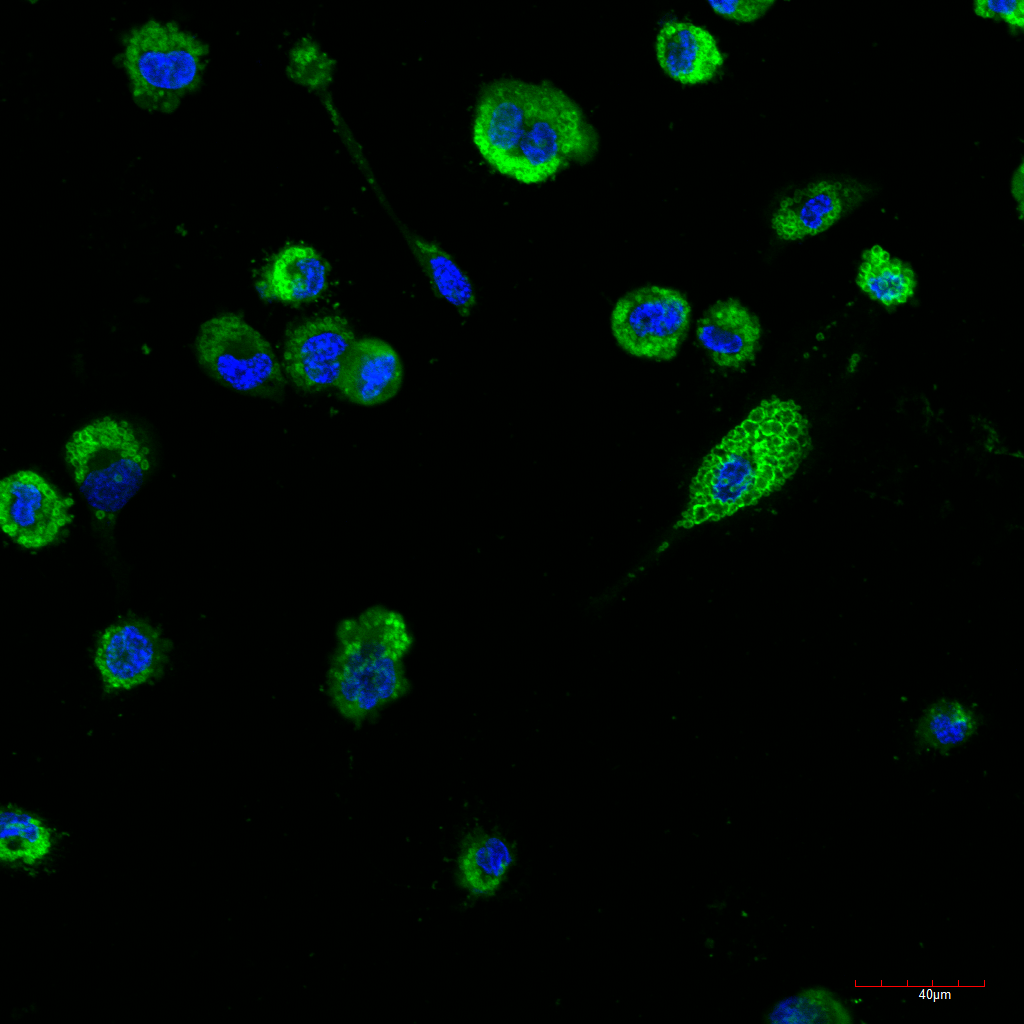

Supplement: S1 File — (ZIP) [file pone.0240762.s001.zip › SI Files Oct 2019/Fig2/ADFR stain/Hcy+folate/1/1--0.tif]

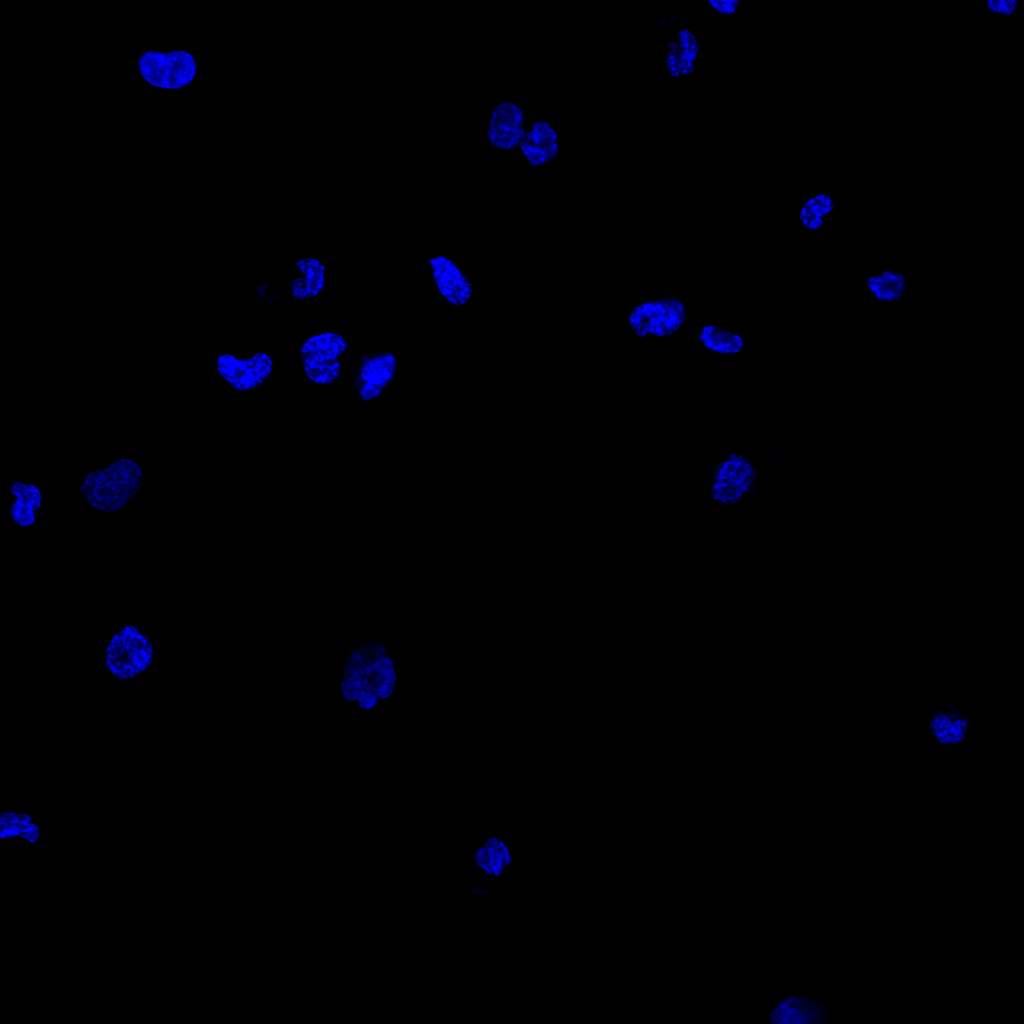

Supplement: S1 File — (ZIP) [file pone.0240762.s001.zip › SI Files Oct 2019/Fig2/ADFR stain/Hcy+folate/1/1--1.tif]

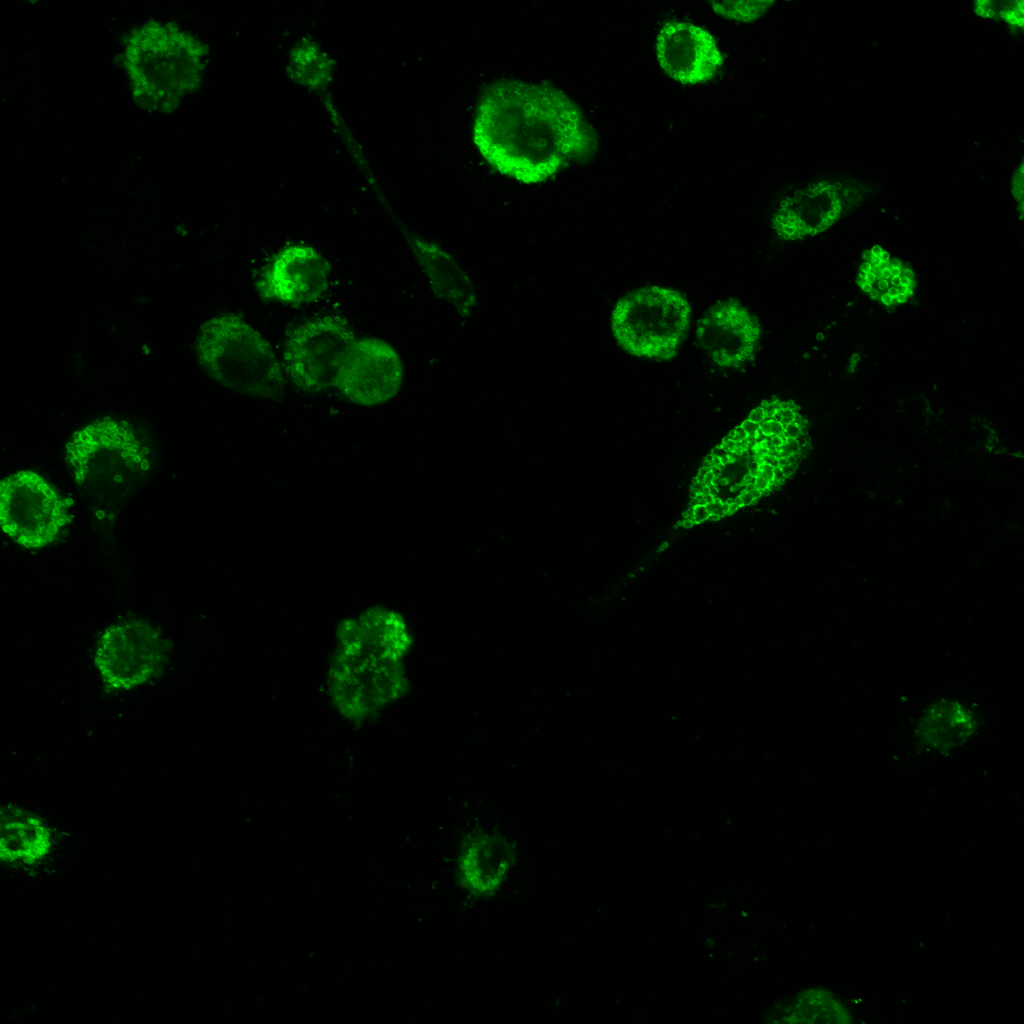

Supplement: S1 File — (ZIP) [file pone.0240762.s001.zip › SI Files Oct 2019/Fig2/ADFR stain/Hcy+folate/1/1--2.tif]

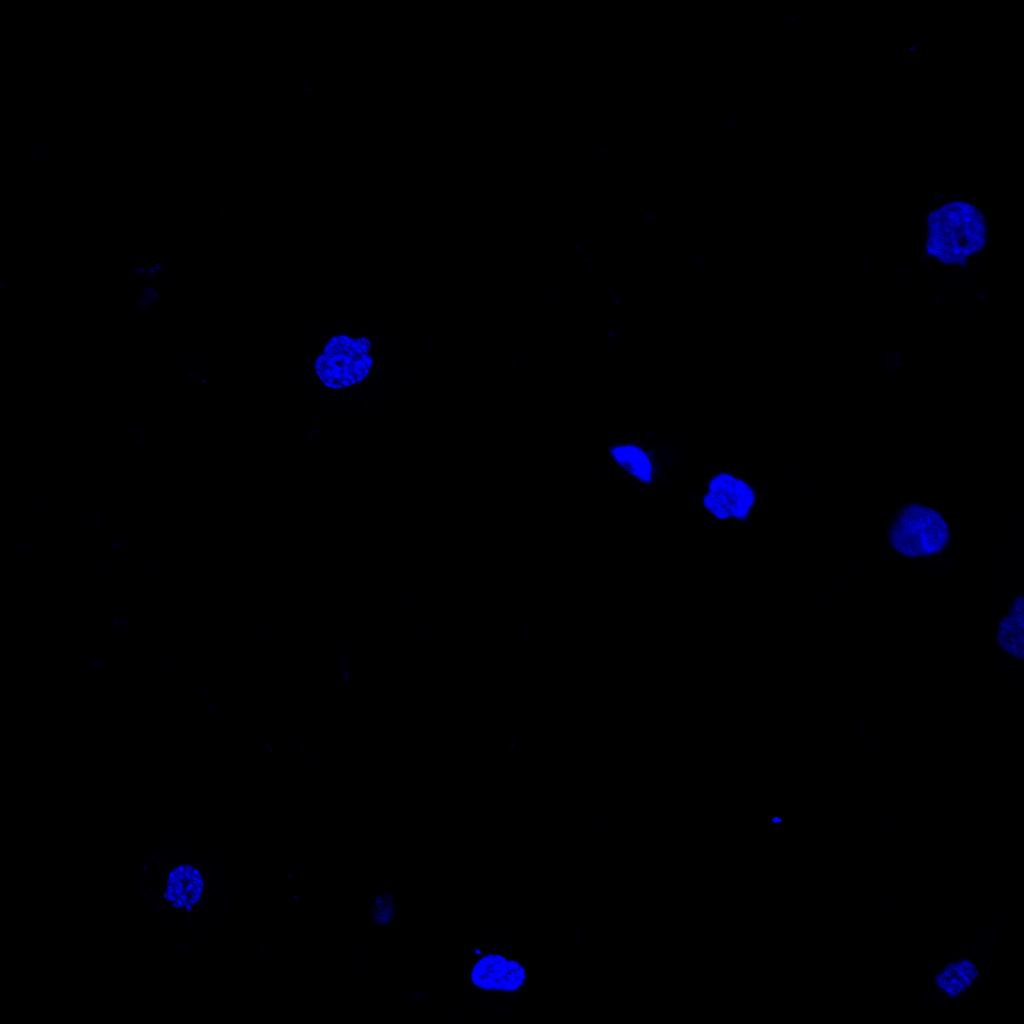

Supplement: S1 File — (ZIP) [file pone.0240762.s001.zip › SI Files Oct 2019/Fig2/ADFR stain/Hcy+folate/2/2--1.tif]

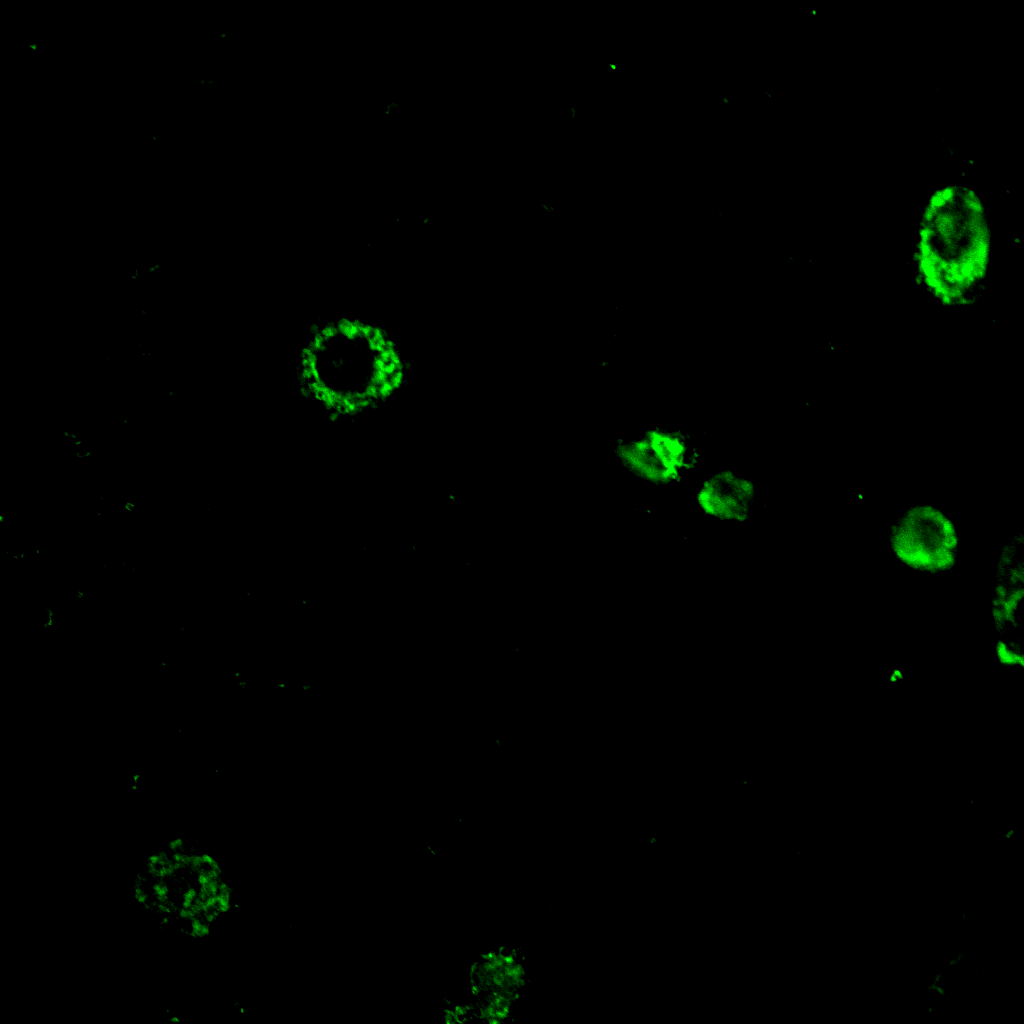

Supplement: S1 File — (ZIP) [file pone.0240762.s001.zip › SI Files Oct 2019/Fig2/ADFR stain/Hcy+folate/2/2--2.tif]

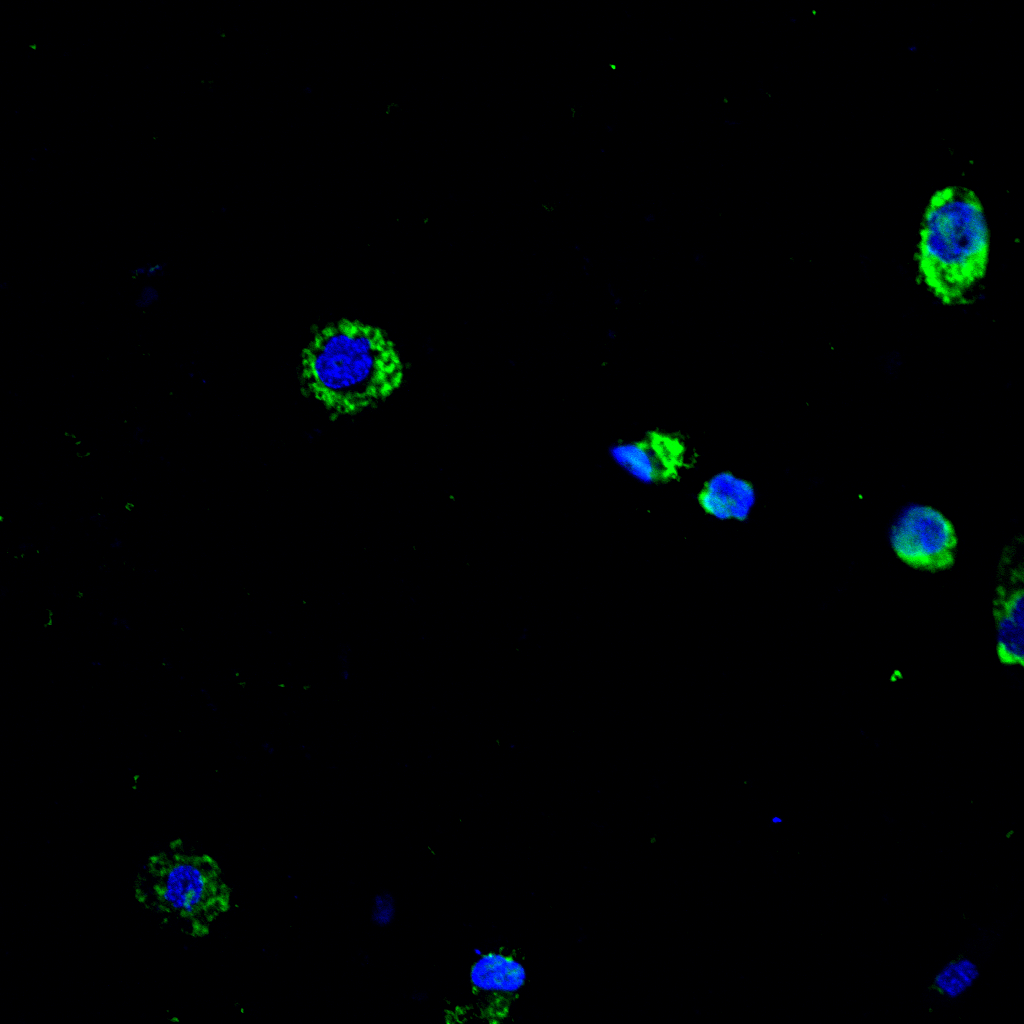

Supplement: S1 File — (ZIP) [file pone.0240762.s001.zip › SI Files Oct 2019/Fig2/ADFR stain/Hcy+folate/2/2-0.tif]

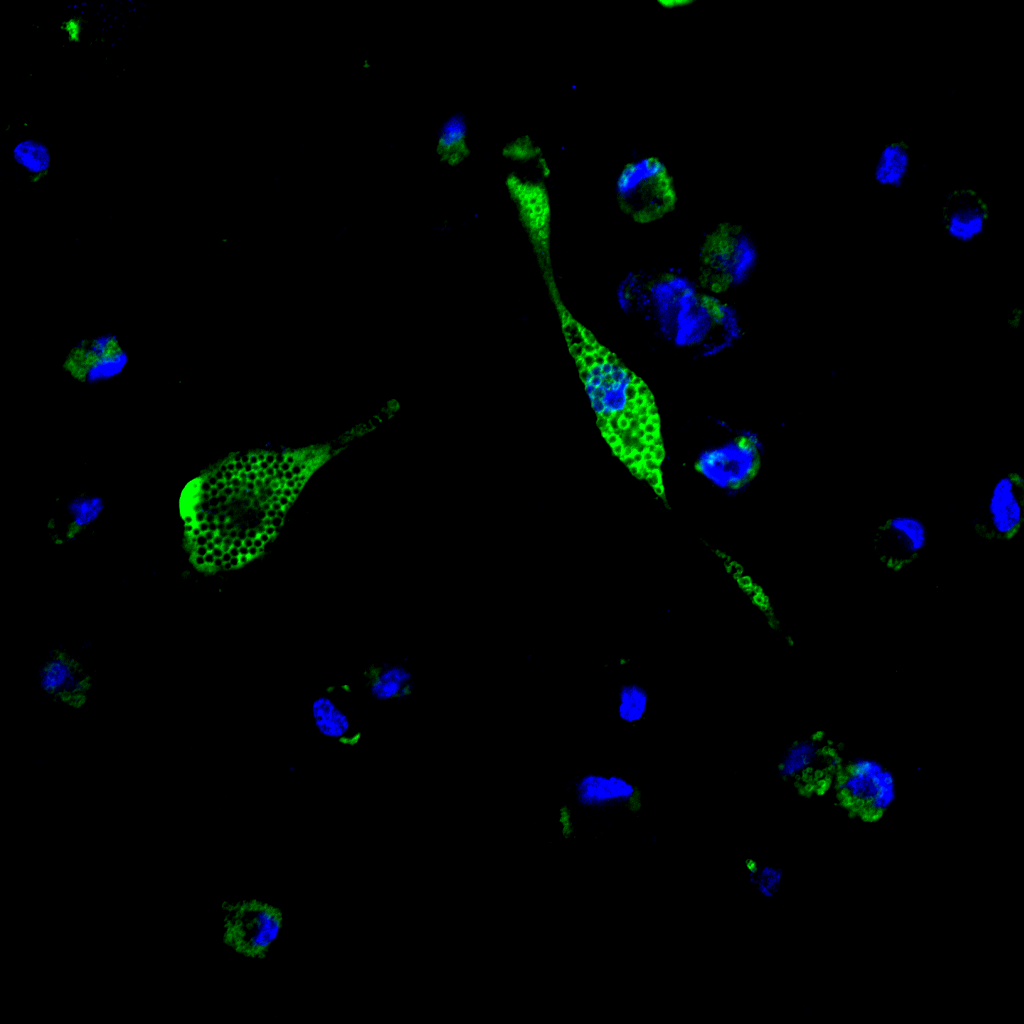

Supplement: S1 File — (ZIP) [file pone.0240762.s001.zip › SI Files Oct 2019/Fig2/ADFR stain/Hcy+folate/3/3--0.tif]

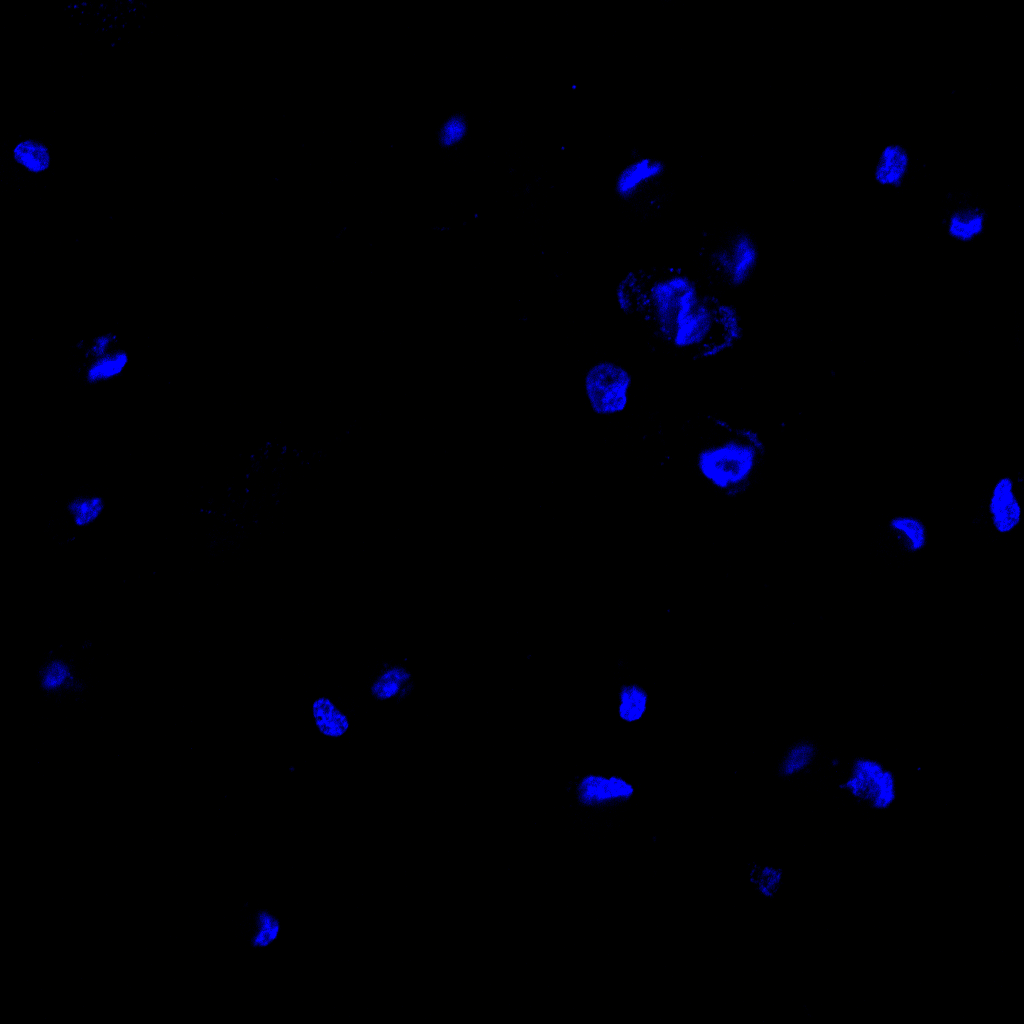

Supplement: S1 File — (ZIP) [file pone.0240762.s001.zip › SI Files Oct 2019/Fig2/ADFR stain/Hcy+folate/3/3--1.tif]

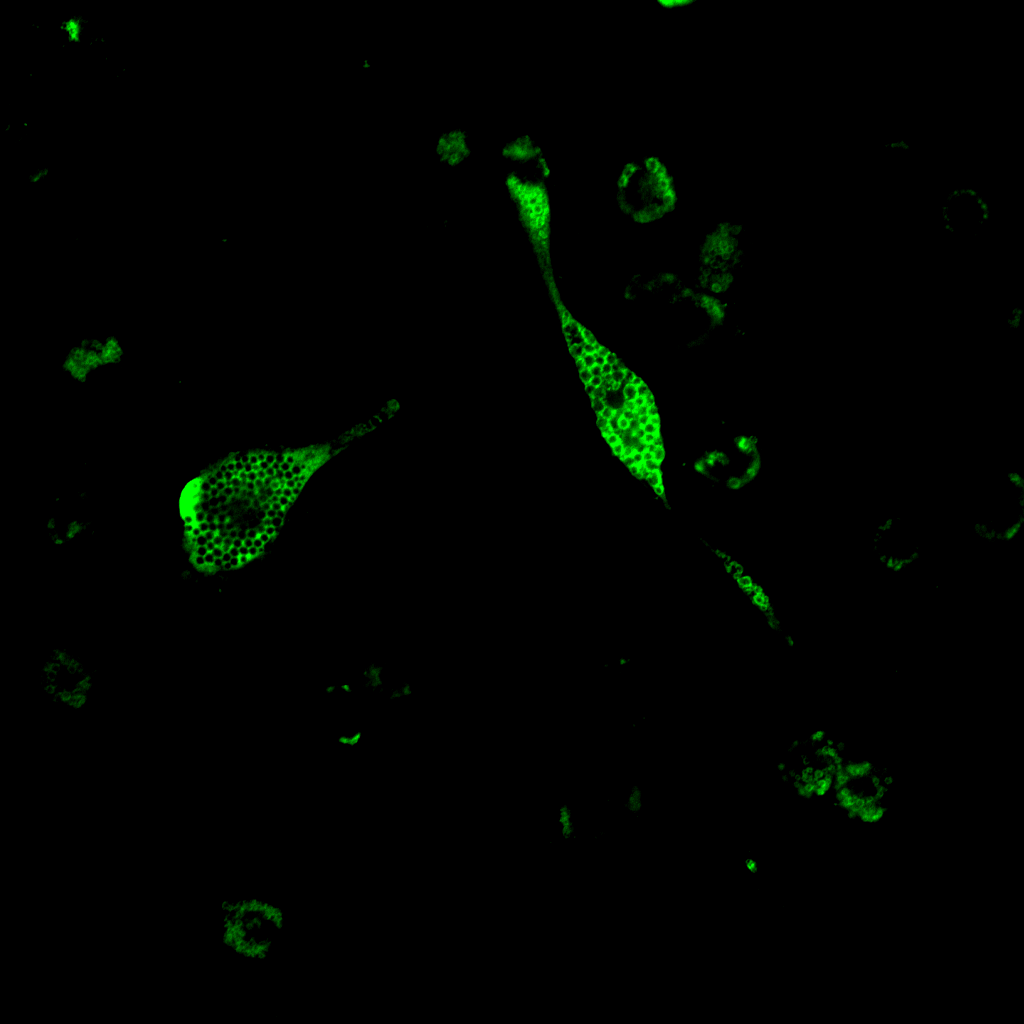

Supplement: S1 File — (ZIP) [file pone.0240762.s001.zip › SI Files Oct 2019/Fig2/ADFR stain/Hcy+folate/3/3--2.tif]

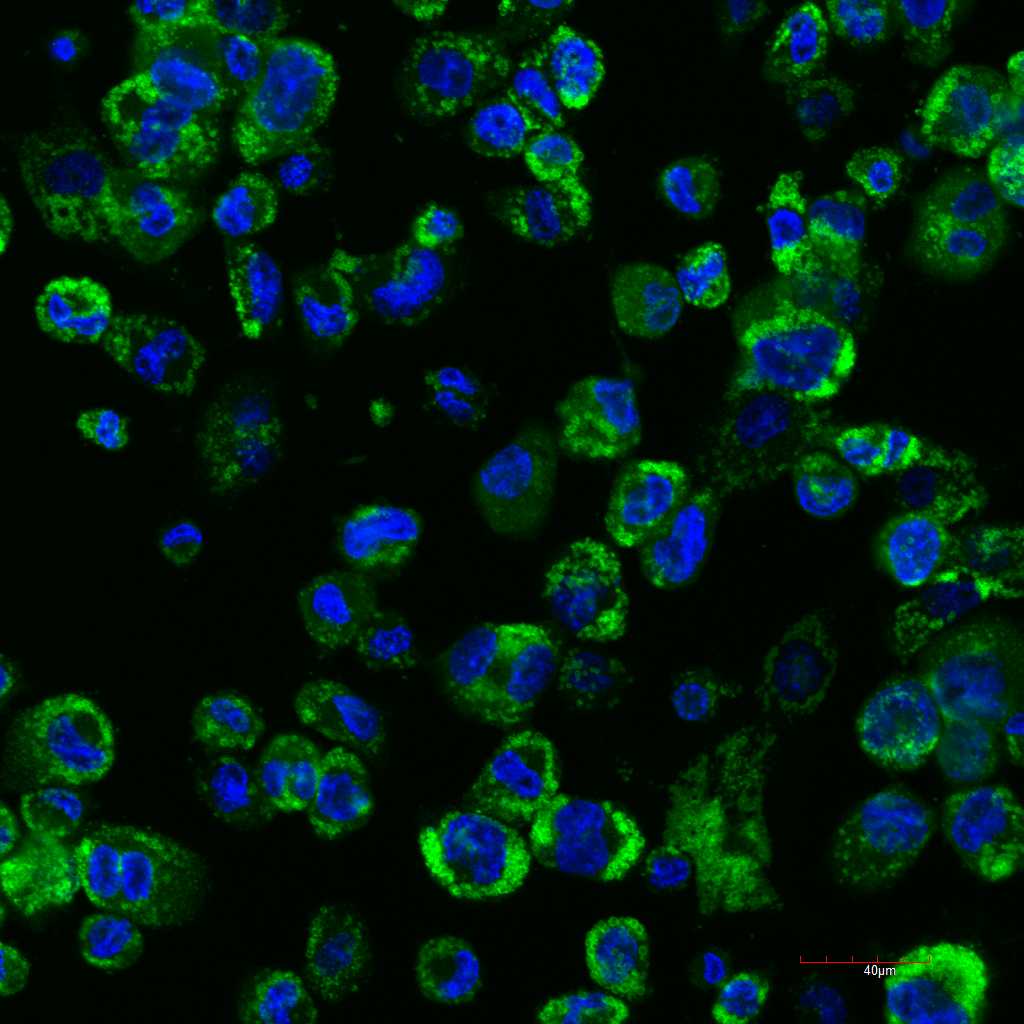

Supplement: S1 File — (ZIP) [file pone.0240762.s001.zip › SI Files Oct 2019/Fig2/ADFR stain/Hcy+folate/4/4--0.tif]

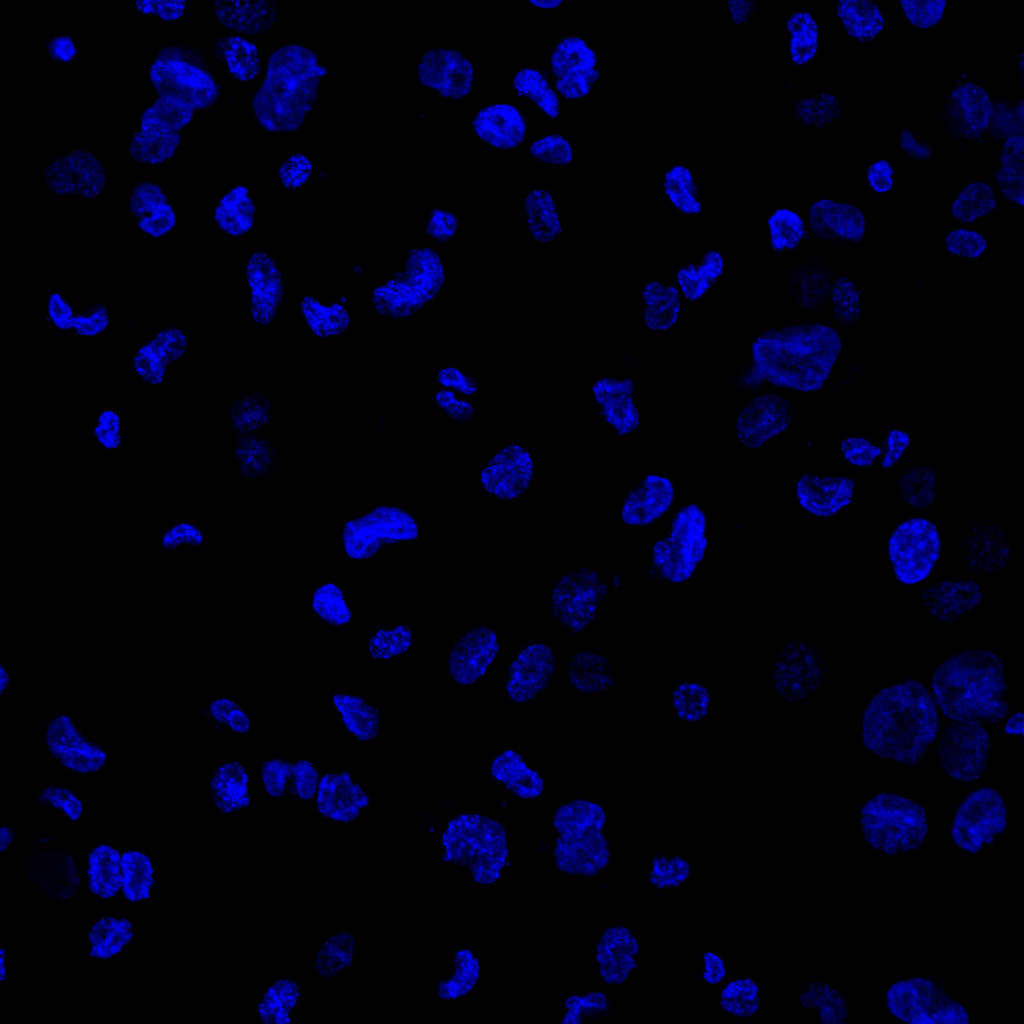

Supplement: S1 File — (ZIP) [file pone.0240762.s001.zip › SI Files Oct 2019/Fig2/ADFR stain/Hcy+folate/4/4--1.tif]

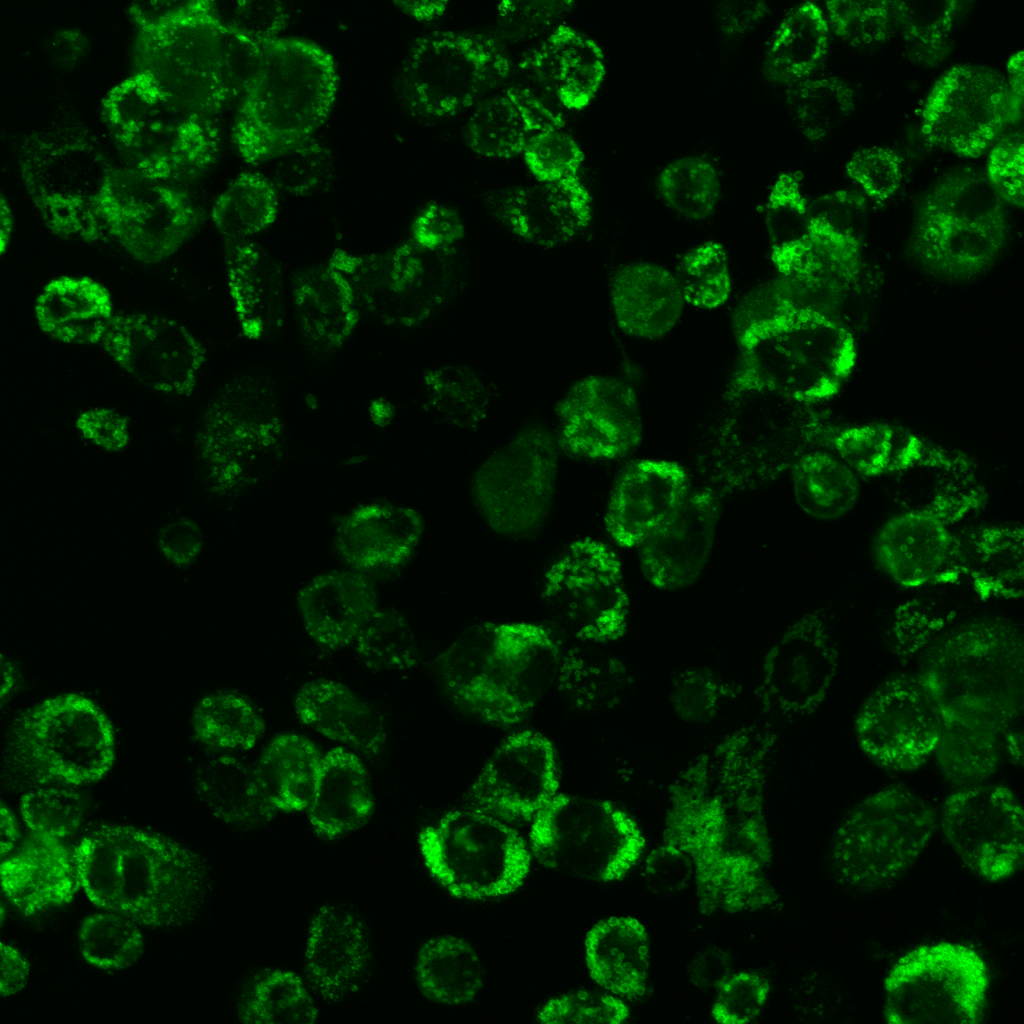

Supplement: S1 File — (ZIP) [file pone.0240762.s001.zip › SI Files Oct 2019/Fig2/ADFR stain/Hcy+folate/4/4--2.tif]

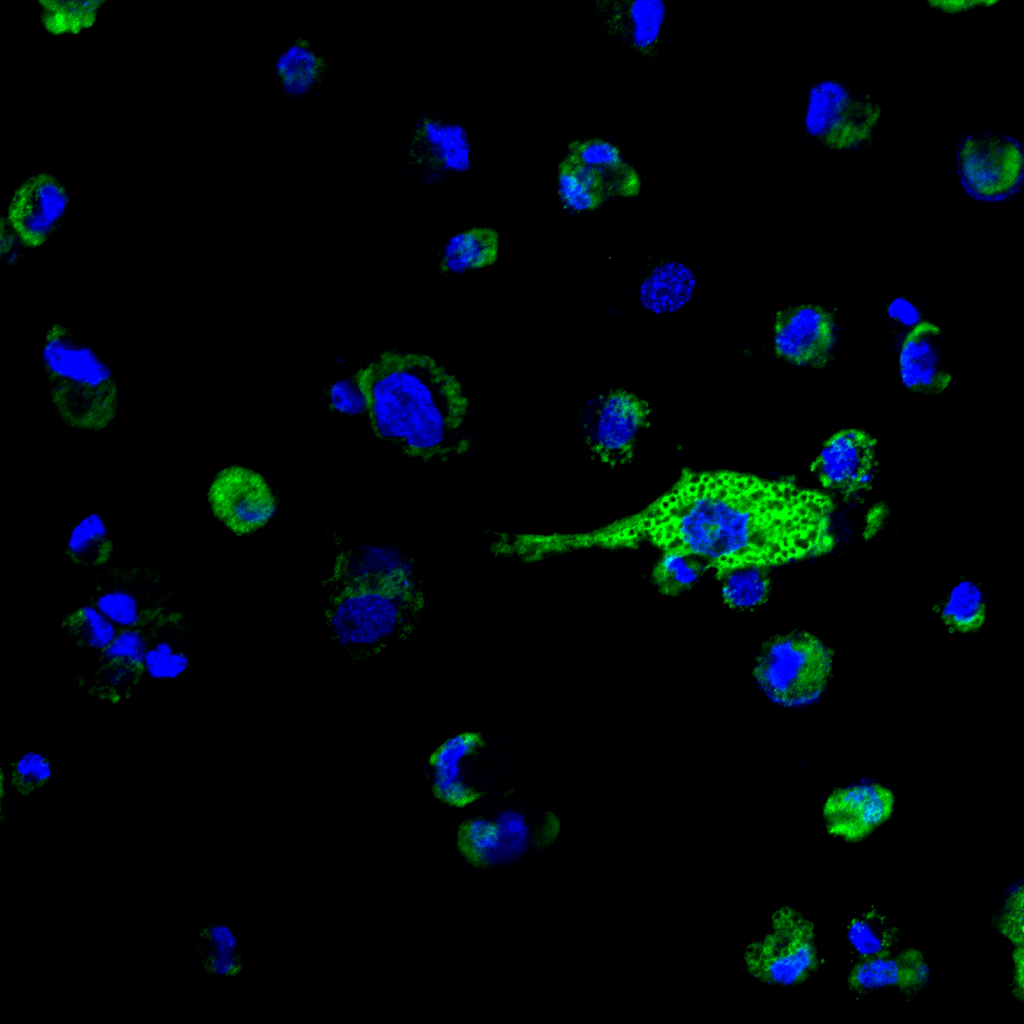

Supplement: S1 File — (ZIP) [file pone.0240762.s001.zip › SI Files Oct 2019/Fig2/ADFR stain/Hcy+folate/5/5--0.tif]

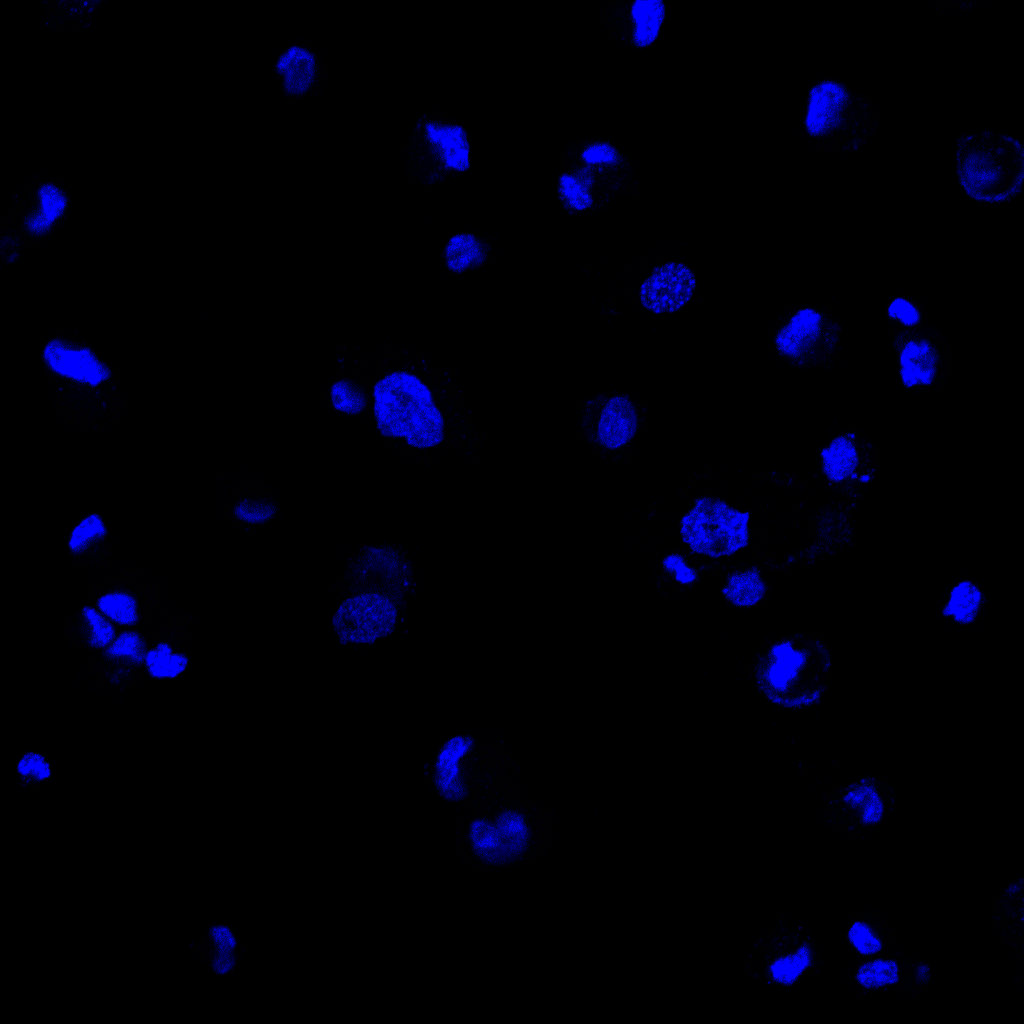

Supplement: S1 File — (ZIP) [file pone.0240762.s001.zip › SI Files Oct 2019/Fig2/ADFR stain/Hcy+folate/5/5--1.tif]

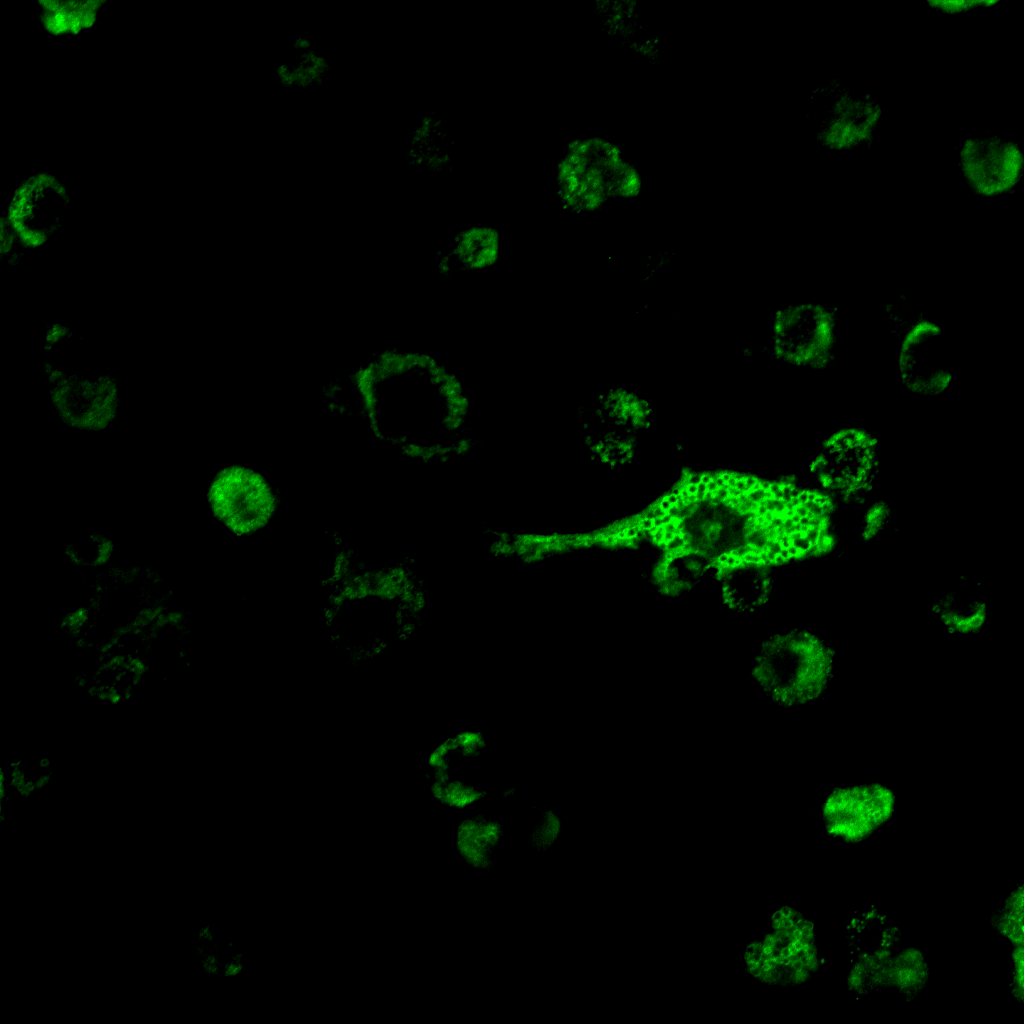

Supplement: S1 File — (ZIP) [file pone.0240762.s001.zip › SI Files Oct 2019/Fig2/ADFR stain/Hcy+folate/5/5--2.tif]

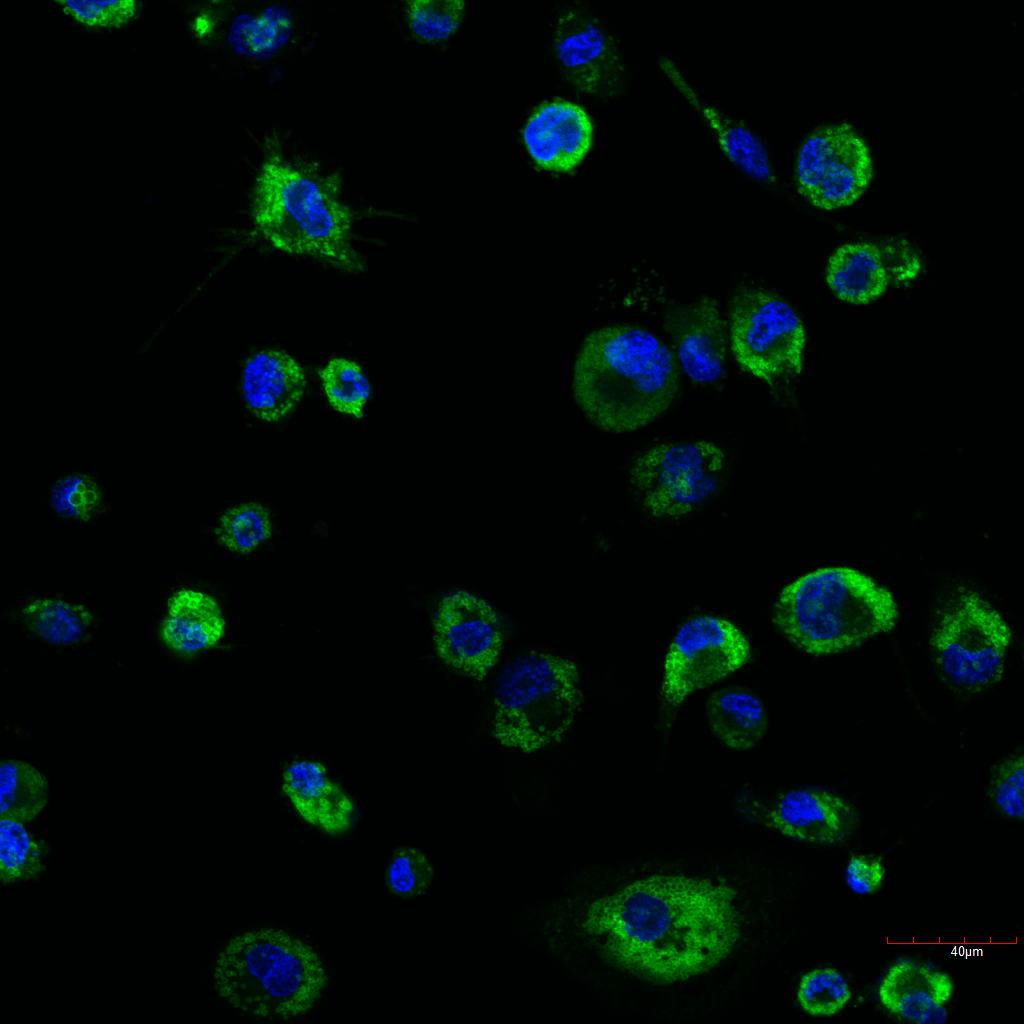

Supplement: S1 File — (ZIP) [file pone.0240762.s001.zip › SI Files Oct 2019/Fig2/ADFR stain/Hcy+folate/6/6--0.tif]

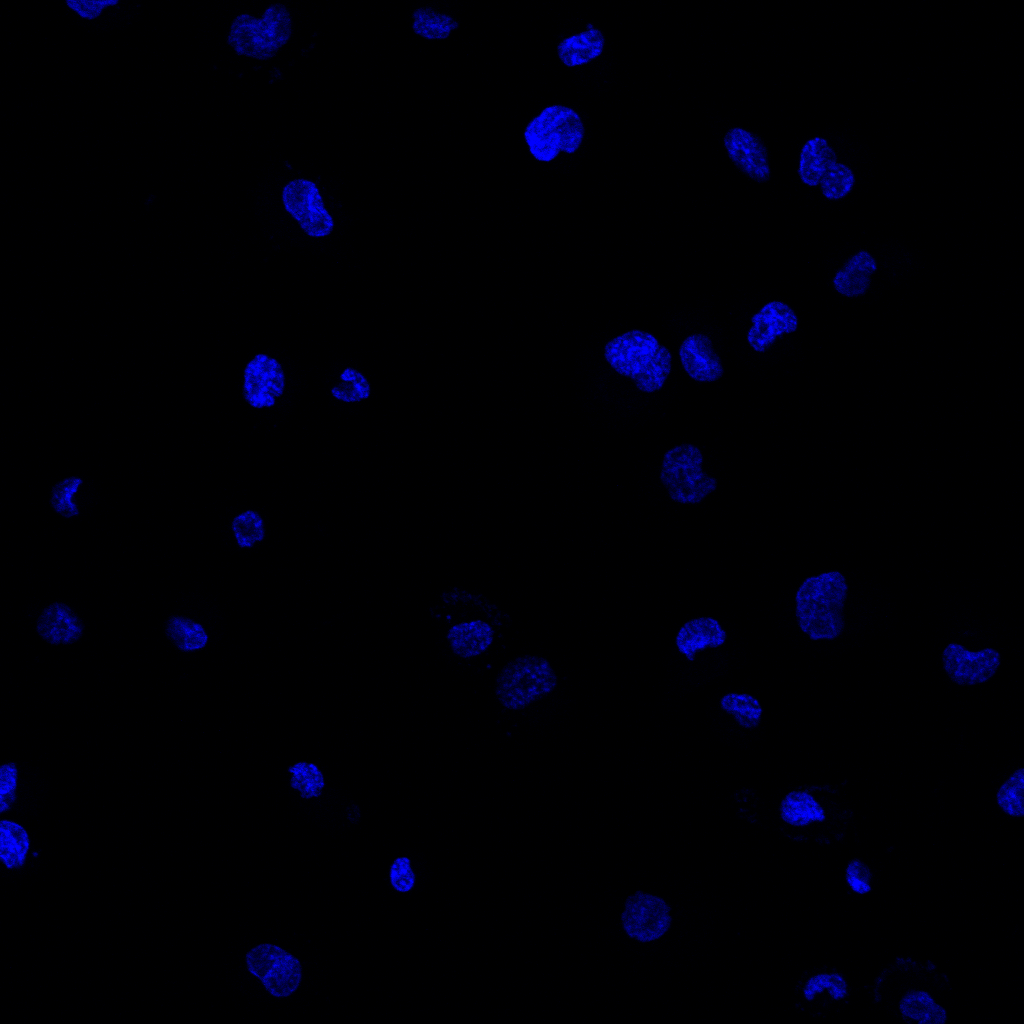

Supplement: S1 File — (ZIP) [file pone.0240762.s001.zip › SI Files Oct 2019/Fig2/ADFR stain/Hcy+folate/6/6--1.tif]

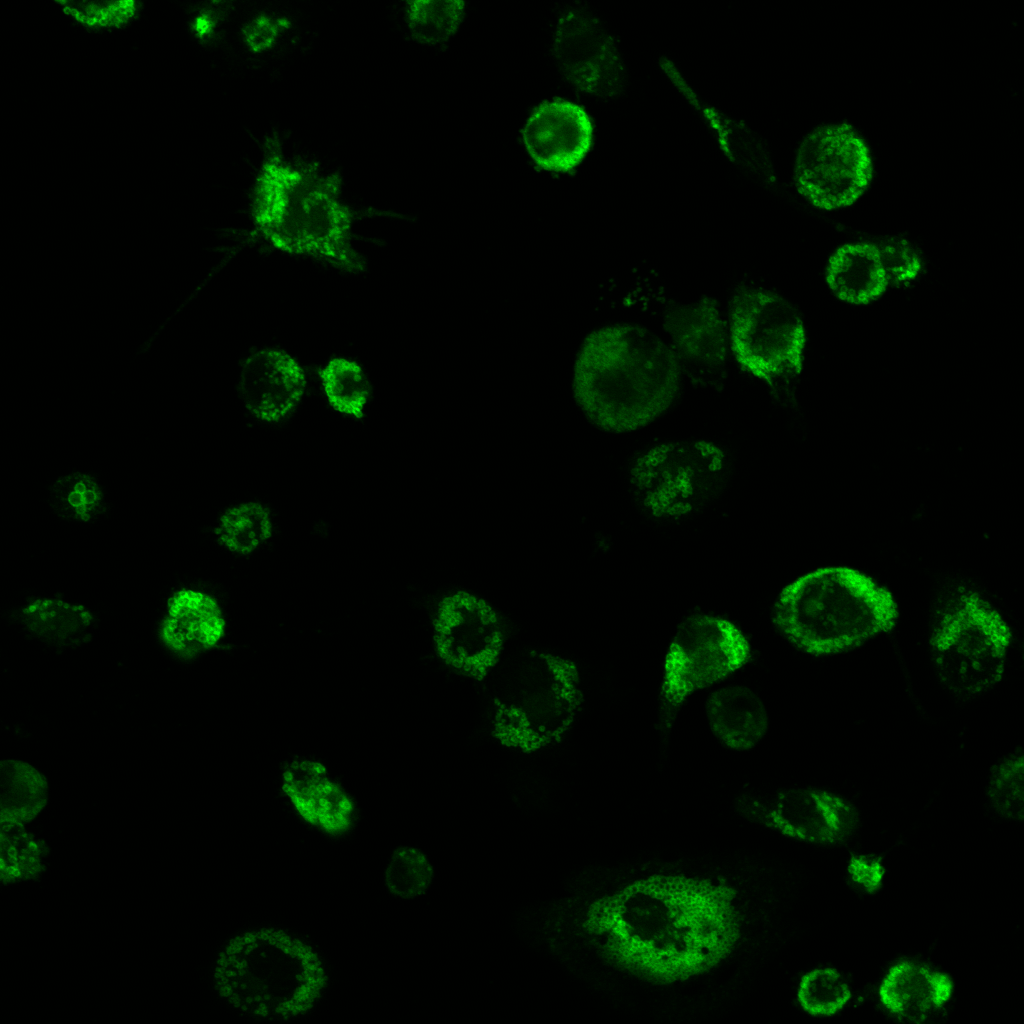

Supplement: S1 File — (ZIP) [file pone.0240762.s001.zip › SI Files Oct 2019/Fig2/ADFR stain/Hcy+folate/6/6--2.tif]

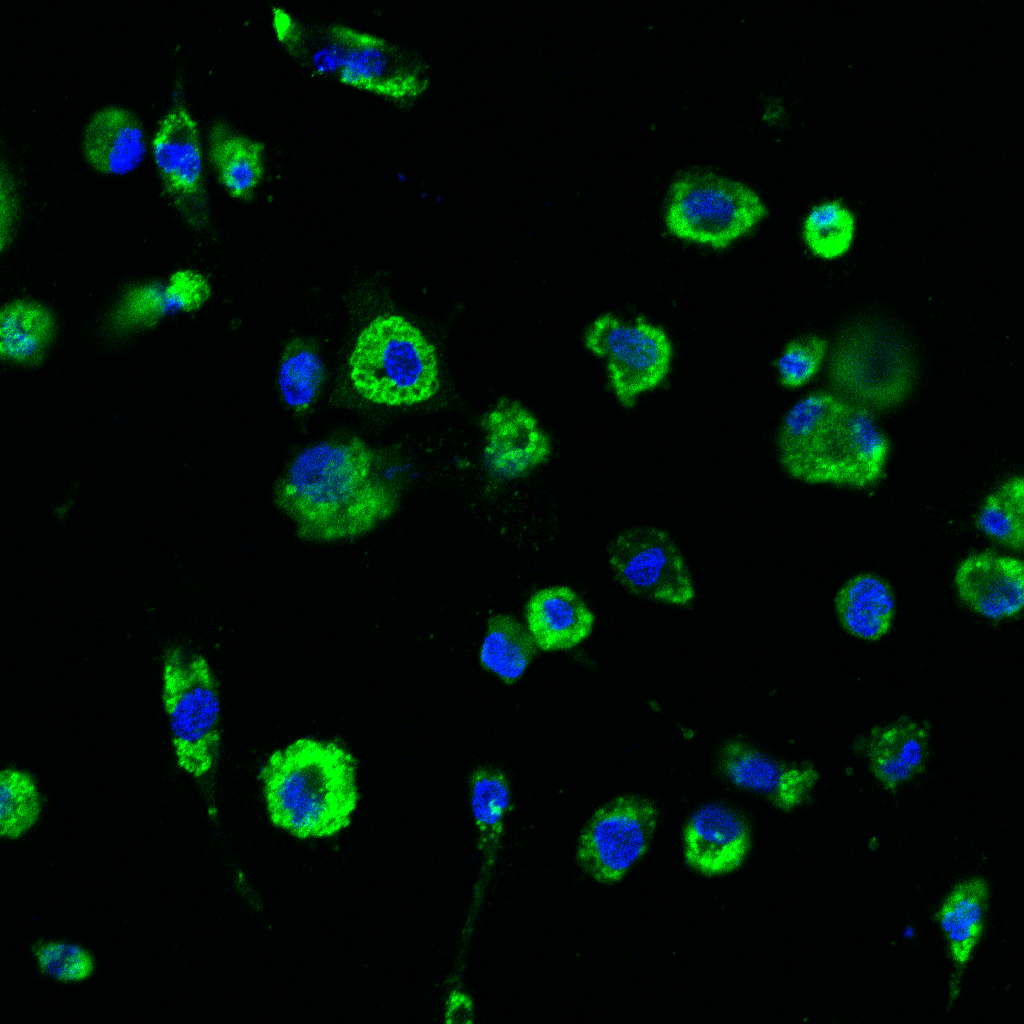

Supplement: S1 File — (ZIP) [file pone.0240762.s001.zip › SI Files Oct 2019/Fig2/ADFR stain/Hcy/1/1--0.tif]

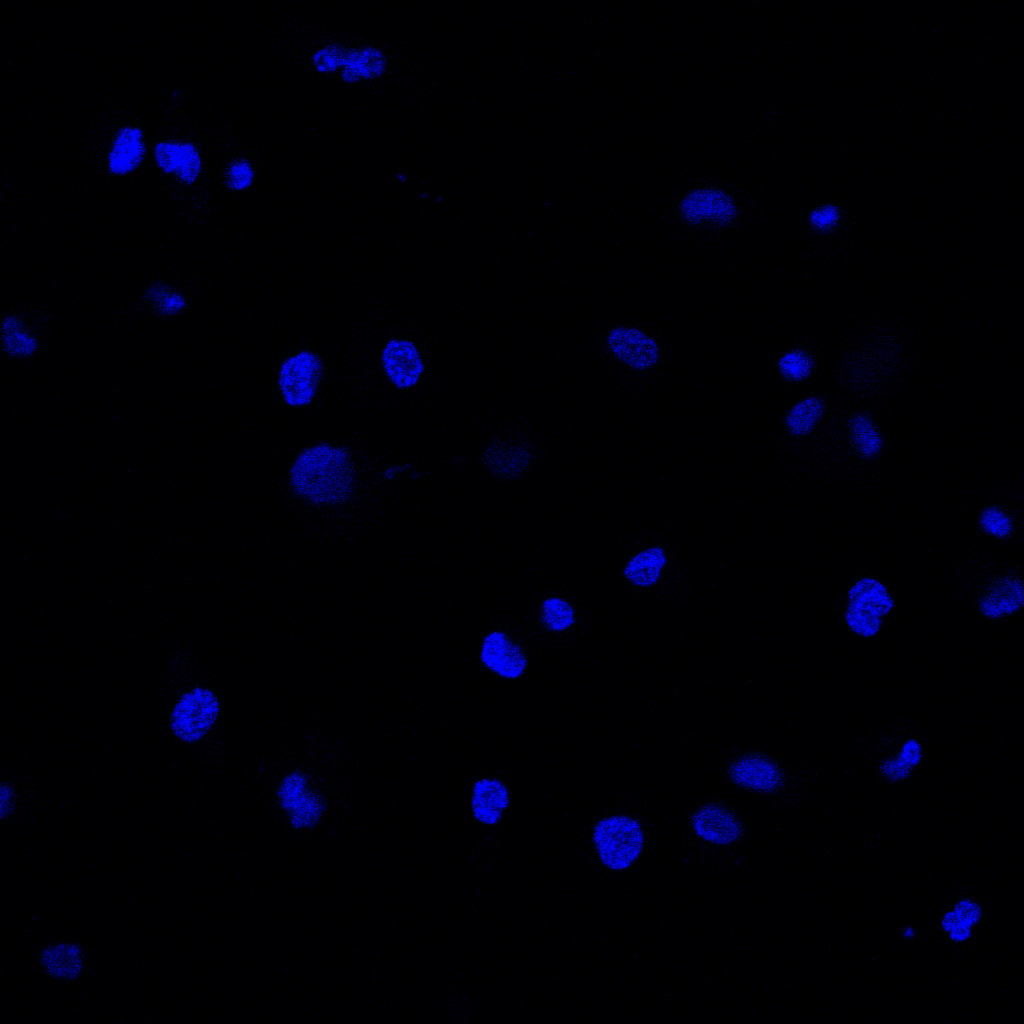

Supplement: S1 File — (ZIP) [file pone.0240762.s001.zip › SI Files Oct 2019/Fig2/ADFR stain/Hcy/1/1--1.tif]

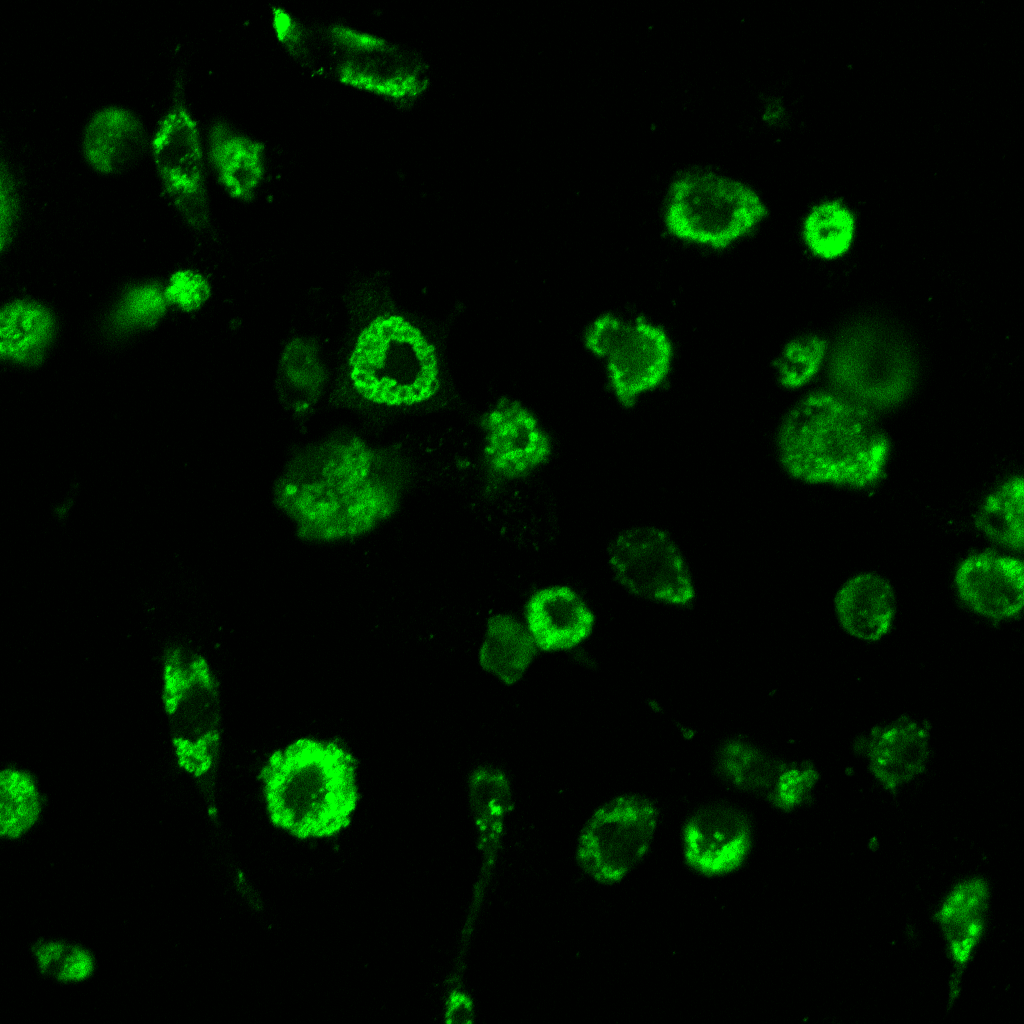

Supplement: S1 File — (ZIP) [file pone.0240762.s001.zip › SI Files Oct 2019/Fig2/ADFR stain/Hcy/1/1--2.tif]

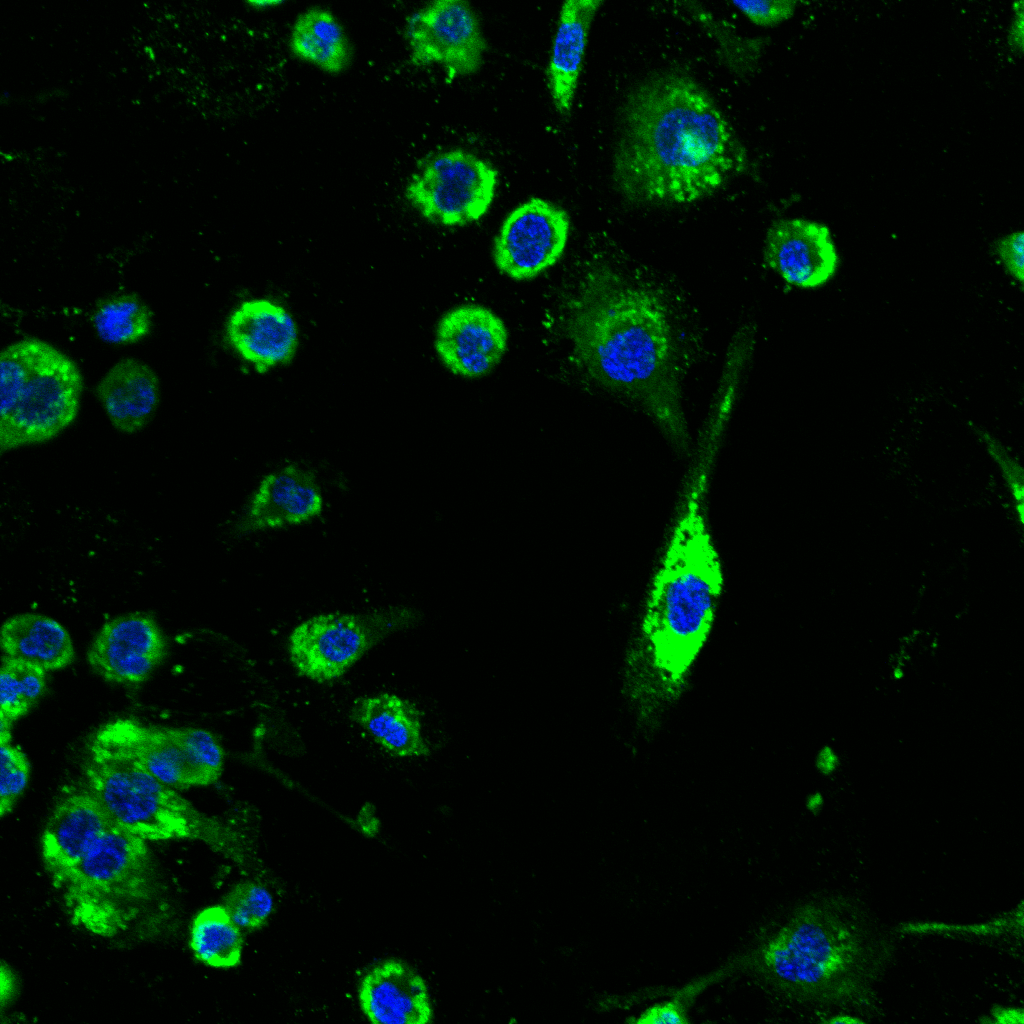

Supplement: S1 File — (ZIP) [file pone.0240762.s001.zip › SI Files Oct 2019/Fig2/ADFR stain/Hcy/2/2--0_.tif]

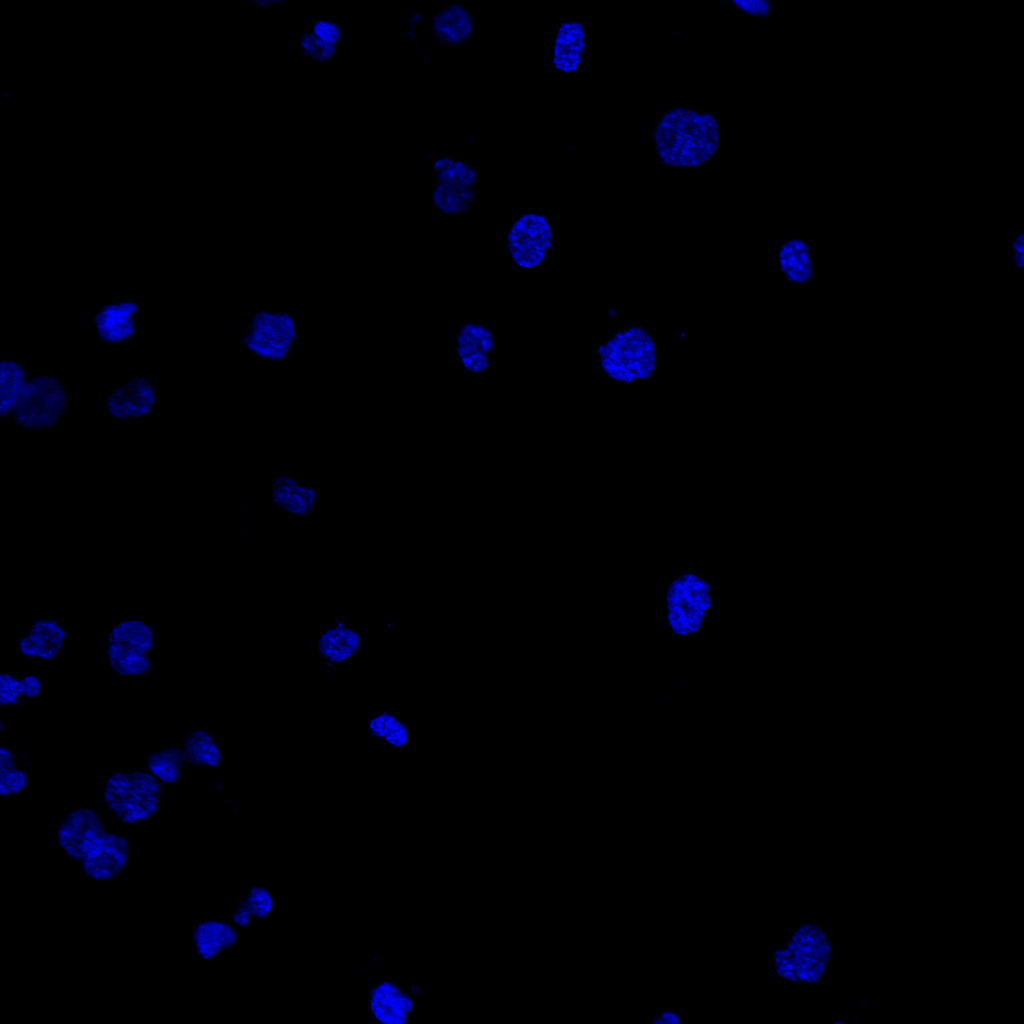

Supplement: S1 File — (ZIP) [file pone.0240762.s001.zip › SI Files Oct 2019/Fig2/ADFR stain/Hcy/2/2--1.tif]

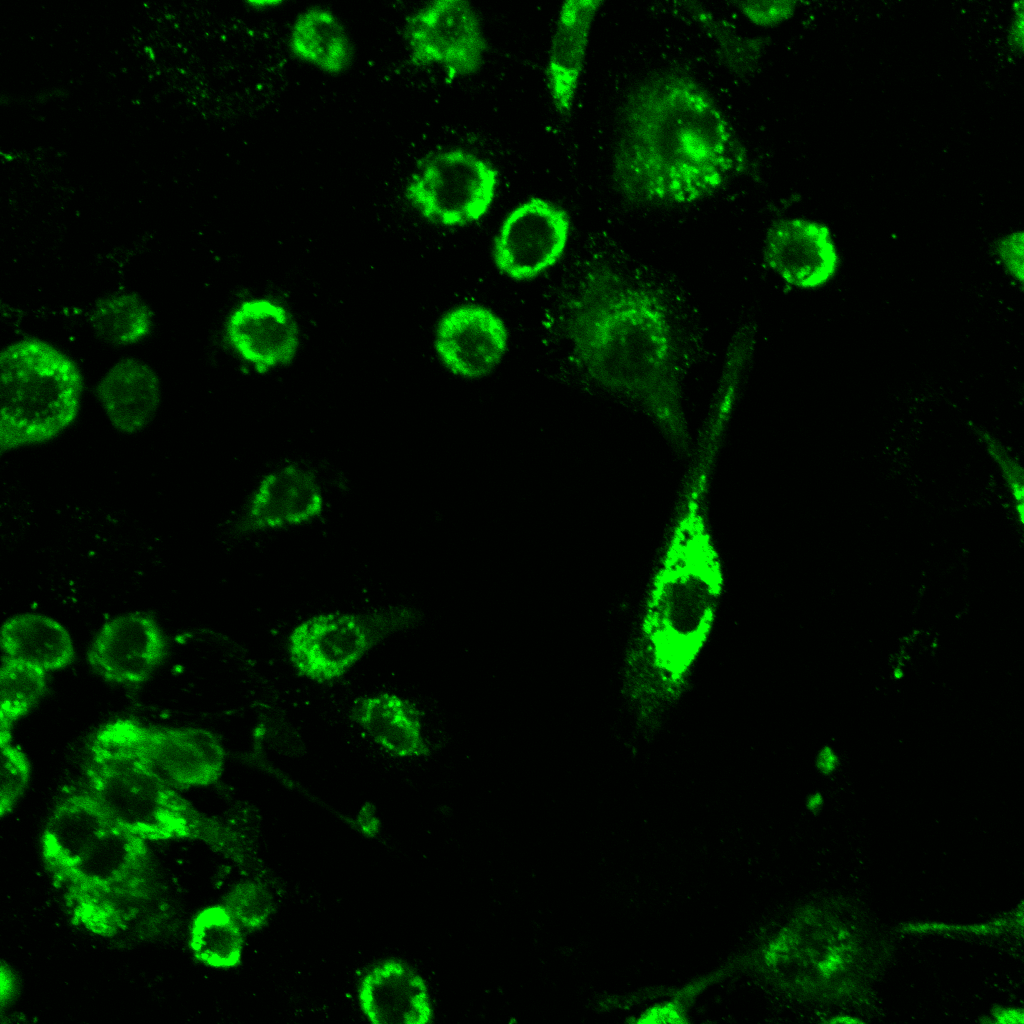

Supplement: S1 File — (ZIP) [file pone.0240762.s001.zip › SI Files Oct 2019/Fig2/ADFR stain/Hcy/2/2--2.tif]

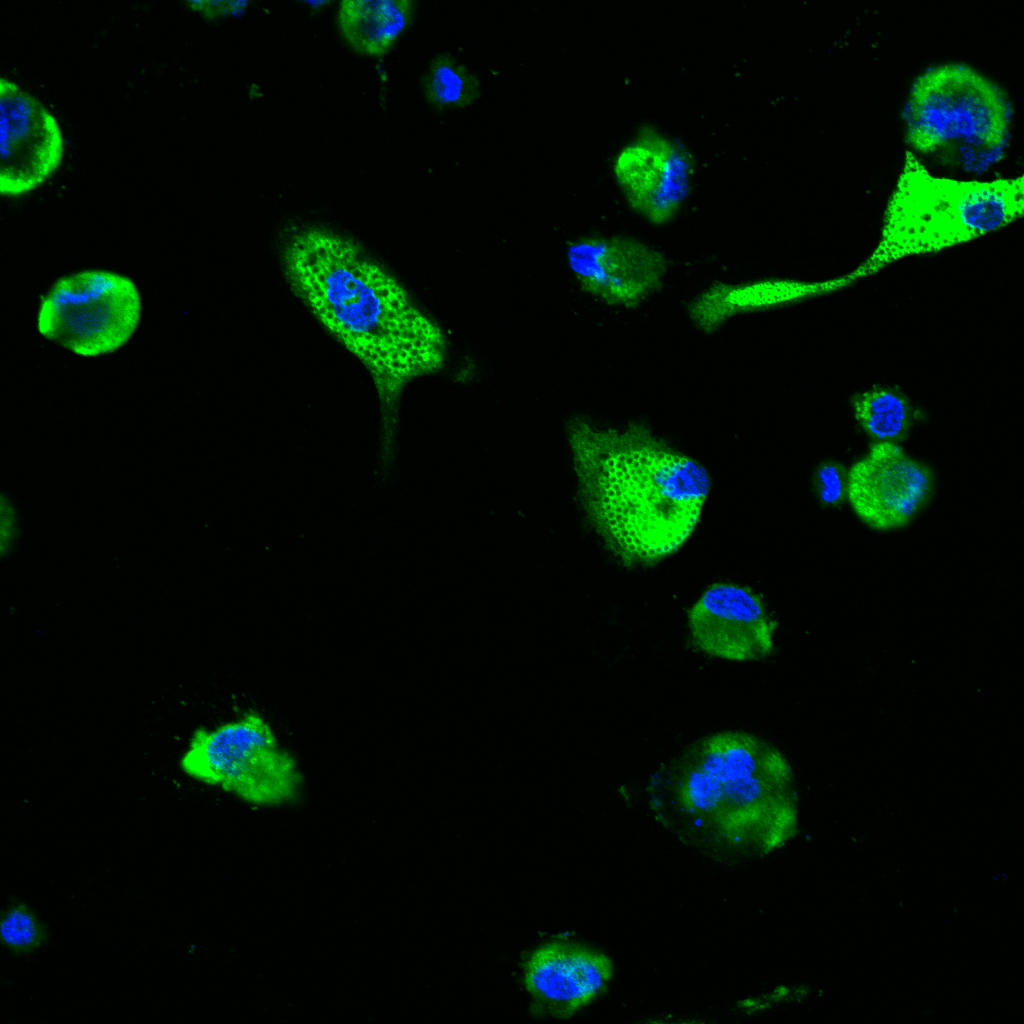

Supplement: S1 File — (ZIP) [file pone.0240762.s001.zip › SI Files Oct 2019/Fig2/ADFR stain/Hcy/3/3--0.tif]

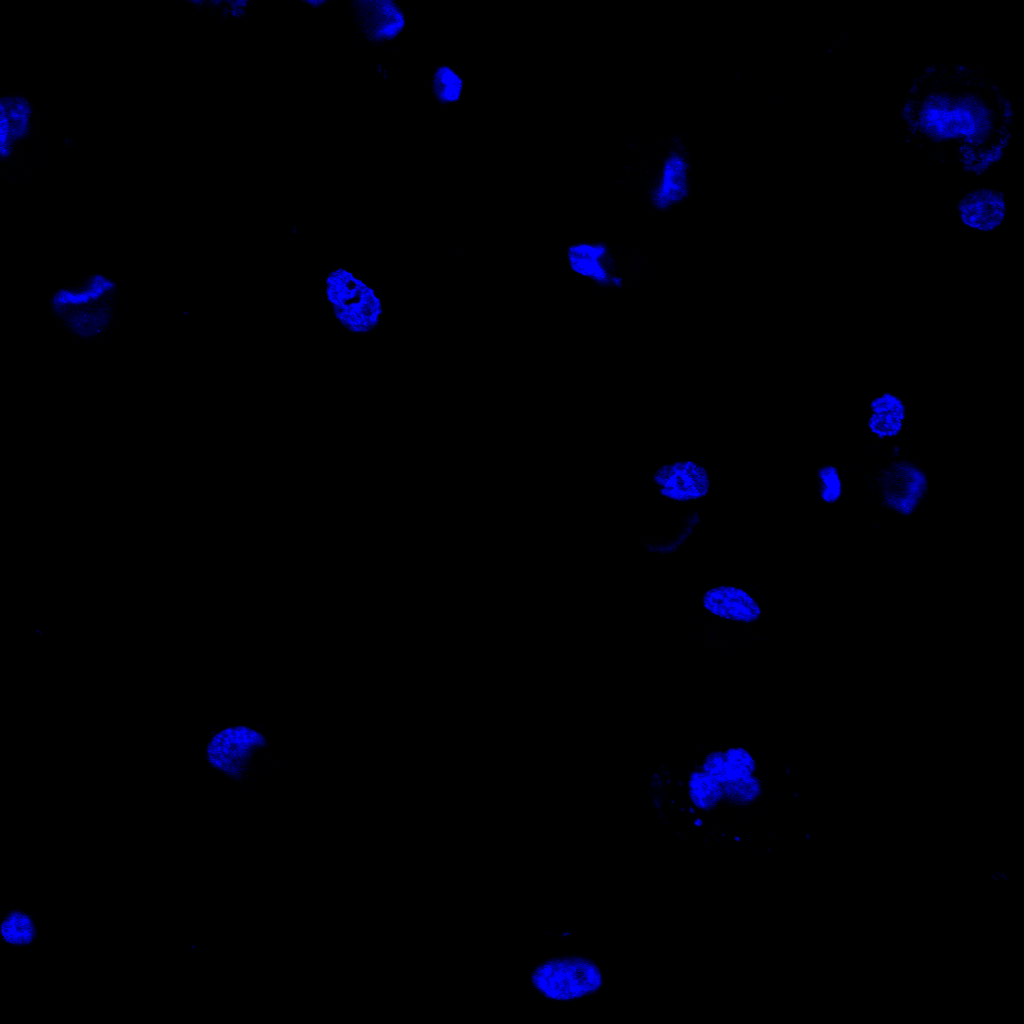

Supplement: S1 File — (ZIP) [file pone.0240762.s001.zip › SI Files Oct 2019/Fig2/ADFR stain/Hcy/3/3--1.tif]

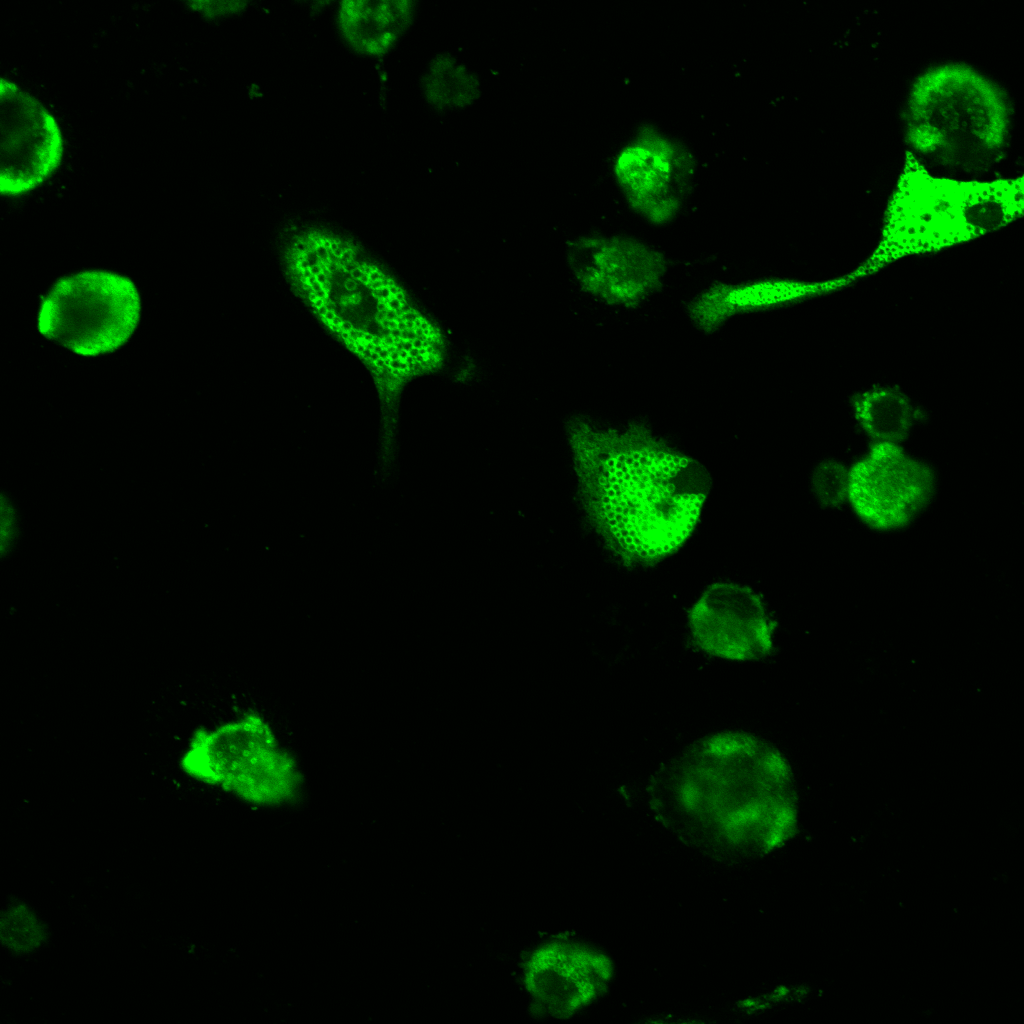

Supplement: S1 File — (ZIP) [file pone.0240762.s001.zip › SI Files Oct 2019/Fig2/ADFR stain/Hcy/3/3--2.tif]

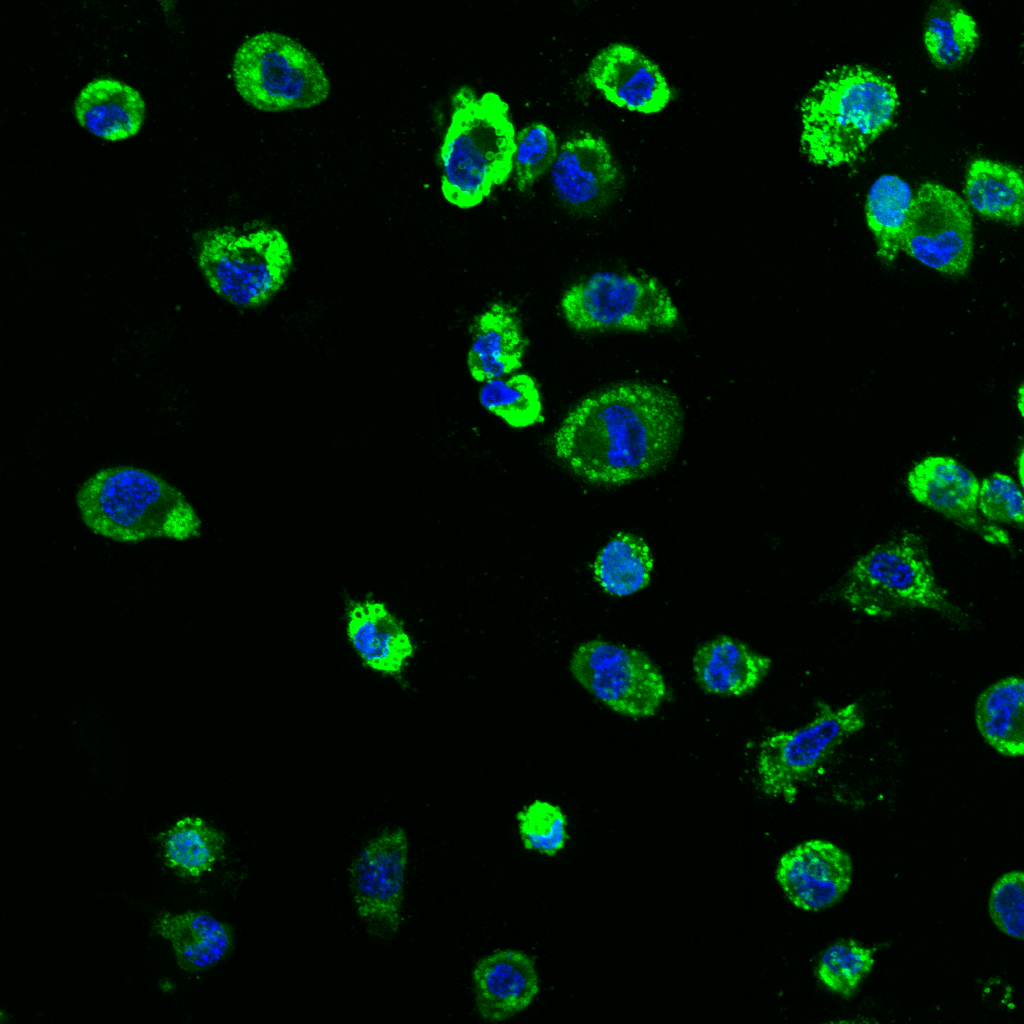

Supplement: S1 File — (ZIP) [file pone.0240762.s001.zip › SI Files Oct 2019/Fig2/ADFR stain/Hcy/4/4--0.tif]

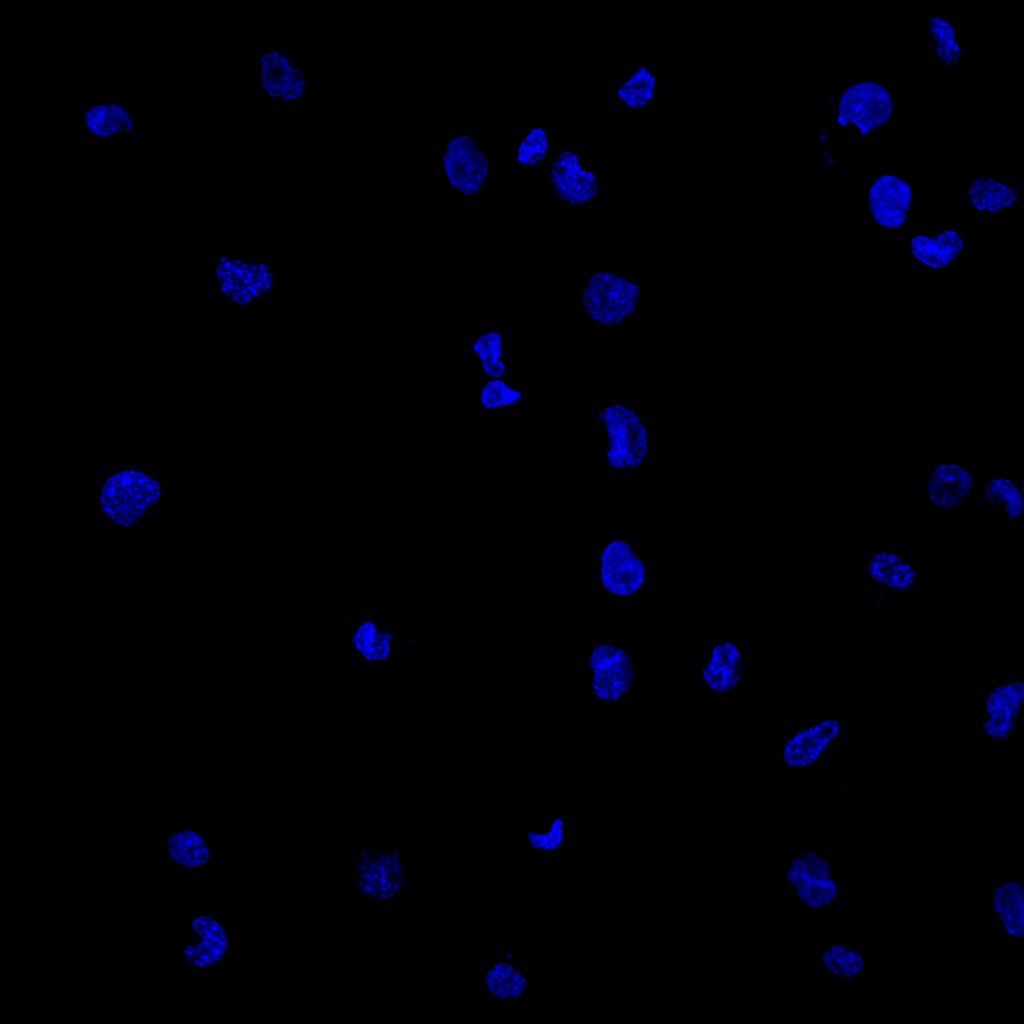

Supplement: S1 File — (ZIP) [file pone.0240762.s001.zip › SI Files Oct 2019/Fig2/ADFR stain/Hcy/4/4--1.tif]

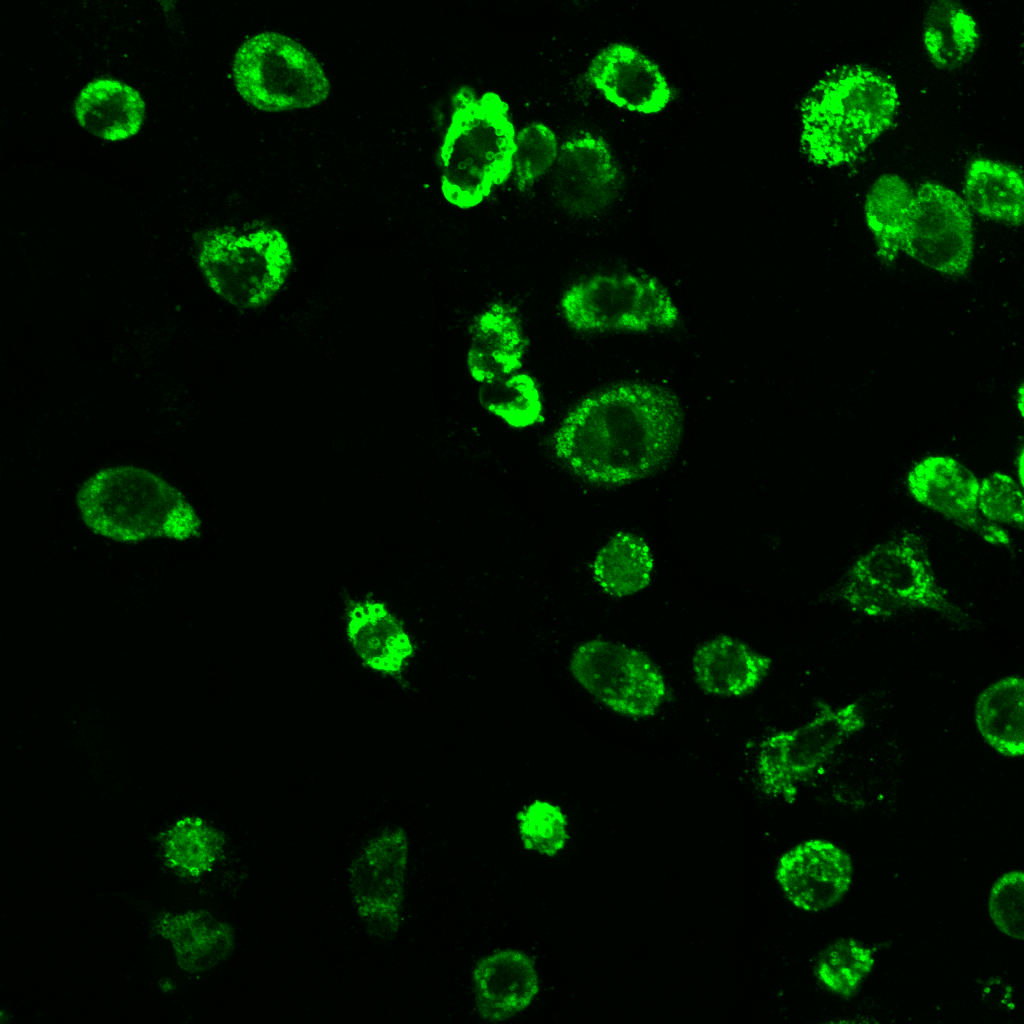

Supplement: S1 File — (ZIP) [file pone.0240762.s001.zip › SI Files Oct 2019/Fig2/ADFR stain/Hcy/4/4--2.tif]

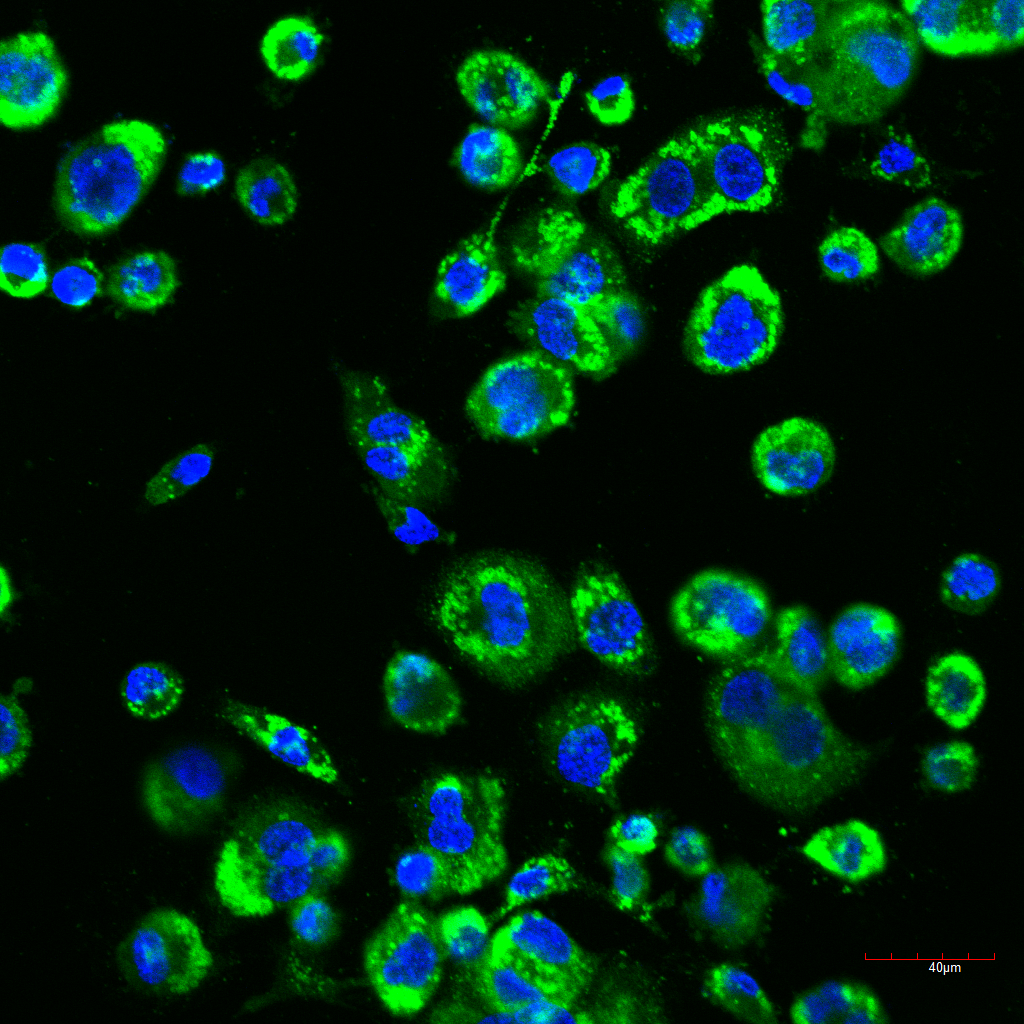

Supplement: S1 File — (ZIP) [file pone.0240762.s001.zip › SI Files Oct 2019/Fig2/ADFR stain/Hcy/5/5--0.tif]

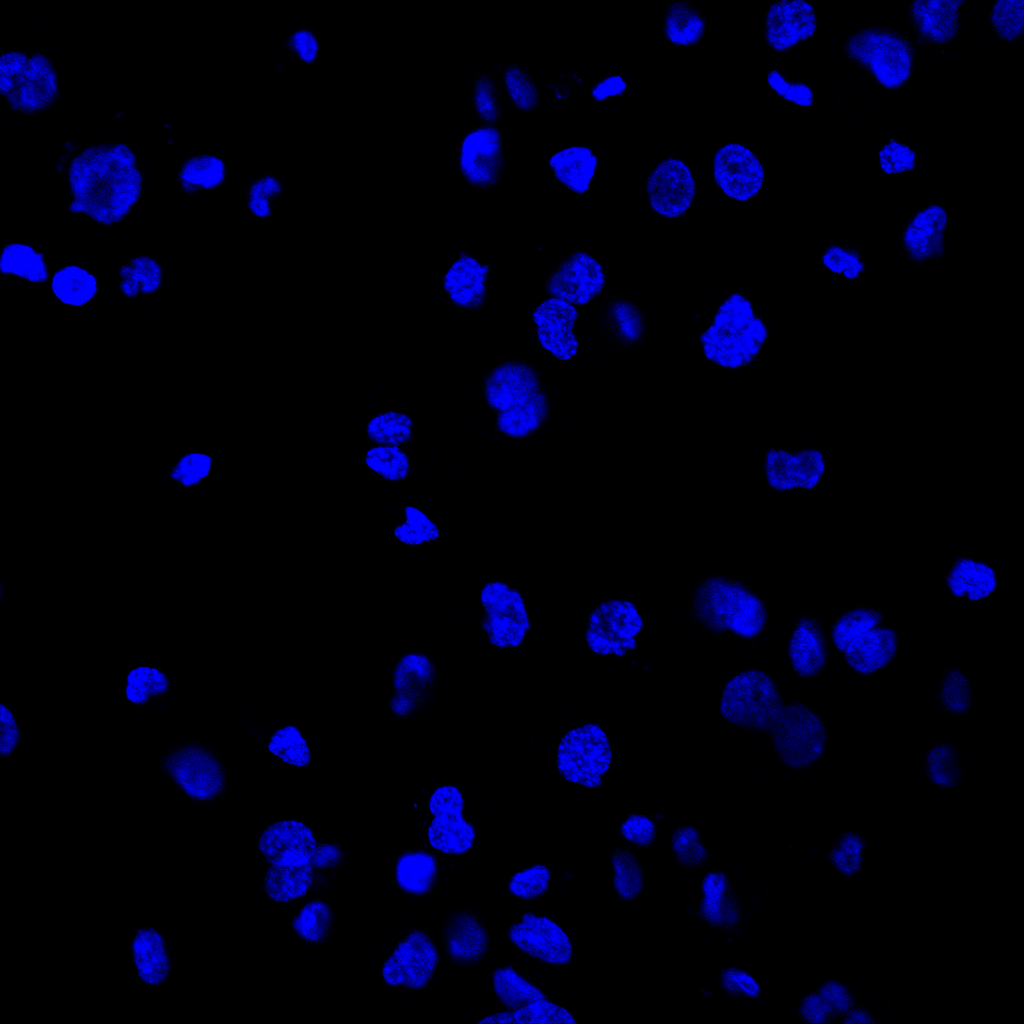

Supplement: S1 File — (ZIP) [file pone.0240762.s001.zip › SI Files Oct 2019/Fig2/ADFR stain/Hcy/5/5--1.tif]

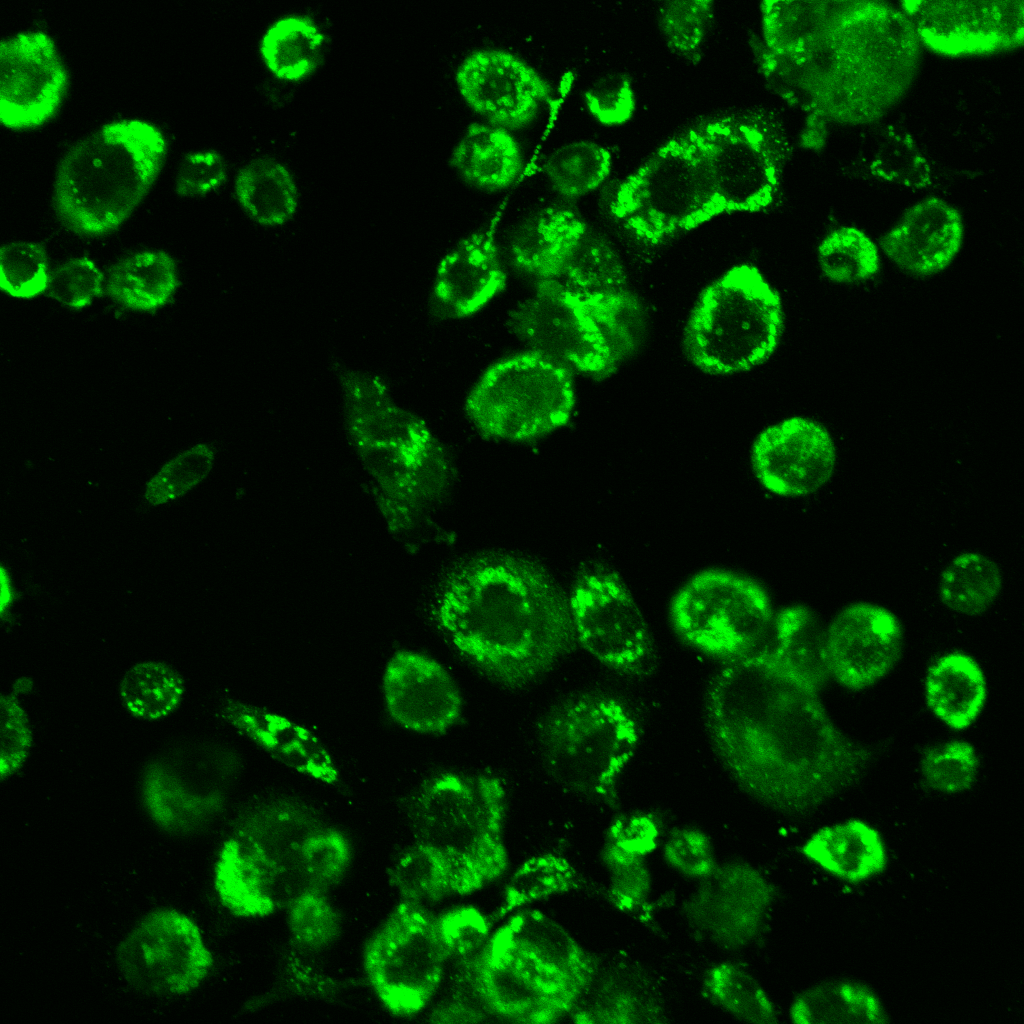

Supplement: S1 File — (ZIP) [file pone.0240762.s001.zip › SI Files Oct 2019/Fig2/ADFR stain/Hcy/5/5--2.tif]

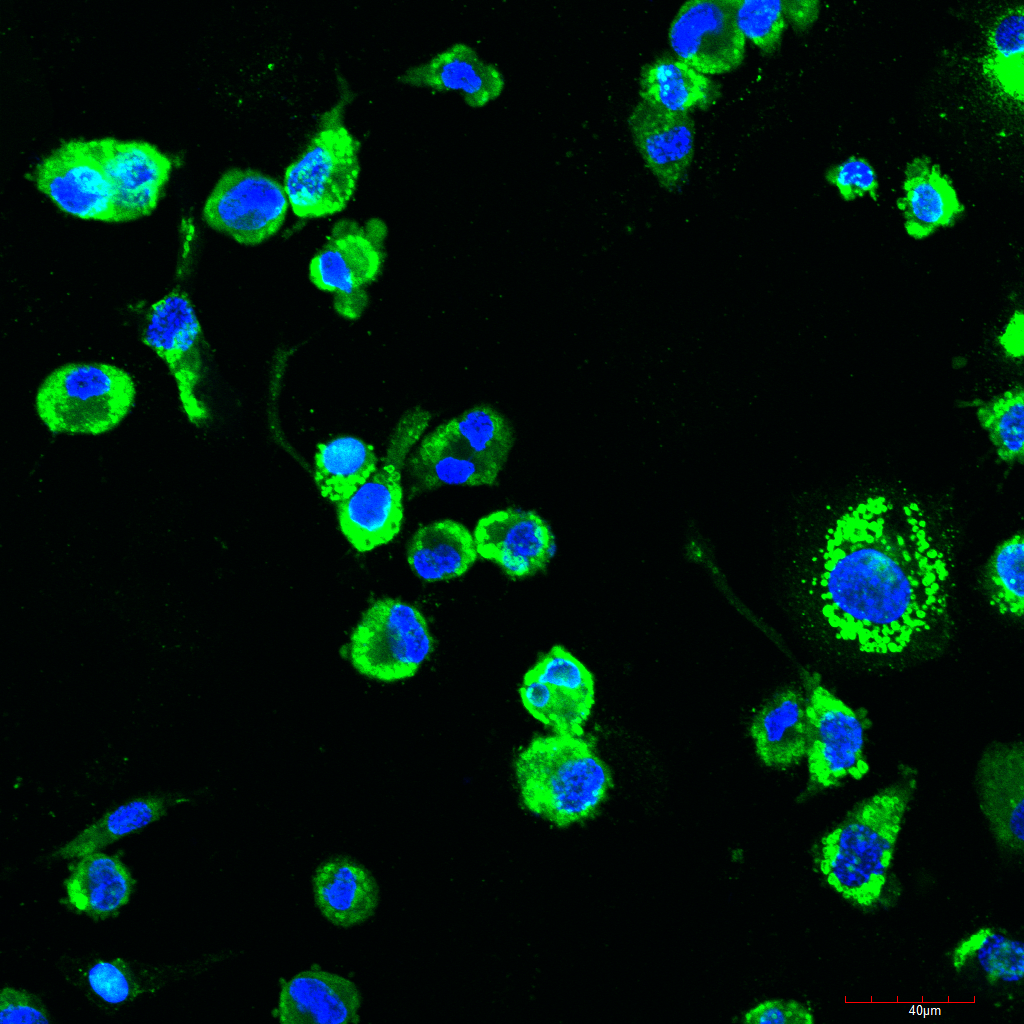

Supplement: S1 File — (ZIP) [file pone.0240762.s001.zip › SI Files Oct 2019/Fig2/ADFR stain/Hcy/6/6--0.tif]

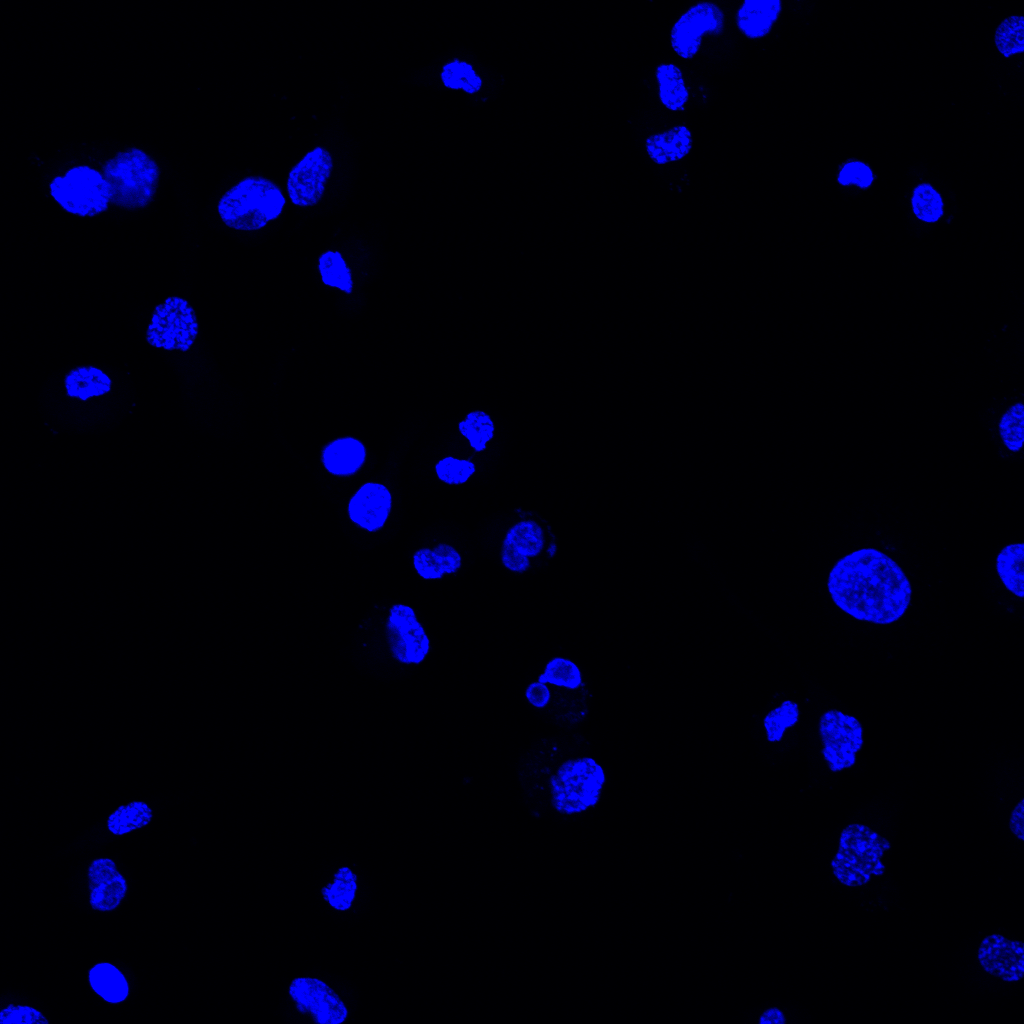

Supplement: S1 File — (ZIP) [file pone.0240762.s001.zip › SI Files Oct 2019/Fig2/ADFR stain/Hcy/6/6--1.tif]

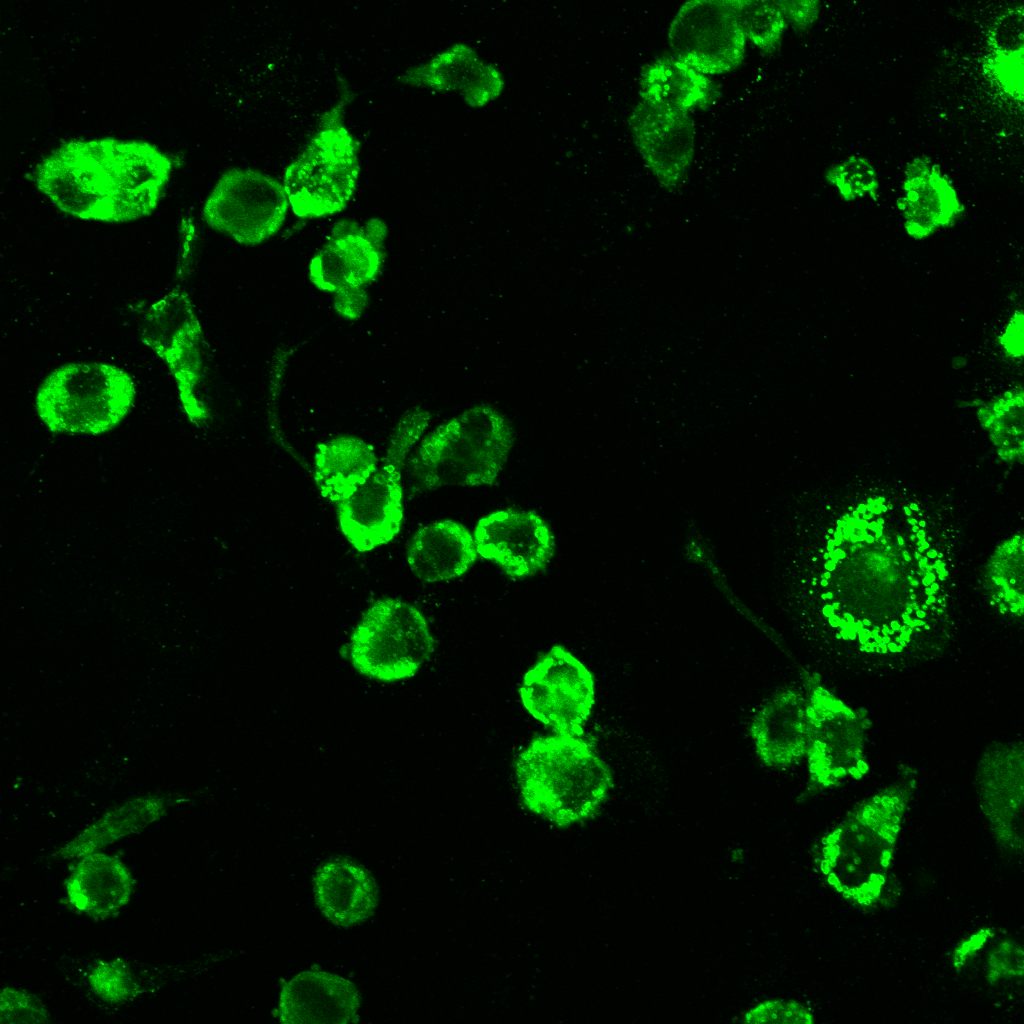

Supplement: S1 File — (ZIP) [file pone.0240762.s001.zip › SI Files Oct 2019/Fig2/ADFR stain/Hcy/6/6--2.tif]

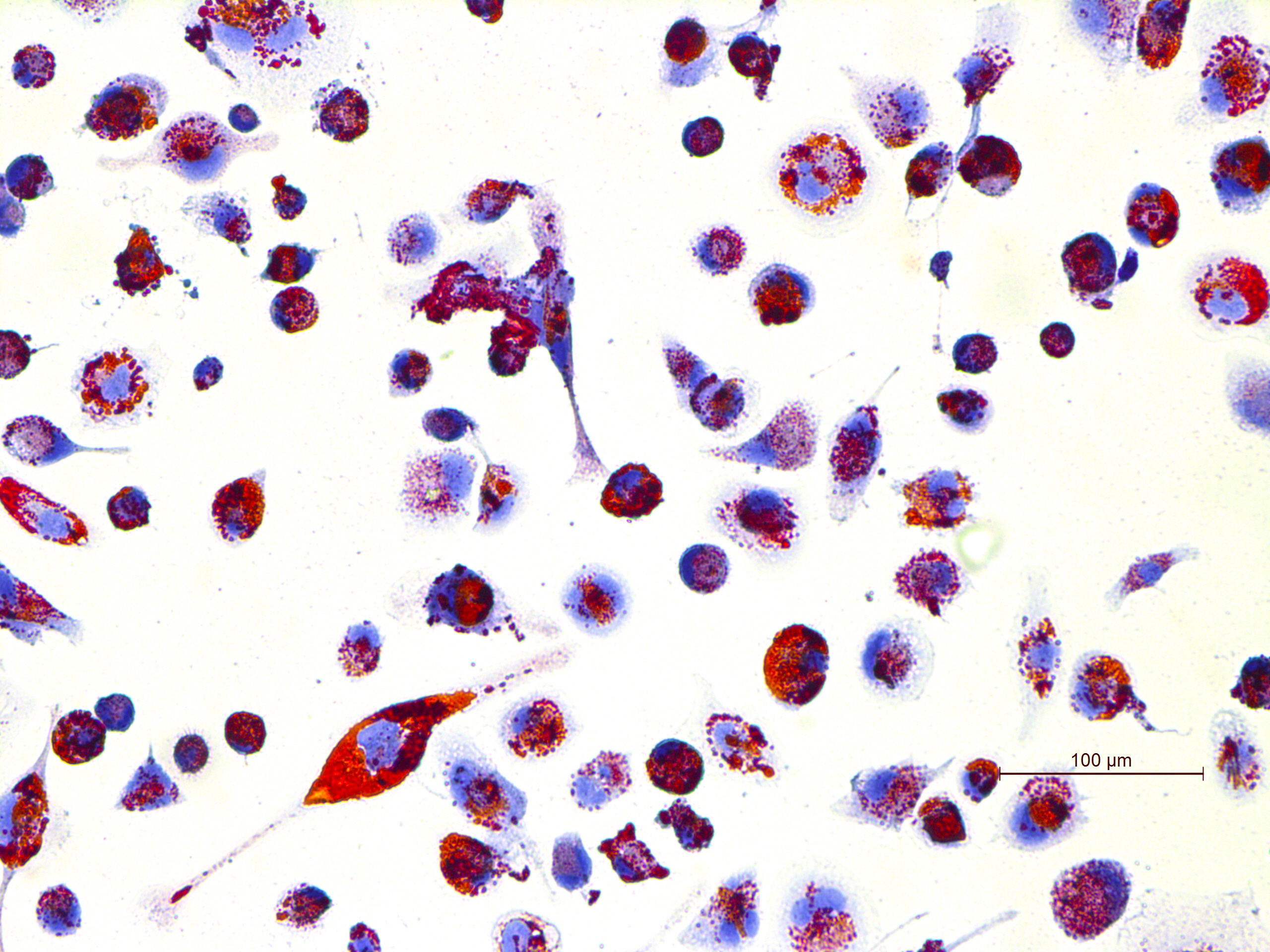

Supplement: S1 File — (ZIP) [file pone.0240762.s001.zip › SI Files Oct 2019/Fig2/oil O stain/control/1.jpg]

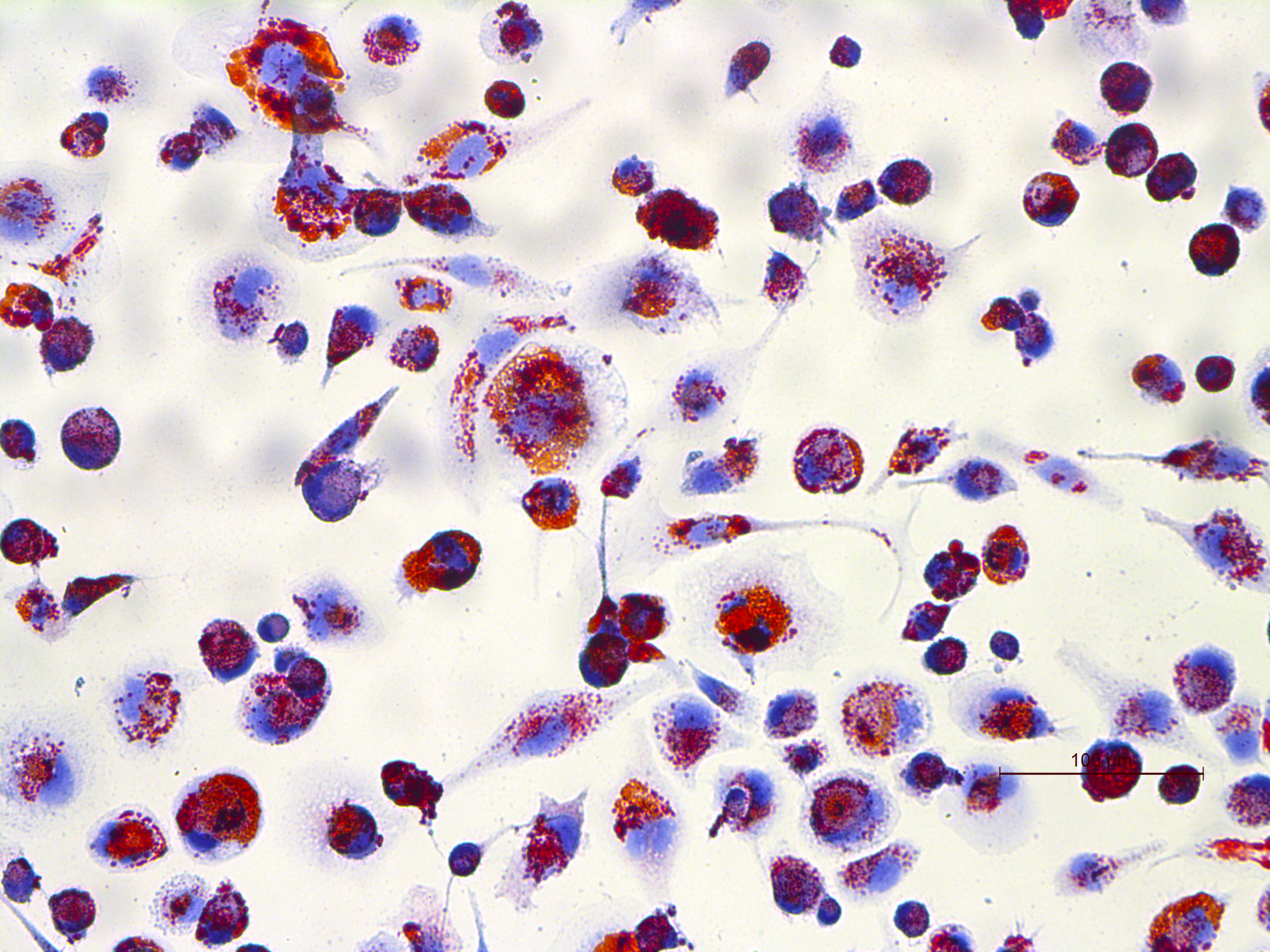

Supplement: S1 File — (ZIP) [file pone.0240762.s001.zip › SI Files Oct 2019/Fig2/oil O stain/control/2.jpg]

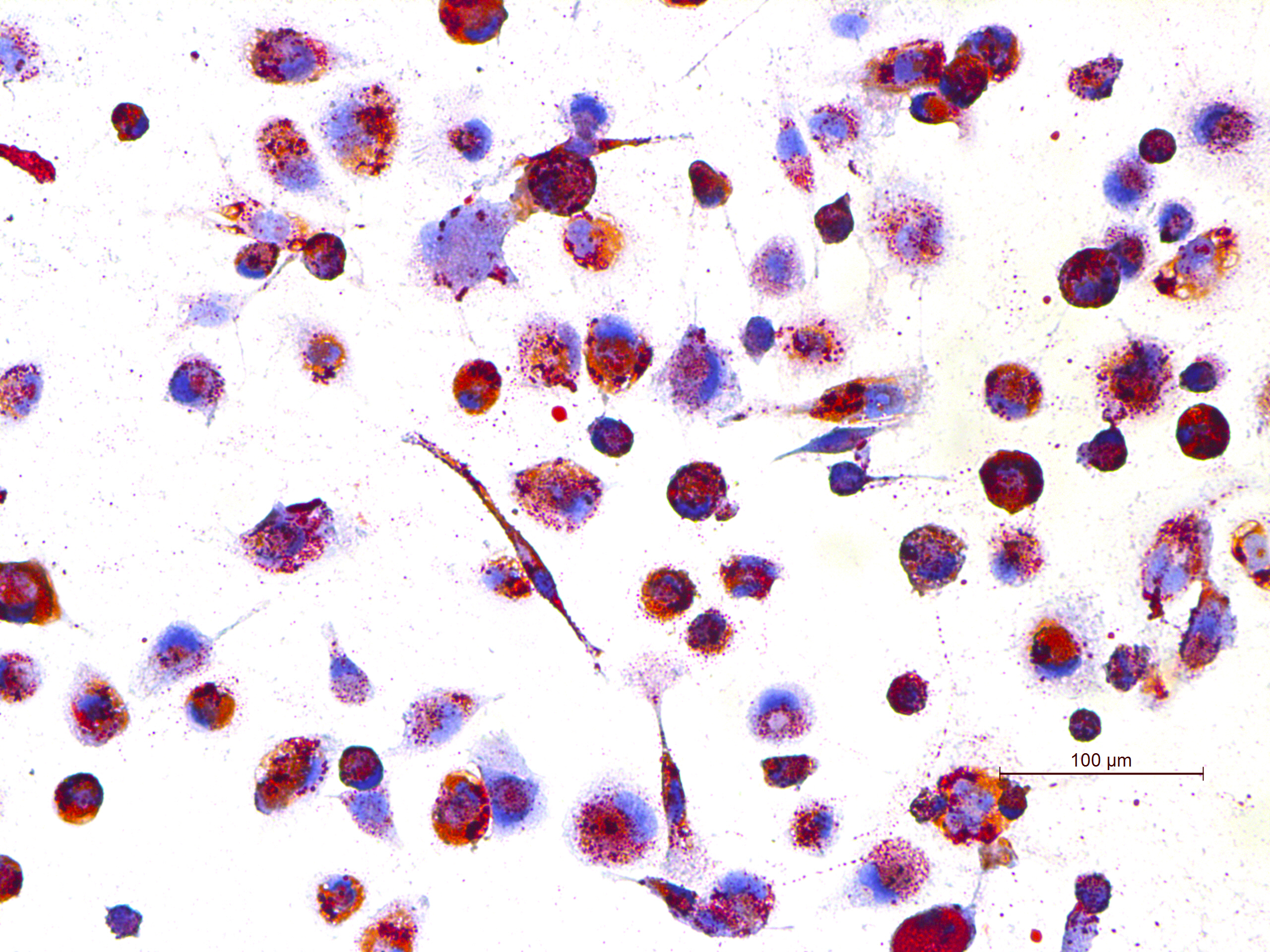

Supplement: S1 File — (ZIP) [file pone.0240762.s001.zip › SI Files Oct 2019/Fig2/oil O stain/control/3.jpg]

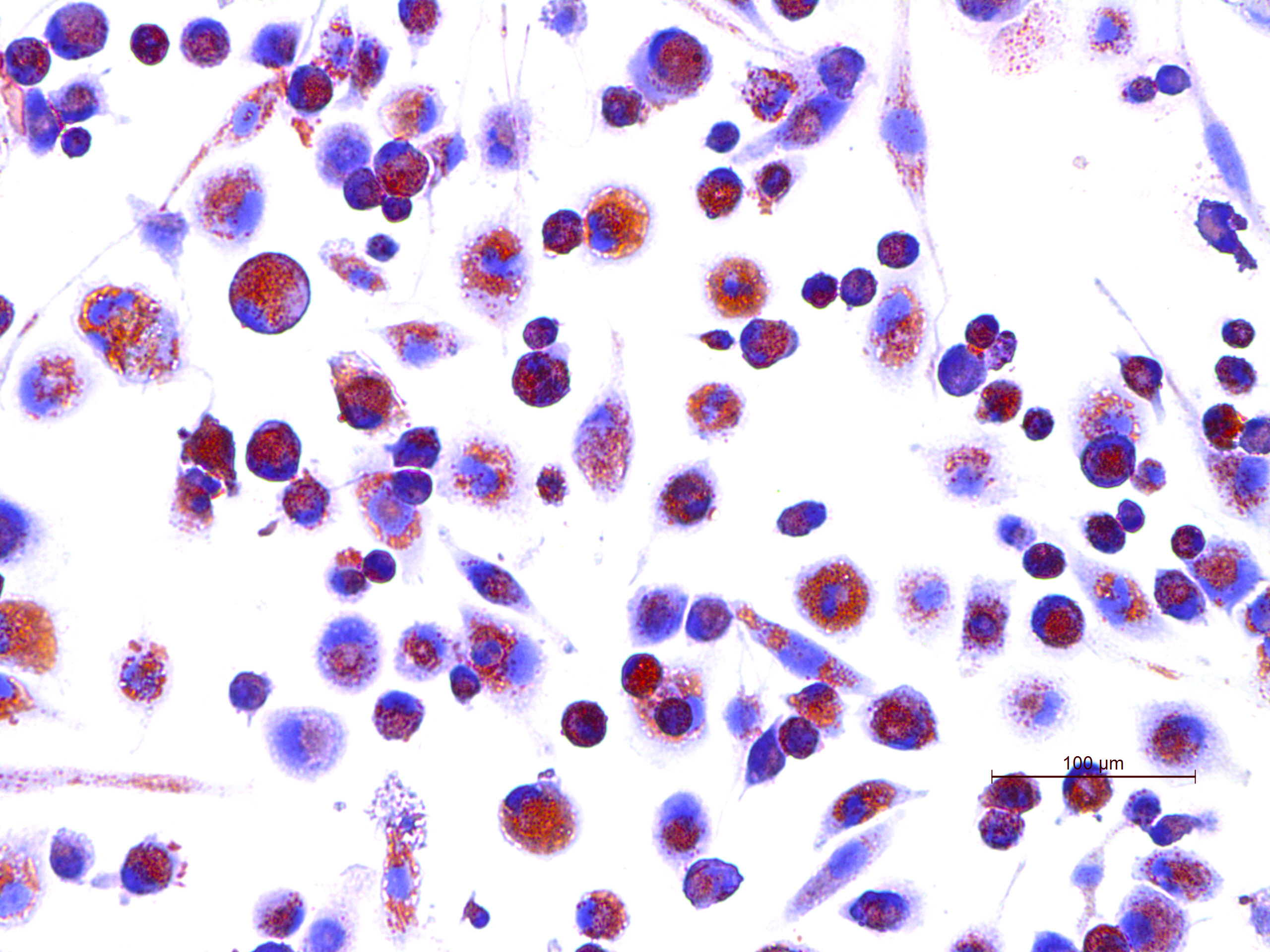

Supplement: S1 File — (ZIP) [file pone.0240762.s001.zip › SI Files Oct 2019/Fig2/oil O stain/Hcy+folate/1.jpg]

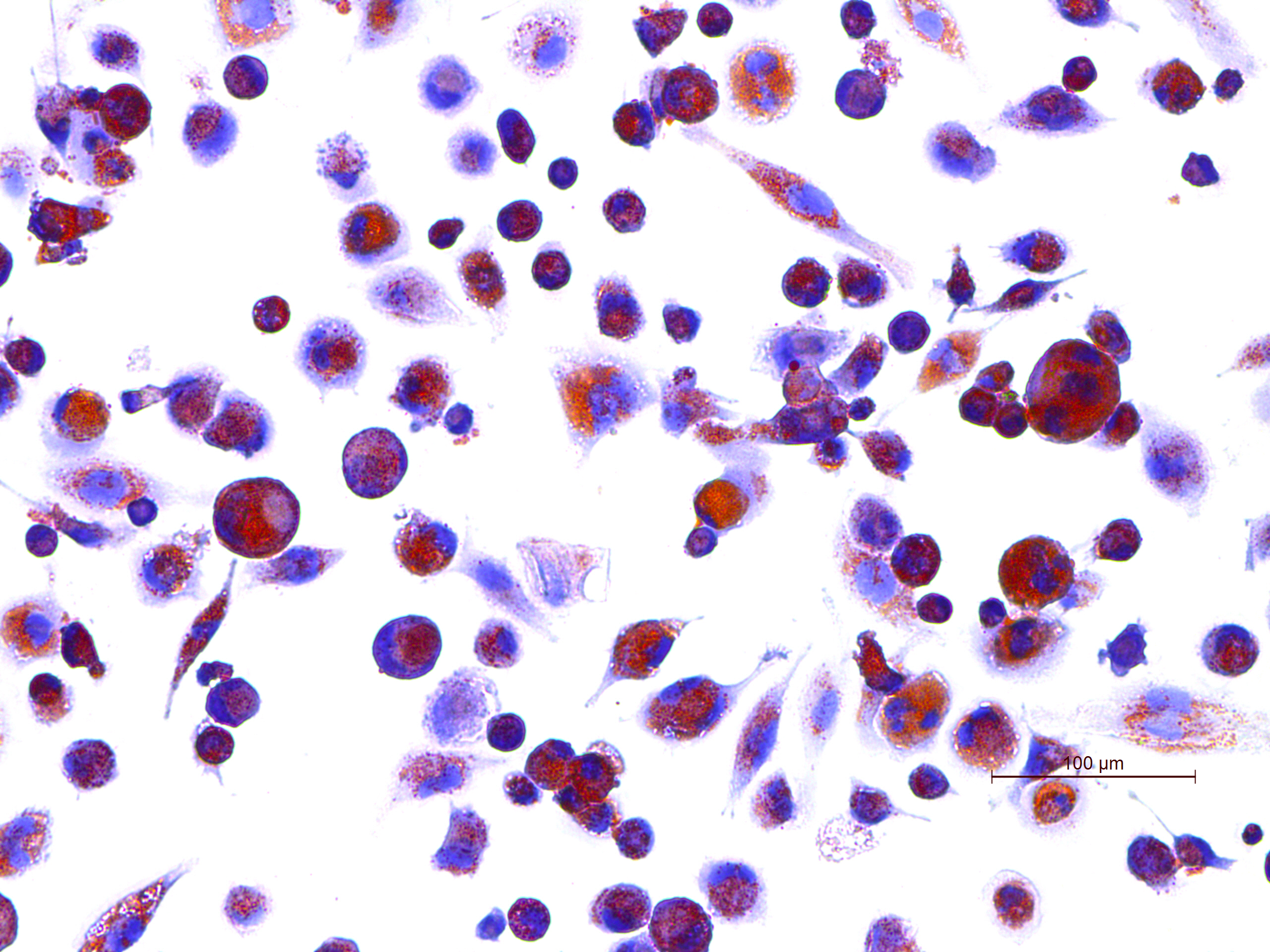

Supplement: S1 File — (ZIP) [file pone.0240762.s001.zip › SI Files Oct 2019/Fig2/oil O stain/Hcy+folate/2.jpg]

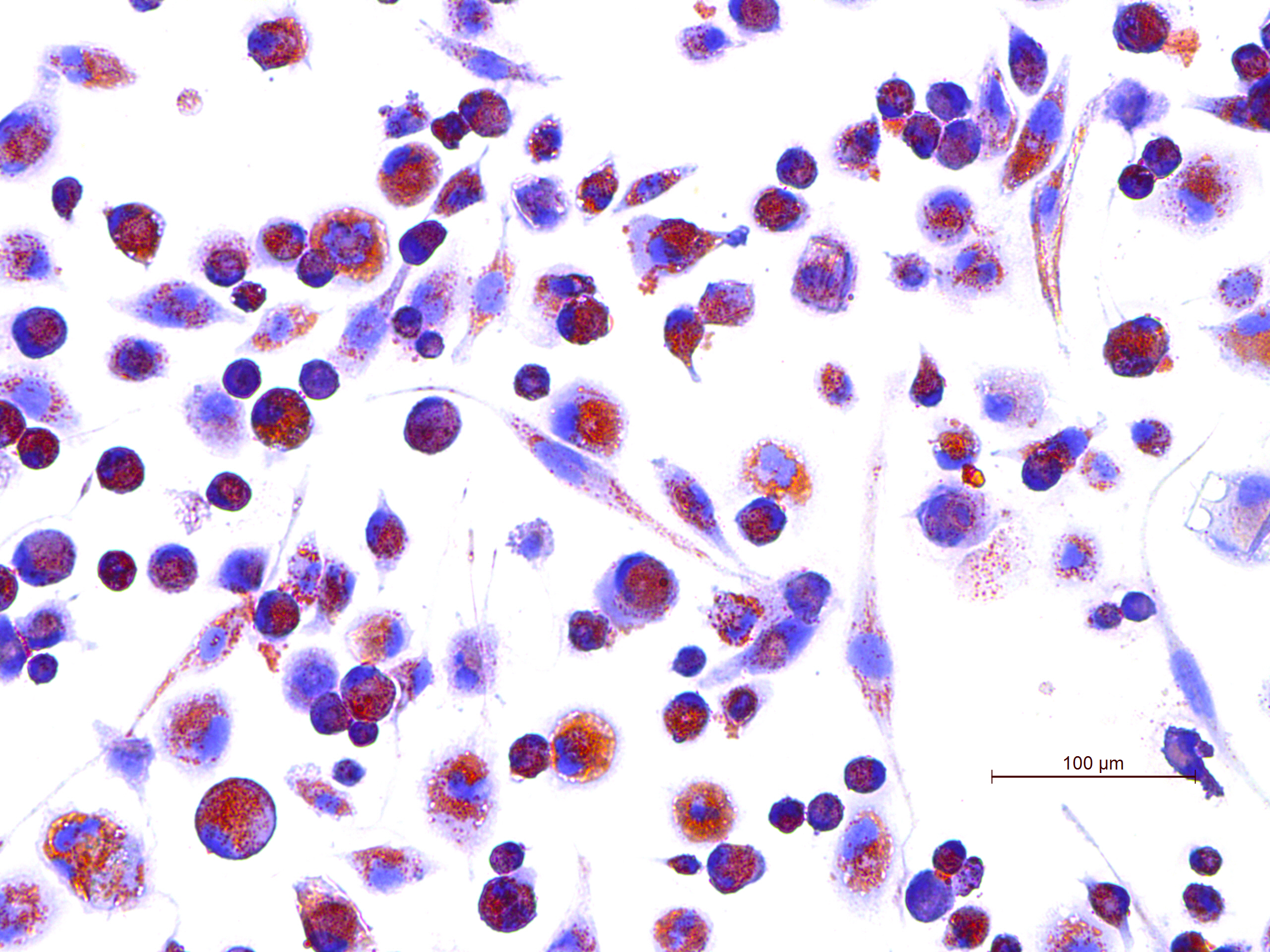

Supplement: S1 File — (ZIP) [file pone.0240762.s001.zip › SI Files Oct 2019/Fig2/oil O stain/Hcy+folate/3.jpg]

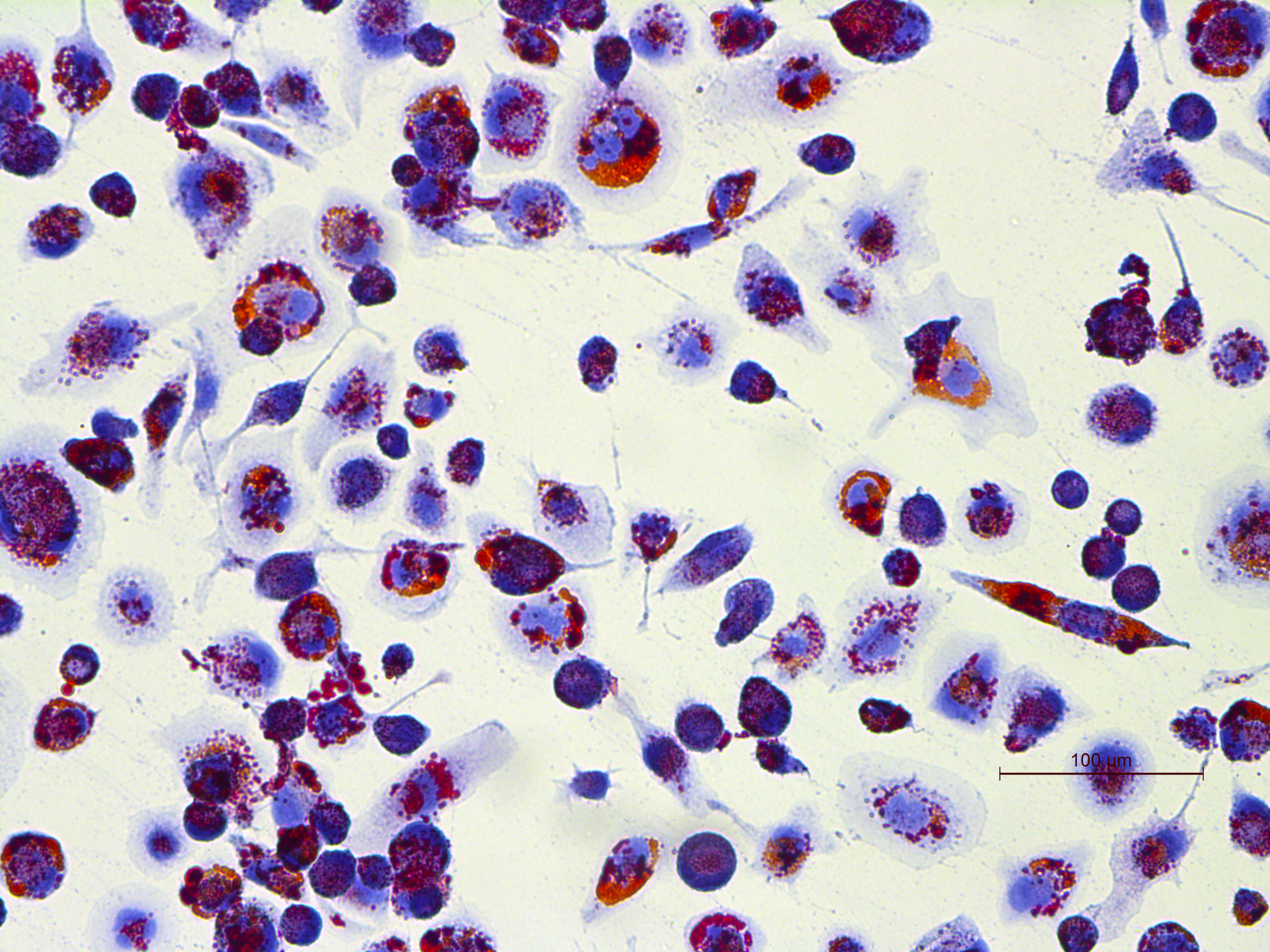

Supplement: S1 File — (ZIP) [file pone.0240762.s001.zip › SI Files Oct 2019/Fig2/oil O stain/Hcy/1.jpg]

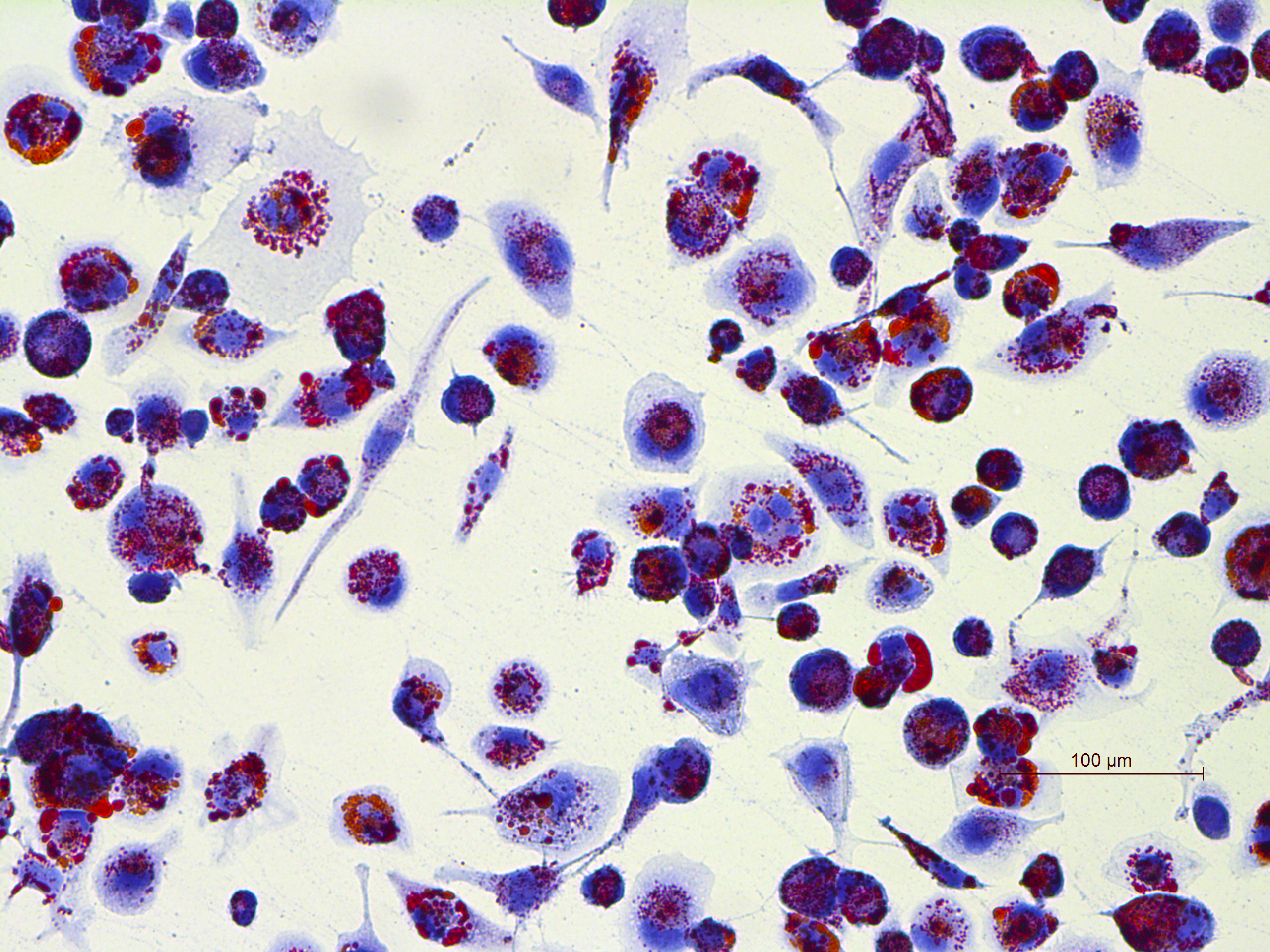

Supplement: S1 File — (ZIP) [file pone.0240762.s001.zip › SI Files Oct 2019/Fig2/oil O stain/Hcy/2.jpg]

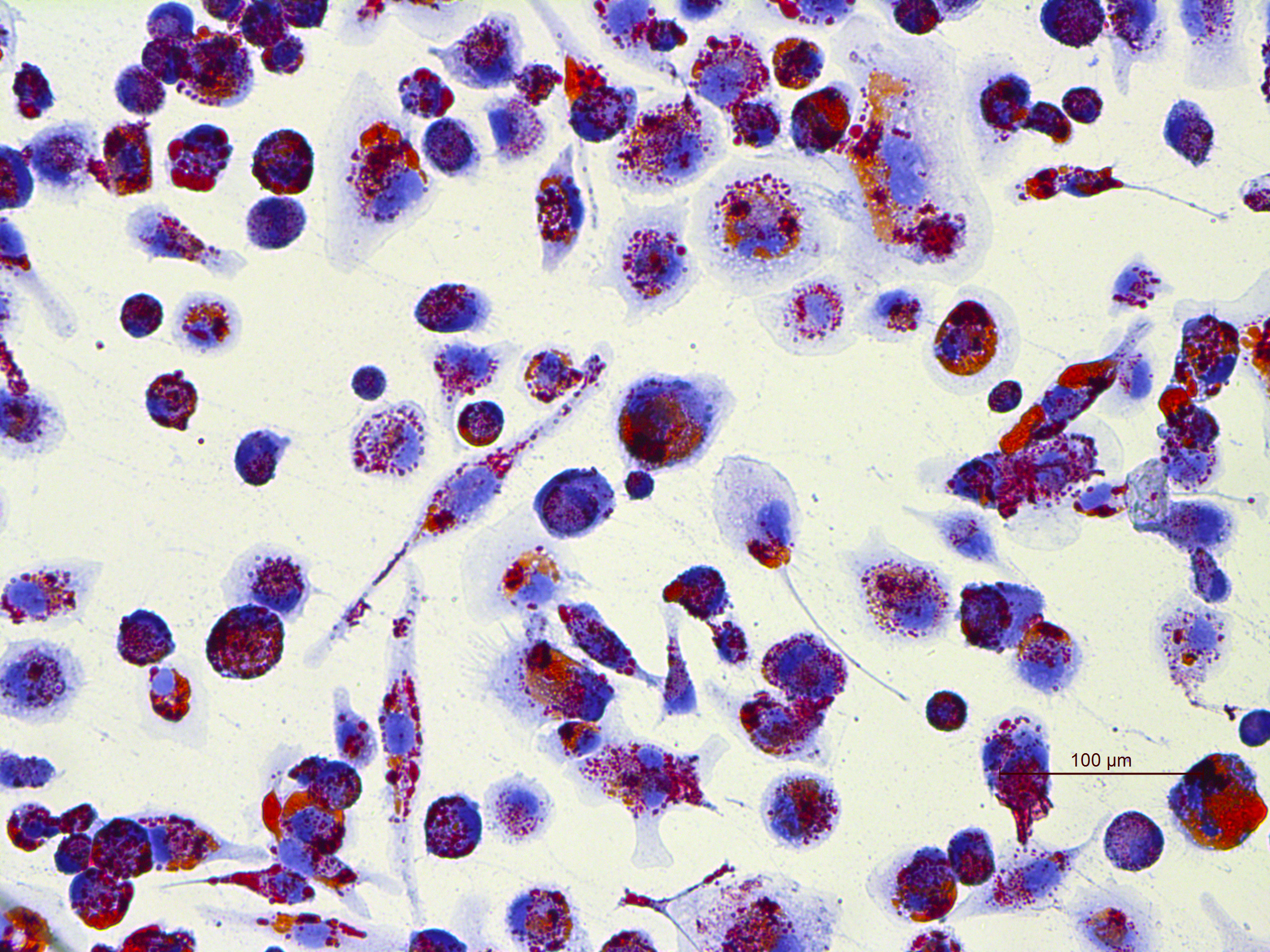

Supplement: S1 File — (ZIP) [file pone.0240762.s001.zip › SI Files Oct 2019/Fig2/oil O stain/Hcy/3.jpg]
